# First Evidence of *Entamoeba* Parasites in Australian Wild Deer and Assessment of Transmission to Cattle

**DOI:** 10.3389/fcimb.2022.883031

**Published:** 2022-06-10

**Authors:** Jose L. Huaman, Carlo Pacioni, Lily Kenchington-Evans, Mark Doyle, Karla J. Helbig, Teresa G. Carvalho

**Affiliations:** ^1^Department of Microbiology, Anatomy, Physiology and Pharmacology, School of Agriculture, Biomedicine and Environment, La Trobe University, Melbourne, VIC, Australia; ^2^Department of Environment, Land, Water and Planning, Arthur Rylah Institute for Environmental Research, Melbourne, VIC, Australia; ^3^Environmental and Conservation Sciences, Murdoch University, Perth, WA, Australia; ^4^Far South Coast, South East Local Land Services, Bega, NSW, Australia

**Keywords:** *Entamoeba bovis*, wild deer, cattle, cross-species infection, Australia, ribosomal lineages, 18S rRNA

## Abstract

Australian wild deer populations have significantly expanded in size and distribution in recent decades. Due to their role in pathogen transmission, these deer populations pose a biosecurity risk to the livestock industry. However, little is known about the infection status of wild deer in Australia. The intestinal parasite *Entamoeba bovis* has been previously detected in farm and wild ruminants worldwide, but its epidemiology and distribution in wild ruminants remain largely unexplored. To investigate this knowledge gap, faecal samples of wild deer and domestic cattle from south-eastern Australia were collected and analysed for the presence of *Entamoeba* spp. using PCR and phylogenetic analysis of the conserved 18S rRNA gene. *E. bovis* parasites were detected at high prevalence in cattle and wild deer hosts, and two distinct *Entamoeba* ribosomal lineages (RLs), RL1 and RL8, were identified in wild deer. Phylogenetic analysis further revealed the existance of a novel *Entamoeba* species in sambar deer and a novel *Entamoeba* RL in fallow deer. While we anticipated cross-species transmission of *E. bovis* between wild deer and cattle, the data generated in this study demonstrated transmission is yet to occur in Australia. Overall, this study has identified novel variants of *Entamoeba* and constitutes the first report of *Entamoeba* in fallow deer and sambar deer, expanding the host range of this parasite. Epidemiological investigations and continued surveillance of *Entamoeba* parasites in farm ruminants and wild animals will be required to evaluate pathogen emergence and transmission to livestock.

## Introduction

Parasites of the genus *Entamoeba* comprise unicellular anaerobic organism that infect humans (Shirley et al., 2018; Cui et al., 2019), domestic animals (Noble and Noble, 1952; Stensvold et al., 2010; Cui et al., 2019; Ai et al., 2021) and wild animals (Stensvold et al., 2010; Shilton et al., 2018; Cui et al., 2019). *Entamoeba* parasites develop through a faecal-oral life cycle, and infections with pathogenic species can cause intestinal disease and damage the liver and brain (Ngui et al., 2012). The initial classification of *Entamoeba* species was established based on the type of host in which the parasites were identified (Noble and Noble, 1952) and on parasite morphological features (Stensvold et al., 2011), such as cyst size and the number of nuclei. Using this approach, *Entamoeba* species have been classified into four distinct groups, including *E. gingivalis*-like group (species without cysts), *E. bovis*-like group (uni-nucleated cysts), *E. histolytica*-like group (quadri-nucleated cysts), and *E. coli*-like group (octo-nucleated cysts) (Clark et al., 2006; Stensvold et al., 2011). In recent years, the analysis of *Entamoeba* 18S ribosomal RNA (18S rRNA) sequences has significantly expanded the repertoire of genetically distinct *Entamoeba* organisms (Clark et al., 2006; Stensvold et al., 2011; Jacob et al., 2016). Although morphology-based analysis will be required to consolidate such findings, they provide unique insights into variation within species, evolutionary relationships, and host specificity (Clark et al., 2006; Stensvold et al., 2011; Jacob et al., 2016). Moreover, analysis of *Entamoeba* DNA sequences is an essential tool in endemic countries where microscopy does not allow for the distinction of pathogenic and non-pathogenic *Entamoeba* species (Nath et al., 2015). The genetic diversity of morphologically identical parasites, and the host promiscuity of *Entamoeba* organisms, highlights an ongoing need for further characterisation of genetic variants and host range, particularly in pathogenic species and emerging zoonotic species infections.

In animals, ruminants such as cattle and sheep appear to be common hosts of the uni-nucleated cyst *Entamoeba* species (Noble and Noble, 1952; Clark et al., 2006; Stensvold et al., 2010). Nevertheless, cyst morphology varies greatly within and between uni-nucleated cyst-producing species isolated from different ruminant hosts (Stensvold et al., 2010). The term “ribosomal lineage” (RL) was introduced to name newly discovered *Entamoeba* 18S rRNA sequences with greater than 5% divergence from known species. These RLs represent organisms not yet described morphologically and not referrable to described species (Jacob et al., 2016). The analysis of *Entamoeba* 18S rRNA sequences detected in farmed and wild ruminants over the last decade revealed the presence of *E. bovis* and eight RLs. Of these, four RLs are closely related to *E. bovis* (*Entamoeba* RL 1-3 and 8) (Jacob et al., 2016). Besides being detected in cattle (*Bos taurus*) (Stensvold et al., 2010; Jacob et al., 2016; Nolan et al., 2017; Matsubayashi et al., 2018; Ai et al., 2021) and sheep (*Ovis aries*) (Stensvold et al., 2010; Jacob et al., 2016; Ai et al., 2021), *E. bovis* and *Entamoeba* RLs have also been detected in goats (*Capra hircus*) (Nolan et al., 2017; Al-Habsi et al., 2017; Ai et al., 2021), horses (*Equus ferus*) (Ai et al., 2021), camels (*Camelus ferus*) (Ai et al., 2021), and cervids. Among the studies conducted on cervids, white-tailed deer (*Odocoileus virginianus*) from the USA (Kingston and Stabler, 1978), fallow deer (*Dama dama*) from Mauritius (Jacob et al., 2016), and reindeer (*Rengifer tarandus*) from Iceland (Stensvold et al., 2010) tested positive for *E. bovis*, while *Entamoeba* RL 1 was detected in roe deer (*Capreolus capreolus*) from Sweden (Stensvold et al., 2010). Information about the pathogenicity of *E. bovis* and *Entamoeba* RLs in ruminants remains limited; however, their detection in cattle in the absence of clinical symptoms such as diarrhoea, suggests low pathogenicity (Matsubayashi et al., 2018; Ai et al., 2021). *E. bovis* have a broad ruminant host range and can be transmitted by faecal excretion of cysts followed by oral ingestion of contaminated food or water (Noble and Noble, 1952; Clark et al., 2006; Stensvold et al., 2010). Based on the oral-faecal life cycle of *Entamoeba* parasites, the transmission of *E. bovis* between different host taxa that share common land is highly likely.

To date, *Entamoeba* parasites have only been identified twice in Australian wild animals. *E. ranarum* was detected and characterised in wild cane toads (*Rhinella marina*) (Shilton et al., 2018), and *E. bovis* was detected in feral goats in Western Australia rangeland with a 6.4% prevalence (Al-Habsi et al., 2017). The prevalence and distribution of *Entamoeba* species in Australian farmed and wild ruminants, such as wild deer, remains yet to be investigated. Wild deer and livestock commonly share grazing areas in agricultural landscapes and are equally susceptible to a wide range of pathogens of agricultural importance (Cripps et al., 2019). Wild deer represent a significant source of pathogen transmission; thus, we hypothesised wild deer to be involved in the transmission of *Entamoeba* parasites to livestock and *vice versa*. In recent years, our team has investigated the role of wild deer as carriers of livestock pathogens in Australia (Huaman et al., 2020; Huaman et al., 2021; Huaman et al., 2021a; Huaman et al., 2021b), and here, we report the first detection of *Entamoeba* parasites in wild deer sampled in Australia. Further, we assess the prevalence, distribution, and characterisation of *Entamoeba* species and RLs as well as the potential of cross-species transmission.

## Materials and Methods

### Sample Collection

**Figure 1:**
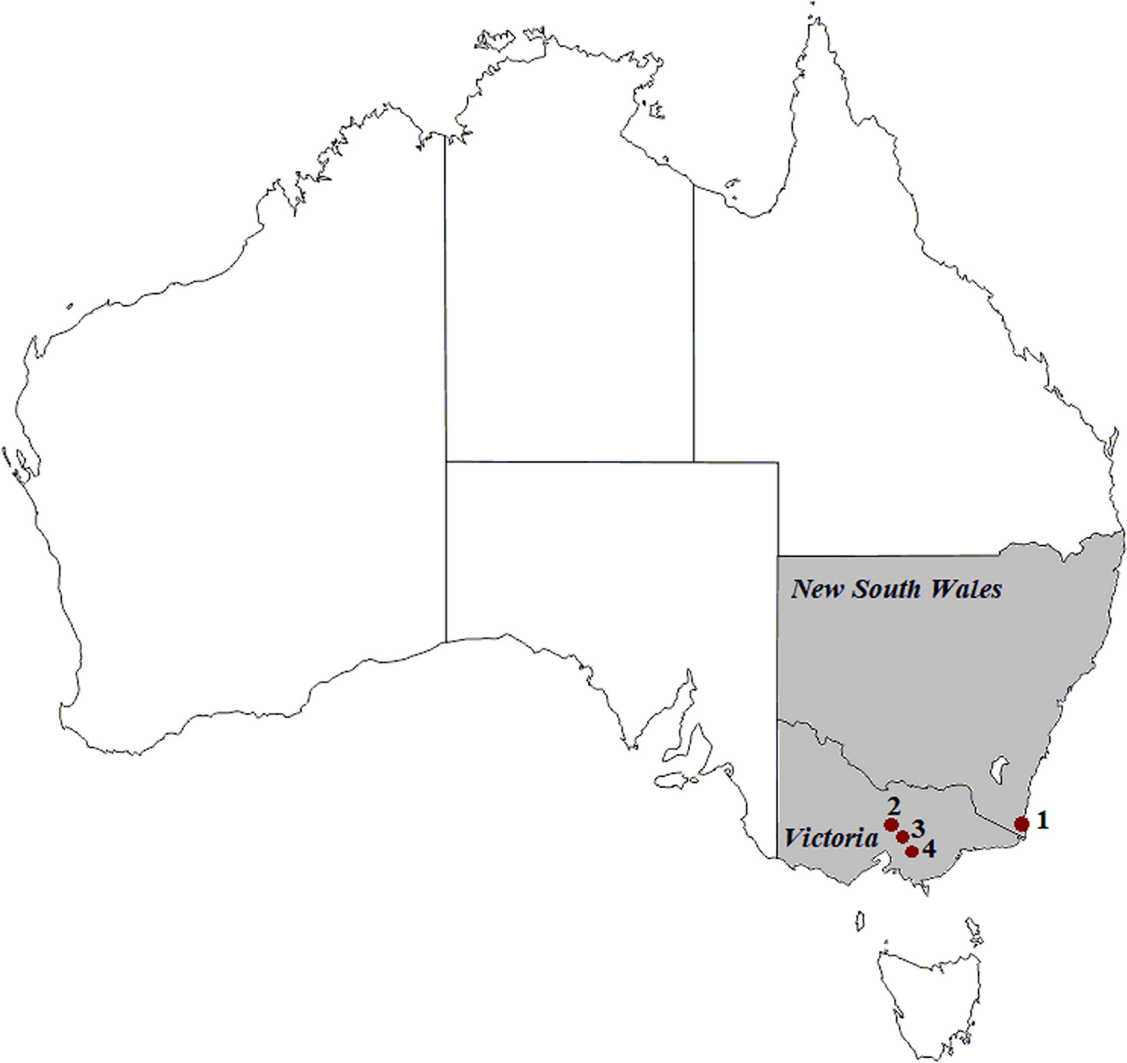
Geographic locations of deer (1 to 4) and cattle (1, 2 and 4) sample collection in south-eastern Australia. (1) Kiah, (2) Outer Melbourne, (3) Yellingbo, (4) Bunyip. ^©^d-maps.com.

Faecal samples were collected from Australian wild deer to assess their infection status (Huaman et al., 2021a; Huaman et al., 2021b). Opportunistic sampling during field necropsies was carried out on deer culled with the assistance of recreational and professional hunters as part of control operations in New South Wales and Victoria (**Figure 1**) between August 2019 and October 2020. All samples were collected from the large intestine and placed in sterile plastic containers (Techno Plas, Australia).

Cattle faecal samples collected for clinical investigations independent from this study were analysed here for the presence of *Entamoeba* parasites. Cattle samples were collected from beef and dairy farms within a 20 kilometres radius of the deer sampling areas between September 2020 and April 2021. All samples were collected directly from the animals, placed in individual sterile plastic containers and immediately refrigerated. Samples were transported to the Laboratory of Molecular Parasitology within the Department of Microbiology, Anatomy, Physiology and Pharmacology at La Trobe University, and stored at -80° C until further use.

### DNA Extraction and PCR Amplification

Frozen faecal samples were aliquoted into 0.25 g frozen portions. Genomic DNA was extracted using a DNeasy^®^ PowerSoil^®^ Kit (Qiagen, Valencia, CA, USA) following the manufacturer’s instructions. PCR was performed with primers EntboF2 and EntboR3 (Matsubayashi et al., 2018; Ai et al., 2021) to amplify an internal fragment of 850 bp of the 18S rRNA *Entamoeba* gene. In addition, the methodology published by Ali et al. (2005) was employed to detect tRNA-linked short tandem repeats (STRs). Six previously published primer pairs (A-L5/A-L3, D-A5/D-A3, N-K5/N-K3, R-R5/R-R3, S^TGA^-D5/S^TGA^-D3, S-Q5/S-Q3) were selected and tested in all the deer and cattle samples. These primers were originally designed to amplify *E. hystolytica* t-RNA STRs. PCR amplification was performed in a 25 μL reaction mixture containing 1x Green GoTaq Flexi buffer, 2 mM of MgCl_2_, 10 mM of dNTPs, 0.2 μM of each primer, 0.625 units of GoTaq G2 DNA polymerase (Promega, Madison, WI, USA), and 1 μL of template DNA. Amplification was carried out in a T100 thermal cycler (BioRad, Hercules, CA, USA), and amplification products were visualised by gel electrophoresis, using a 2% agarose gel stained with RedSafe™ (iNtRON Biotechnology, Gyeonggi-do, Korea), and the high-resolution ChemiDoc™ MP Imaging System (Bio-Rad, Hercules, CA, USA).

### DNA Sequencing and Phylogenetic Analysis

PCR products were sequenced by bi-directional Sanger sequencing at the Australian Genome Research Facility (Melbourne, Australia), then analysed and edited using Geneious software 11.1.4 (Biomatters Ltd., Auckland, New Zealand, version 11.1.4). Multiple sequence alignments were performed using Clustal X (Thompson et al., 1997). A phylogenetic tree was built based on *Entamoeba* 18S rRNA sequences using the substitution model with the lowest BIC scores (Tamura 3-parameter model + G) and the maximum-likelihood method in MEGA 7 (Kumar et al., 2016). Thus, *Entamoeba* sequences obtained in the present study were aligned with 31 *Entamoeba* reference sequences deposited in GenBank (**Table S1**). These reference sequences represented 17 recognised species and 5 published ribosomal lineages. Statistical support for the trees was evaluated by bootstrapping based on 1,000 repetitions. Moreover, the number of nucleotide differences and the mean sequence divergence of *Entamoeba* clades identified in our sequences were calculated in MEGA 7. The nucleotide 18S rRNA sequences detected in this study were submitted to GenBank under accession number OM415364 - OM415424 (**Table S2**).

### Bayesian Divergence Time Estimates

As deer were introduced in Australia only 200 years ago, estimating the most recent common ancestors (TMRCA) of *E. bovis* detected in wild deer and cattle can reveal whether parasite transmission occurred between the two hosts in Australia. Therefore, the reported split ages (Romero et al., 2016) between *E. nuttalli* and *E. hystolytica* (5.93 ± 0.28Mya), along with *E. hystolytica* and *E. invadens* (68.18 ± 16.04 Mya), were used as calibrations for the Bayesian analysis using a lognormal distribution with a mean of 1.78 and 4.5, and a standard deviation of 0.05 and 0.25, respectively. The phylogenetic trees were modelled using a birth-death tree prior, a lognormal relaxed clock in BEAST v2.6.3 (Bouckaert et al., 2019), and a gamma distribution (shape=1, rate=0.00001) for the substitution rate parameter. Two independent runs of 200 million steps were computed, sampling parameters every 10,000 steps and discarding the first 10% of each chain as burn-in. Tracer v1.7.1 (Rambaut et al., 2018) was used to ensure that the length of the burn-in phase was sufficient and guaranteed convergence of the two analyses. Results were obtained after combining the two chains with LogCombiner. The programs TreeAnnotator v2.6.2 (Bouckaert et al., 2019) and FigTree v1.4.4 (http://tree.bio.ed.ac.uk/software/figtree/) were used to summarise the posterior tree distribution and visualise the annotated Maximum Clade Credibility (MCC) tree.

## Results

### High Prevalence of Entamoeba DNA Found in Wild Deer and Cattle Samples

A total of twenty-three cattle faecal samples were obtained from south-eastern Australia, as well as seventy-one wild deer faecal samples, including sixty samples from fallow deer (*Dama dama*) and eleven samples from sambar deer (*Rusa unicolor*) (**Table 1**). All samples were screened by PCR for the presence of the 18S rRNA *Entamoeba* gene using primers EntboF2 and EntboR3 (Matsubayashi et al., 2018). The overall prevalence of *Entamoeba* spp. in wild deer from south-eastern Australia was found to be 81.7% (58/71), ranging from 72.9% to 100% depending on the host species and the sample geographic location (**Table 1**). In the cattle faecal samples, the prevalence of *Entamoeba* spp. was 100% (**Table 1**). All the *Entamoeba* 18S rRNA PCR amplicons generated (seventy-one from wild deer samples and twenty-three from cattle samples) were analysed by Sanger sequencing. Subsequent analysis of the ninety-four 18S rRNA sequences revealed *E. bovis* as the dominant species detected with a total prevalence of 74.6% (53/71) in wild deer and 100% (23/23) in cattle.

**Table 1:** *Entamoeba* species and RLs identified in deer and cattle faecal samples collected across south-eastern Australia.

Host species	Geographic location	Total	PCR positive (%)	*Entamoeba* species (*n*)
Fallow deer	NSW	48	35 (72.9)	*E. bovis* (33), *Entamoeba* RL 8 (1), *Entamoeba* RL^a^ (1)
	VIC	12	12 (100)	*E. bovis* (11), *Entamoeba* spp^b^ (1)
Sambar deer	VIC	11	11 (100)	*E. bovis* (9), *Entamoeba* RL 1 (1), *Entamoeba* RL^a^ (1)
Cattle	NSW	15	15 (100)	*E. bovis* (23)
	VIC	8	8 (100)	

NSW, New South Wales; VIC, Victoria; ^a^ novel Entamoeba RL, ^b^ novel Entamoeba species.

### Phylogenetic Analysis of Entamoeba Sequences Reveals the Existence of RL Variants in Wild Deer Samples

**Figure 2:**
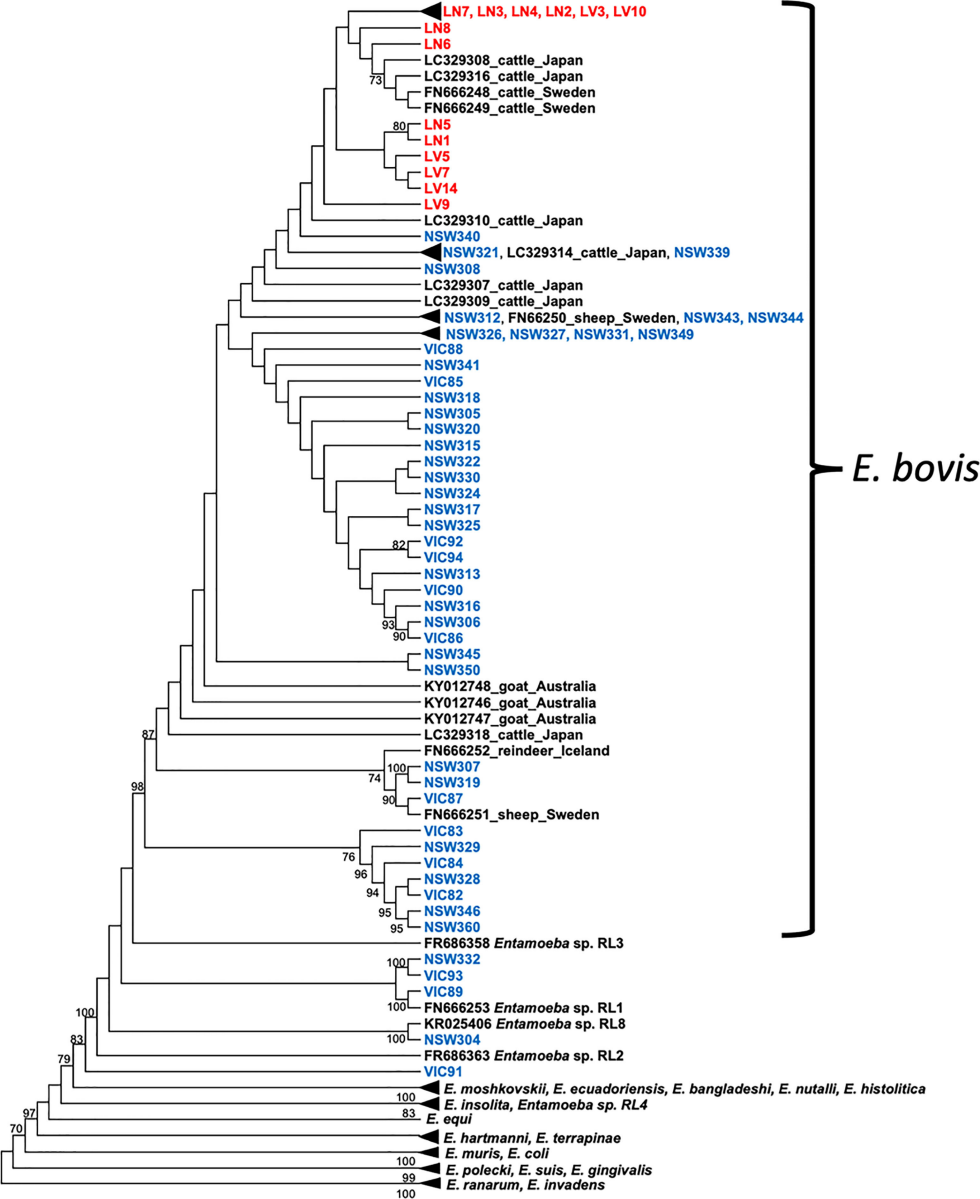
Cladogram of *Entamoeba* partial 18S rRNA sequences. Deer sequences are indicated in blue and cattle sequences in red. Reference sequences are indicated in black. The tree was constructed using the maximum likelihood method and Tamura 3-parameter + G substitution model. Bootstrap values above 70% are shown at the nodes. Note: substitutions do not scale branches in this tree. The phylogenetic tree with scaled branches and alignment is shown in **Figures S2**, **S3**, respectively.

Out of the 850 bp *Entamoeba* 18S amplicons generated by PCR, a good quality DNA fragment of 778 bp was successfully sequenced for each of the ninety-four deer and cattle samples. This 778 bp fragment covered nearly 50% of the *Entamoeba* 18S rRNA gene and was therefore used to investigate the phylogenetic relationship and the levels of divergence of the ninety-four *Entamoeba* sequences. A high proportion of the wild deer-derived sequences fell into the *E. bovis* clade, which includes isolates from rangeland goats, cattle, sheep, and reindeer (**Figure 2**). Further, the cattle-derived sequences clustered exclusively within the *E. bovis* clade. The genetic similarity among all *E. bovis* sequences obtained in this study (both derived from deer and cattle) ranged from 92.5% to 100%. Moreover, the mean divergence within the cattle-derived sequences is 5 to 7-fold smaller than the divergence observed within the deer-derived sequences (**Table 2**). Within the 778 bp 18S rRNA gene fragment, a mean of 21 nucleotide differences was found between the two host group sequences (**Table 2**).

**Table 2:** Mean sequence divergence and number of differences (nucleotides) between Australian deer and cattle sequences within clades.

Clades	Sequence divergence %	Number of differences
*E. bovis* deer vs *E. bovis* cattle	2.9 % ± 0.4 %	20.88 ± 2.8
Within *E. bovis* deer	3.5 % ± 0.4 %	24.32 ± 2. 8
Within *E. bovis* cattle	0.7 % ± 0.2 %	5.06 ± 1.3
All deer vs all cattle	4.2 % ± 0.5 %	27.72 ± 3
within all deer	5.8 % ± 0.6 %	36.70 ± 3.1
within all cattle	0.7 % ± 0.2 %	5.06 ± 1.3
Non-VIC91 deer vs VIC91	26.2 % ± 2.9 %	133.76 ± 10

Interestingly, five 18S rRNA sequences of deer origin (VIC89, NSW304, NSW332, VIC93 and VIC91) clustered with distinct *Entamoeba* RLs, while none of the sequences identified in this study clustered with *Entamoeba* RLs 2, 3 and 4, which were previously reported in ruminants (**Figure 2**). Sequence VIC89, sourced from sambar deer, clustered with *Entamoeba* RL1 (FN666253) detected in roe deer from Sweden and shared 98.6% of the nucleotide sequence. Sequence NSW304 sourced from fallow deer, clustered with *Entamoeba* RL8 (KR025406), detected in cattle from the United Kingdom, with a homology of 99.9%. Comparison of sequence NSW304 with two additional *Entamoeba* RL8 sequences detected in camel (MN749974) and goat (MN749989) from China revealed a nucleotide identity of 99.9% and 95.1%, respectively. The alignment of these four *Entamoeba* RL8 sequences (NSW304, FN666253, MN749974, and MN749989) revealed an identity of 100% between sequences NSW304, FN666253, and MN749974; while three insertions (at positions 715, 716 and 750) and one deletion (at position 677) were identified in the strain detected in the goat (MN749989) (**Figure S1**). Sequences VIC93 and NSW332 sourced from fallow deer fell into the same clade, displaying 99% sequence identity; however, these two sequences did not cluster with any *Entamoeba* RL reference sequence, therefore emerging as a divergent *Entamoeba* RL (**Figure 2**). Sequence VIC91 obtained from sambar deer was genetically distinct from the *Entamoeba* sequences identified in other deer and cattle samples with high sequence divergence (mean 26.2%) and nucleotide difference (mean 133.76) (**Table 2**). Nucleotide similarity between sequence VIC91 and the reference *Entamoeba* sequences ranged from 82% to 86%. Overall, these findings suggest sample VIC91 belongs to a novel *Entamoeba* species.

### STRs Were Amplified in Deer and Cattle Samples But Not Successfully Sequenced

Amplicons were obtained for all the STRs tested except for D-A5/D-A3 (**Figure S4**), albeit a slight difference in size when compared to amplicons of *E. histolytica* (Ali et al., 2005). A total of ten samples, including 4 wild deer samples and 6 cattle samples (**Figures S4A, B**, respectively) were sequenced using primers S^TGA^-D5 and S^TGA^-D3 (Ali et al., 2005). However, DNA sequences of good quality could not obtained, even when cloning the STR amplicons prior to sequencing.

### Divergence Time Analysis Suggests Lack of Entamoeba Transmission Between Wild Deer and Cattle

**Figure 3:**
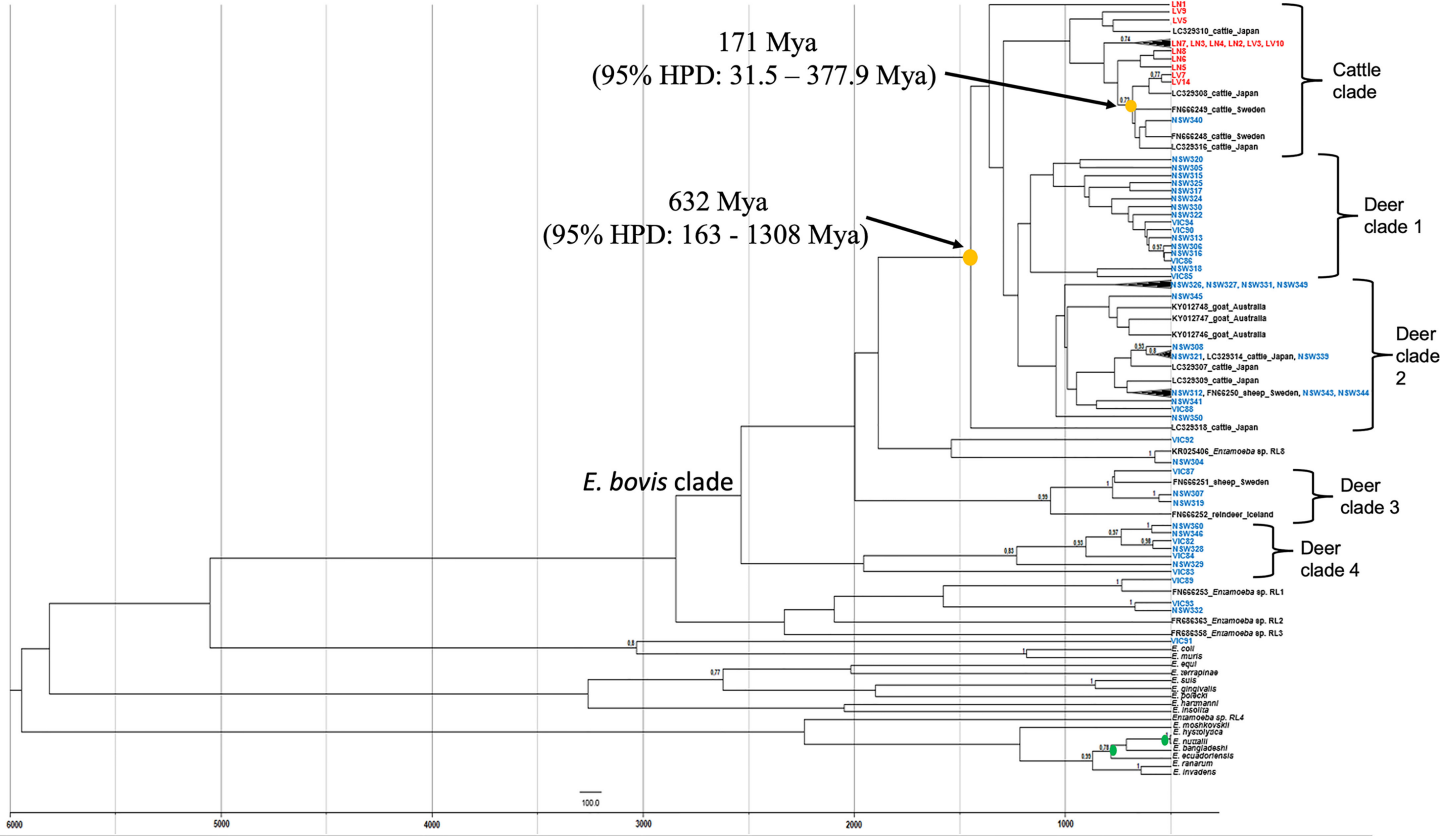
Maximum clade credibility tree of *Entamoeba* obtained from Bayesian inference using split ages reported previously as calibrations (green dots). Yellow dots indicate the estimated mean ages for the most recent common ancestor (TMRCA) of *Entamoeba* detected in Australian wild deer and cattle. Deer sequences are indicated in blue and cattle sequences in red. Reference sequences are indicated in black. HPD, highest posterior density, Mya, Million years ago.

To determine the transmission of *Entamoeba* parasites between wild deer and cattle in this study, a phylogenetic tree was constructed using a birth-death tree prior under a Bayesian framework and two calibration nodes (**Figure 3**). This approach aims to reconstruct the speciation process and, by using the time of divergence between two taxa as calibration, it converts the unit of the branches from substitutions to time (years in this case). The trees explored are then annotated in a maximum clade credibility (MCC) tree. The MCC tree revealed a clear species structure with the cattle-derived *Entamoeba* sequences well separated from the wild deer-derived *Entamoeba* sequences, like the previously generated maximum likelihood (ML) tree (**Figure 2**). Sequence NSW340 was, however, an exception to the species separation as it clustered with LV7 and LV14 (**Figure 3**). Overall, sequences sourced from wild deer clustered within four clades (**Figure 3**). There was little resolution within the deer group, as reflected in the low node posterior probabilities (< 0.7); however, deer clade 2 and 3 grouped with posterior probabilities > 0.8, and similar strong support was found in the ML tree. The MCC tree confirmed that sequences VIC89 and NSW304 belong to *Entamoeba* RL1 and RL8, respectively, and corroborated that sequence VIC91 is genetically distinct from the other sequences analysed in this study. In the ML tree sequences, LN1 (cattle origin) and VIC92 (deer origin) clustered within the *E. bovis* clade, although they fell outside of any other cluster, which is in contrast with the output of the ML tree (**Figure 2**). The most recent node between sequences obtained from a deer and a cow in Australia was estimated to be 171 million years ago (Mya) (95% HPD: 31.5 – 377.9 Mya). While the most recent common ancestors (TMRCA) between Australian wild deer and cattle clades was estimated at 632 Mya, but with considerable uncertainty (95% HPD: 163 - 1308 Mya) (**Figure 3**).

## Discussion

In recent decades, Australian wild deer populations have significantly increased in abundance and distribution, leading to regular close interactions between deer and livestock, which increases the risk of pathogen transmission (Davis et al., 2016; Cripps et al., 2019). However, little is known about the epidemiology of pathogens that Australian deer may transmit to livestock, other domestic animals, or wildlife. The present study complements our initial work on investigating pathogens in wild deer across multiple geographic locations in Australia (Huaman et al., 2020; Huaman et al., 2021; Huaman et al., 2021a; Huaman et al., 2021b). Here we report the identification of *Entamoeba* sequences in wild deer and cattle faecal samples collected in south-eastern Australia, with subsequent phylogenetic analysis to evaluate the cross-species transmission of *Entamoeba* parasites. This baseline information is of value for monitoring the status of parasitic infections in Australian deer and evaluating the risk of disease transmission between wild deer and livestock. Additionally, the data provided by this study increases our knowledge of the host range and distribution of *Entamoeba*, a group of parasites prevalent in ruminant livestock. Finally, this study represents the first molecular screening and characterisation of *Entamoeba* in Australian wild deer.

The predominant *Entamoeba* species identified in the wild deer and cattle samples collected and analysed in this study was *Entamoeba bovis*, a species recognised to infect ruminants, including livestock animals (Stensvold et al., 2010; Matsubayashi et al., 2018; Ai et al., 2021). The prevalence of *Entamoeba* infections previously reported in cattle are relatively low [2.5% in Costa Rica (Jimenez et al., 2007), 4.8% in Korea (Ismail et al., 2010)] when detected by microscopic analysis; while higher prevalences have been reported following PCR analysis [72% in Japan (Matsubayashi et al., 2018), 80% in Uganda (Nolan et al., 2017), 100% in China (Ai et al., 2021)]. Similar to these last reports, the present study detected a prevalence of 100% for *E. bovis* in cattle samples (n=23) using a PCR-based analysis.

*E. bovis* has also been previously detected in wild cervids, including in wild goats from Western Australia with a prevalence of 6.4% (Al-Habsi et al., 2017). This relatively low *E. bovis* prevalence contrasts with the much higher prevalence of 74.6% (53/71) reported here in fallow deer and sambar deer from eastern Australia. This difference could be attributed to methodology, as *E. bovis* was identified in wild goats from Western Australia (Nolan et al., 2017) by microscopy analysis. Indeed, microscopy and morphology-based detection methods are likely to underestimate parasite prevalence, as discussed above for the case of cattle samples, and are less sensitive methods when compared to the molecular detection tools employed in the present study. Although microscopy detection methods might underestimate the number of *E. bovis* infections, it is not excluded that lower parasite prevalence can exist, for example, due to climatic reasons. The sampling areas of this study are located in south-eastern Australia, where a Mediterranean-like climate prevails with significantly humid winters, which can facilitate the maintenance of parasites in the environment (Shirley et al., 2018). In contrast, the sampling area of Al-Habsi et al. (2017) was the semiarid rangeland area in Western Australia.

Wild deer and cattle-derived *Entamoeba* 18S rRNA sequences cluster within the *E. bovis* clade, although different species-specific clades are formed (**Figure 3**). Pairwise analysis revealed differences within sequences of wild deer origin, indicating high parasite diversity within this host. Polymorphic markers such as serine-rich protein genes and tRNA-linked short tandem repeats (STRs) have been used for the genotyping and correlation with the geographical distribution of other *Entamoeba* species such as *E. histolytica, E. dispar*, and *E. nuttalli* (Tawari et al., 2007; Weedall and Hall, 2011; Feng et al., 2014). However, this approach is yet to be used to identify *E. bovis*. We employed a methodology previously used in *E. histolytica* (Ali et al., 2005); however, the sequencing of ten samples (4 wild deer and 6 cattle samples) using primers S^TGA^-D5 and S^TGA^-D3 did not generate good quality DNA sequences, even when STR amplicons were cloned prior to sequencing. The primers used here were originally designed to amplify *E. hystolytica* t-RNA STRs (Ali et al., 2005). Therefore, the presence of polymorphisms in the t-RNA gene of *E. bovis* and/or the sensitivity of the primers could account for the low quality of the sequences generated.

In the absence of STR data and to determine whether a potential *E. bovis* cross-species transmission was possible, the time of the most common ancestor between *E. bovis* sequences of wild deer and cattle origin was estimated (**Figure 3**). The most common ancestor of *E. bovis* identified in wild deer and cattle hosts was estimated to have existed well before 200 years ago (before cattle and deer were introduced in Australia). Both ML and Bayesian phylogenetic analyses grouped the sequences according to their host species with moderate sequence divergence. Therefore, taken together, these results provide no evidence of *E. bovis* transmission between wild deer and cattle in Australia. This finding was somewhat unexpected, but it is possible that since wild deer populations have only recently increased in density, they did not play an important role in the transmission of these parasites thus far. However, this may change in the future due to deer density expansion, increasing contact rates (direct or indirect) and transmission events with livestock species.

Phylogenetic analysis of all *Entamoeba* sequences of deer origin identified that two sequences (VIC89 and NSW304) cluster with two distinct *Entamoeba* RL. The term “ribosomal lineage” (RL) was proposed to name *Entamoeba* strains with greater than 5% sequence divergence from known species (Jacob et al., 2016). Sequence VIC89 detected in a sambar deer from Victoria clustered with high genetic similarity with an *Entamoeba* RL1 sequence from a roe deer from Sweden (FN666253). This RL was also detected in one gazelle and one bighorn sheep in the USA; however, their sequences are not available for comparison (Jacob et al., 2016). Sequence NSW304, detected in a fallow deer from NSW, clustered with an *Entamoeba* RL8 sequence (KR025406). This ribosomal lineage has been previously identified in a variety of hosts, including cow (Jacob et al., 2016), camel (Ai et al., 2021) and goat (Ai et al., 2021). The sequence NSW304 presents high homology with an RL8 sequence of a cow (99.9%; KR025406) and camel (99.9%; MN749974) origin and lower homology with a sequence of goat origin (95.1%; MN749989). Here we present the first identification of *Entamoeba* RL1 and *Entamoeba* RL8 sequences in wild deer. These results broaden the host range of both RLs.

Interestingly, *Entamoeba* sequences (NSW332, VIC93 and VIC91) detected in three wild deer animals did not cluster with any 18S rRNA reference sequence. One sambar deer-sourced sequence (VIC91) was genetically distinct from other *Entamoeba* species found in ruminants, suggesting a possible novel *Entamoeba* species. Sequences NSW332 and VIC93 of fallow deer origin displayed 100% identity with each other and clustered as a sister clade with the VIC89/*Entamoeba* RL1 clade. Jacob et al. (2016) proposed the classification for RL sequences based on two criteria: i) sequences with ≥ 80% coverage of the 18S rRNA region, and ii) ≥ 5% difference with previously known sequences. Our phylogenetic analysis has identified NSW332 and VIC93 as a putative novel RL; however, the sequences generated here are shorter than 80% (48%) of the full *Entamoeba* 18S rRNA gene. Therefore, future studies to determine the complete 18S rRNA gene sequence of NSW332 and VIC93 are needed to confirm this finding.

In conclusion, here we present evidence of three *Entamoeba* RLs in Australian ruminants: *E. bovis* in wild deer and cattle, *Entamoeba* RL1 in wild sambar deer, and *Entamoeba* RL8 in wild fallow deer. Our study represents the first identification of *Entamoeba* parasites in Australian deer, expanding the host range of *Entamoeba* parasites. Further, we present evidence of a potential novel *Entamoeba* species (VIC91) of wild deer origin, closely related to *Entamoeba* RL1. We detected a high prevalence of *E. bovis* (100%) in cattle in the absence of clinical signs, which aligns with the low pathogenicity of *E. bovis* and its alleged commensal relationship with its cattle host (Ai et al., 2021). Finally, our study suggests a lack of current *E. bovis* transmission between wild deer and cattle in Australia. However, considering the ongoing expansion of wild deer populations in Australia, both in size and distribution, this scenario is likely to change in the future.

## Data Availability Statement

The datasets presented in this study can be found in online repositories. The names of the repository/repositories and accession number(s) can be found in the article/**Supplementary Material**.

## Ethics Statement

The work presented in this manuscript required no specific ethical approval. Deer culling was carried out as management operations and independently from this research. Similarly, cattle samples were collected as part of clinical investigations independently from this research. In both cases, we accessed the samples opportunistically.

## Author Contributions

Conceptualisation, CP and TC. Methodology, JH, CP, and TC. Formal analysis, LK-E and JH. Investigation, JH, CP, and TC. Funding: CP, KH, and TC. Resources, CP, MD and TC. Data curation, JH. Writing—original draft preparation, JH and TC. Writing—review and editing, CP, MD, KH and TC. All authors have read and agreed to the published version of the manuscript.

## Funding

This study was funded by the Centre for Invasive Species Solutions (Grant PO1-L-002).

## Conflict of Interest

The authors declare that the research was conducted in the absence of any commercial or financial relationships that could be construed as a potential conflict of interest.

## Publisher’s Note

All claims expressed in this article are solely those of the authors and do not necessarily represent those of their affiliated organizations, or those of the publisher, the editors and the reviewers. Any product that may be evaluated in this article, or claim that may be made by its manufacturer, is not guaranteed or endorsed by the publisher.

## Supplementary Material

Supplementary Figure 1 Alignment of partial 18S rRNA DNA sequences obtained from sample NSW304 and three *Entamoeba* sp. RL8 reference strains (KR025406, MN749974, MN749989). Numbers indicate nucleotide positions. ‘*’ denotes an identical nucleotide residue across all four sequences. ‘-’ indicates the absence of a nucleotide residue. Gap positions are highlighted in green.

Supplementary Figure 2 Phylogenetic analysis of *Entamoeba* partial 18S rRNA DNA sequences. Deer sequences are highlighted in blue and cattle sequences in red. Reference sequences are indicated in black. The tree was constructed using the maximum likelihood method and Tamura 3-parameter + G substitution model. Only bootstrap values >70% are shown at the nodes. The scale bar indicates nucleotide substitutions per site.

Supplementary Figure 3 Alignment of 18S rRNA sequences included in our phylogenetic and bayesian analysis. Numbers indicate nucleotide positions. ‘*’ denotes an identical nucleotide across the alignment. ‘-’ indicates the absence of a nucleotide residue.

Supplementary Figure 4 PCR amplification of the six STRs assessed in this study [based on (21)]. **(A)** Amplification of STRs D-A5/D-A3 (400/500 bp), STGA-D5/STGA-D3 (150/200 bp), A-L5/A-L3 (500/600 bp), R-R5/R-R3 (700 bp), N-K5/N-K3 (600/800 bp), and SQ5/SQ3 (400/500 bp) was attempted in four deer samples (N301, N304, N306, N308). **(B)** Amplification of STRs N-K5/N-K3 (600/800 bp), A-L5/A-L3 (500/600 bp), SQ5/SQ3 (400/500 bp), STGA-D5/STGA-D3 (150/200 bp) was attempted in six cattle samples (LN2, LN4, LN6, LV1, LV4, 300).

Supplementary Table 1 List of 18S rRNA *Entamoeba* sequences included on the phylogenetic and divergence time analysis.

Supplementary Table 2 List of accession number of the *Entamoeba* 18S rRNA DNA sequences obtained in the present study and submitted to GenBank.
