# Supplementary figures and images for: First Evidence of Entamoeba Parasites in Australian Wild Deer and Assessment of Transmission to Cattle
Source: Front Cell Infect Microbiol. 2022 Jun 10;12:883031. doi: 10.3389/fcimb.2022.883031 (PMC9226911; doi:10.3389/fcimb.2022.883031)

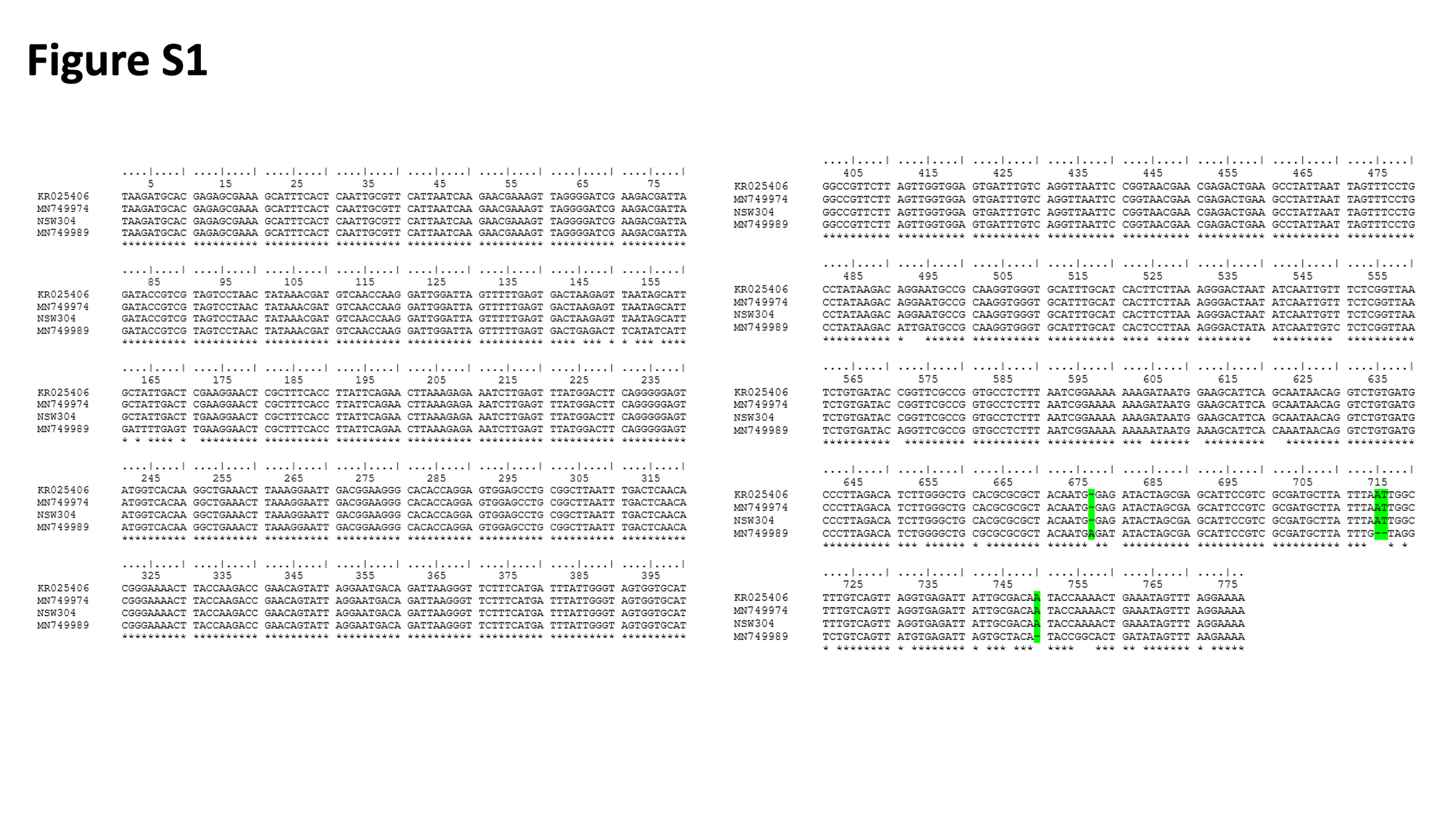

Supplement: Supplementary Figure 1 — Alignment of partial 18S rRNA DNA sequences obtained from sample NSW304 and three Entamoeba sp. RL8 reference strains (KR025406, MN749974, MN749989). Numbers indicate nucleotide positions. ‘*’ denotes an identical nucleotide residue across all four sequences. ‘-’ indicates the absence of a nucleotide residue. Gap positions are highlighted in green. [file Image_1.tiff]

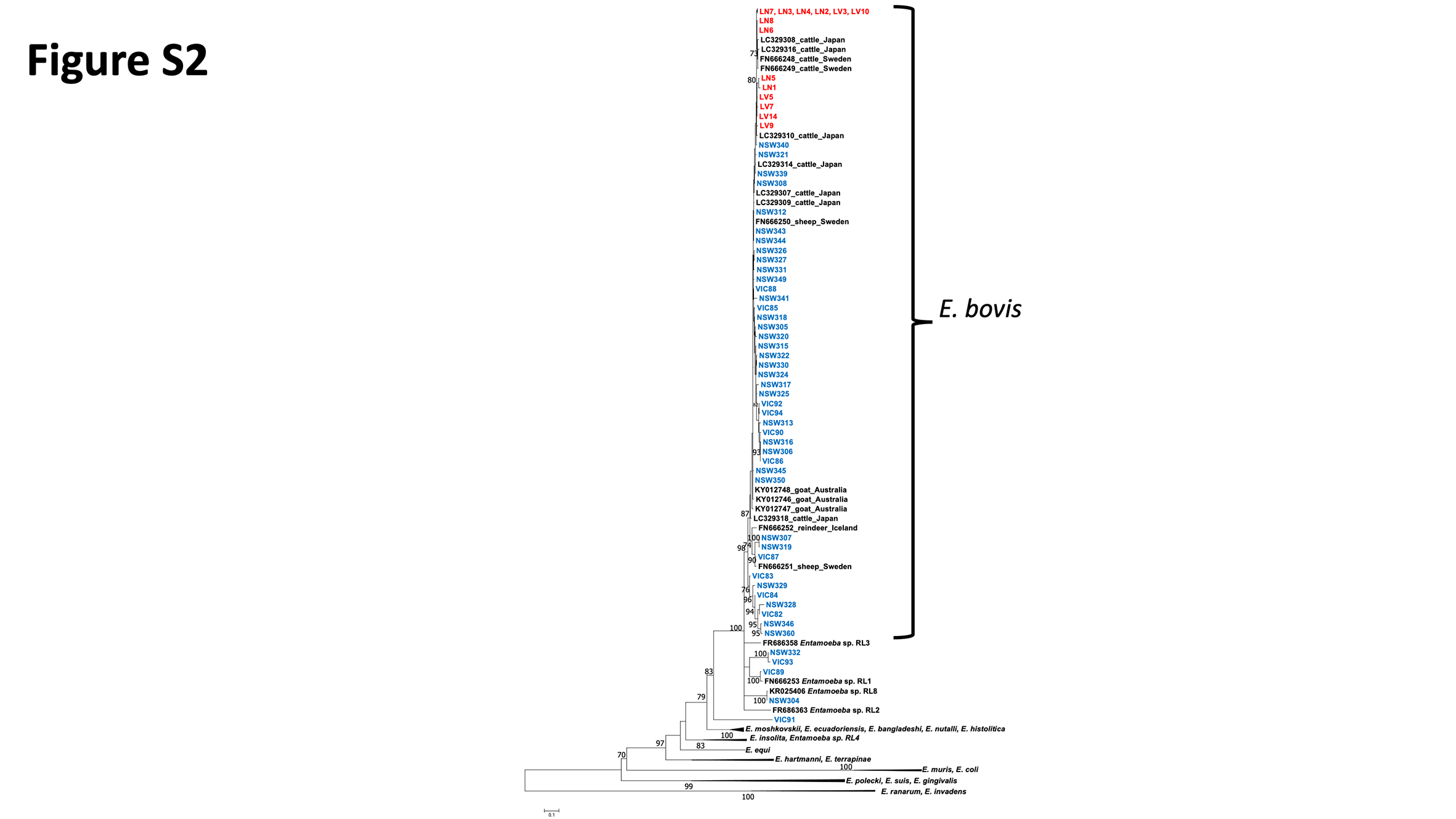

Supplement: Supplementary Figure 2 — Phylogenetic analysis of Entamoeba partial 18S rRNA DNA sequences. Deer sequences are highlighted in blue and cattle sequences in red. Reference sequences are indicated in black. The tree was constructed using the maximum likelihood method and Tamura 3-parameter + G substitution model. Only bootstrap values >70% are shown at the nodes. The scale bar indicates nucleotide substitutions per site. [file Image_2.tiff]

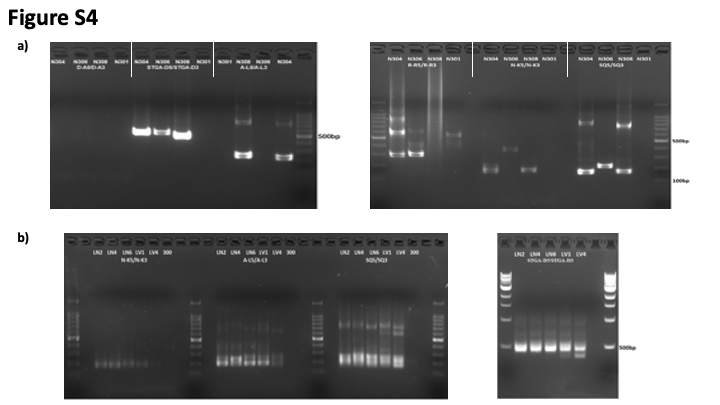

Supplement: Supplementary Figure 4 — PCR amplification of the six STRs assessed in this study [based on (21)]. (A) Amplification of STRs D-A5/D-A3 (400/500 bp), STGA-D5/STGA-D3 (150/200 bp), A-L5/A-L3 (500/600 bp), R-R5/R-R3 (700 bp), N-K5/N-K3 (600/800 bp), and SQ5/SQ3 (400/500 bp) was attempted in four deer samples (N301, N304, N306, N308). (B) Amplification of STRs N-K5/N-K3 (600/800 bp), A-L5/A-L3 (500/600 bp), SQ5/SQ3 (400/500 bp), STGA-D5/STGA-D3 (150/200 bp) was attempted in six cattle samples (LN2, LN4, LN6, LV1, LV4, 300). [file Image_4.tiff]
