# Supplementary material for: First Evidence of Entamoeba Parasites in Australian Wild Deer and Assessment of Transmission to Cattle
Source: Front Cell Infect Microbiol. 2022 Jun 10;12:883031. doi: 10.3389/fcimb.2022.883031 (PMC9226911; doi:10.3389/fcimb.2022.883031)
Supplement: Supplementary Figure 3 — Alignment of 18S rRNA sequences included in our phylogenetic and bayesian analysis. Numbers indicate nucleotide positions. ‘*’ denotes an identical nucleotide across the alignment. ‘-’ indicates the absence of a nucleotide residue. [file Image_3.pdf]

Figure S3

```
             ....|....| ....|....| ....|....| ....|....| ....|....| ....|....| ....|....| ....|....| ....|....| ....|....| ....|....| ....|....| ....|....| ....|....|
                     5         15         25         35         45         55         65         75         85         95        105        115        125        135
64142        TAAGATGCAC GAGAGCGAAA GCATTTCACT CA-ACTGTGT CCATTAATCA AGAACGAAAG TTAGGGGATC GAAGACGATC AGATACCGTC GTAGTCCTAA CTATAAACGA TGTCAACCAA GGATTGG--- ---ATGAAAT
R686356      TAAGATGCAC GAGAGCGAAA GCATTTCACT CA-ACTGTGT CCATTAATCA AGAACGAAAG TTAGGGGATC GAAGACGATC AGATACCGTC GTAGTCCTAA CTATAAACGA TGTCAACCAA GGATTGG--- ---ATGAAAT
R025411      TAAGATGCAC GAGAGCGAAA GCATTTCACT CA-ACTGGGT TCATTAATCA AGAACGAAAG TTAGGGGATC GAAGACGATC AGATACCGTC GTAGTCCTAA CTATAAACGA TGTCAACCAA GGATTGG--- ---ATGAAAT
Q286373      TAAGATGCAC GAGAGCGAAA GCATTTCACT CA-ACTGGGT TCATTAATCA AGAACGAAAG TTAGGGGATC GAAGACGATC AGATACCGTC GTAGTCCTAA CTATAAACGA TGTCAACCAA GGATTGG--- ---ATGAAAT
F149906      TAAGATGCAC GAGAGCGAAA GCATTTCACT CA-ACTGGGT CCATTAATCA AGAACGAAAG TTAGGGGATC GAAGACGATC AGATACCGTC GTAGTCCTAA CTATAAACGA TGTCAACCAA GGATTGG--- ---ATGAAAT
SW344        TAAGATGCAC GAGAGCGAAA GCATTTCACT CA-ATTGCGT TCATTAATCA AGAACGAAAG TTAGGGGATC GAAGACGATT AGATACCGTC GTAGTCCTAA CTATAAACGA TGTCAACCAA GGATTGG--- ---ATTAGTT
SW343        TAAGATGCAC GAGAGCGAAA GCATTTCACT CA-ATTGCGT TCATTAATCA AGAACGAAAG TTAGGGGATC GAAGACGATT AGATACCGTC GTAGTCCTAA CTATAAACGA TGTCAACCAA GGATTGG--- ---ATTAGTT
N666250      TAAGATGCAC GAGAGCGAAA GCATTTCACT CA-ATTGCGT TCATTAATCA AGAACGAAAG TTAGGGGATC GAAGACGATT AGATACCGTC GTAGTCCTAA CTATAAACGA TGTCAACCAA GGATTGG--- ---ATTAGTT
C329307      TAAGATGCAC GAGAGCGAAA GCATTTCACT CA-ATTGCGT TCATTAATCA AGAACGAAAG TTAGGGGATC GAAGACGATT AGATACCGTC GTAGTCCTAA CTATAAACGA TGTCAACCAA GGATTGG--- ---ATTAGTT
SW312        TAAGATGCAC GAGAGCGAAA GCATTTCACT CA-ATTGCGT TCATTAATCA AGAACGAAAG TTAGGGGATC GAAGACGATT AGATACCGTC GTAGTCCTAA CTATAAACGA TGTCAACCAA GGATTGG--- ---ATTAGTT
C329309      TAAGATGCAC GAGAGCGAAA GCATTTCACT CA-ATTGCGT TCATTAATCA AGAACGAAAG TTAGGGGATC GAAGACGATT AGATACCGTC GTAGTCCTAA CTATAAACGA TGTCAACCAA GGATTGG--- ---ATTAGTT
SW327        TAAGATGCAC GAGAGCGAAA GCATTTCACT CA-ATTGCGT TCATTAATCA AGAACGAAAG TTAGGGGATC GAAGACGATT AGATACCGTC GTAGTCCTAA CTATAAACGA TGTCAACCAA GGATTGG--- ---ATTAGTT
SW326        TAAGATGCAC GAGAGCGAAA GCATTTCACT CA-ATTGCGT TCATTAATCA AGAACGAAAG TTAGGGGATC GAAGACGATT AGATACCGTC GTAGTCCTAA CTATAAACGA TGTCAACCAA GGATTGG--- ---ATTAGTT
SW331        TAAGATGCAC GAGAGCGAAA GCATTTCACT CA-ATTGCGT TCATTAATCA AGAACGAAAG TTAGGGGATC GAAGACGATT AGATACCGTC GTAGTCCTAA CTATAAACGA TGTCAACCAA GGATTGG--- ---ATTAGTT
SW349        TAAGATGCAC GAGAGCGAAA GCATTTCACT CA-ATTGCGT TCATTAATCA AGAACGAAAG TTAGGGGATC GAAGACGATT AGATACCGTC GTAGTCCTAA CTATAAACGA TGTCAACCAA GGATTGG--- ---ATTAGTT
IC88         TAAGATGCAC GAGAGCGAAA GCATTTCACT CA-ATTGCGT TCATTAATCA AGAACGAAAG TTAGGGGATC GAAGACGATT AGATACCGTC GTAGTCCTAA CTATAAACGA TGTCAACCAA GGATTGG--- ---ATTAGTT
SW350        TAAGATGCAC GAGAGCGAAA GCATTTCACT CA-ATTGCGT TCATTAATCA AGAACGAAAG TTAGGGGATC GAAGACGATT AGATACCGTC GTAGTCCTAA CTATAAACGA TGTCAACCAA GGATTGG--- ---ATTAGTT
SW345        TAAGATGCAC GAGAGCGAAA GCATTTCACT CA-ATTGCGT TCATTAATCA AGAACGAAAG TTAGGGGATC GAAGACGATT AGATACCGTC GTAGTCCTAA CTATAAACGA TGTCAACCAA GGATTGG--- ---ATTAGTT
Y012747      TAAGATGCAC GAGAGCGAAA GCATTTCACT CA-ATTGCGT TCATTAATCA AGAACGAAAG TTAGGGGATC GAAGACGATT AGATACCGTC GTAGTCCTAA CTATAAACGA TGTCAACCAA GGATTGG--- ---ATTAGTT
Y012746      TAAGATGCAC GAGAGCGAAA GCATTTCACT CA-ATTGCGT TCATTAATCA AGAACGAAAG TTAGGGGATC GAAGACGATT AGATACCGTC GTAGTCCTAA CTATAAACGA TGTCAACCAA GGATTGG--- ---ATTAGTT
Y012748      TAAGATGCAC GAGAGCGAAA GCATTTCACT CA-ATTGCGT TCATTAATCA AGAACGAAAG TTAGGGGATC GAAGACGATT AGATACCGTC GTAGTCCTAA CTATAAACGA TGTCAACCAA GGATTGG--- ---ATTAGTT
N4           TAAGATGCAC GAGAGCGAAA GCATTTCACT CA-ATTGCGT TCATTAATCA AGAACGAAAG TTAGGGGATC GAAGACGATT AGATACCGTC GTAGTCCTAA CTATAAACGA TGTCAACCAA GGATTGG--- ---ATTAGTT
N3           TAAGATGCAC GAGAGCGAAA GCATTTCACT CA-ATTGCGT TCATTAATCA AGAACGAAAG TTAGGGGATC GAAGACGATT AGATACCGTC GTAGTCCTAA CTATAAACGA TGTCAACCAA GGATTGG--- ---ATTAGTT
N7           TAAGATGCAC GAGAGCGAAA GCATTTCACT CA-ATTGCGT TCATTAATCA AGAACGAAAG TTAGGGGATC GAAGACGATT AGATACCGTC GTAGTCCTAA CTATAAACGA TGTCAACCAA GGATTGG--- ---ATTAGTT
N2           TAAGATGCAC GAGAGCGAAA GCATTTCACT CA-ATTGCGT TCATTAATCA AGAACGAAAG TTAGGGGATC GAAGACGATC AGATACCGTC GTAGTCCTAA CTATAAACGA TGTCAACCAA GGATTGG--- ---ATTAGTT
V10          TAAGATGCAC GAGAGCGAAA GCATTTCACT CA-ATTGCGT TCATTAATCA AGAACGAAAG TTAGGGGATC GAAGACGATT AGATACCGTC GTAGTCCTAA CTATAAACGA TGTCAACCAA GGATTGG--- ---ATTAGTT
V3           TAAGATGCAC GAGAGCGAAA GCATTTCACT CA-ATTGCGT TCATTAATCA AGAACGAAAG TTAGGGGATC GAAGACGATT AGATACCGTC GTAGTCCTAA CTATAAACGA TGTCAACCAA GGATTGG--- ---ATTAGTT
N8           TAAGATGCAC GAGAGCGAAA GCATTTCACT CA-ATTGCGT TCATTAATCA AGAACGAAAG TTAGGGGATC GAAGACGATT AGATACCGTC GTAGTCCTAA CTATAAACGA TGTCAACCAA GGATTGG--- ---ATTAGTT
N666249      TAAGATGCAC GAGAGCGAAA GCATTTCACT CA-ATTGCGT TCATTAATCA AGAACGAAAG TTAGGGGATC GAAGACGATT AGATACCGTC GTAGTCCTAA CTATAAACGA TGTCAACCAA GGATTGG--- ---ATTAGTT
N666248      TAAGATGCAC GAGAGCGAAA GCATTTCACT CA-ATTGCGT TCATTAATCA AGAACGAAAG TTAGGGGATC GAAGACGATT AGATACCGTC GTAGTCCTAA CTATAAACGA TGTCAACCAA GGATTGG--- ---ATTAGTT
C329316      TAAGATGCAC GAGAGCGAAA GCATTTCACT CA-ATTGCGT TCATTAATCA AGAACGAAAG TTAGGGGATC GAAGACGATT AGATACCGTC GTAGTCCTAA CTATAAACGA TGTCAACCAA GGATTGG--- ---ATTAGTT
C329308      TAAGATGCAC GAGAGCGAAA GCATTTCACT CA-ATTGCGT TCATTAATCA AGAACGAAAG TTAGGGGATC GAAGACGATT AGATACCGTC GTAGTCCTAA CTATAAACGA TGTCAACCAA GGATTGG--- ---ATTAGTT
V14          TAAGATGCAC GAGAGCGAAA GCATTTCACT CA-ATTGCGT TCATTAATCA AGAACGAAAG TTAGGGGATC GAAGACGATT AGATACCGTC GTAGTCCTAA CTATAAACGA TGTCAACCAA GGATTGG--- ---ATTAGTT
V7           TAAGATGCAC GAGAGCGAAA GCATTTCACT CA-ATTGCGT TCATTAATCA AGAACGAAAG TTAGGGGATC GAAGACGATT AGATACCGTC GTAGTCCTAA CTATAAACGA TGTCAACCAA GGATTGG--- ---ATTAGTT
V5           TAAGATGCAC GAGAGCGAAA GCATTTCACT CA-ATTGCGT TCATTAATCA AGAACGAAAG TTAGGGGATC GAAGACGATT AGATACCGTC GTAGTCCTAA CTATAAACGA TGTCAACCAA GGATTGG--- ---ATTAGTT
N6           TAAGATGCAC GAGAGCGAAA GCATTTCACT CA-ATTGCGT TCATTAATCA AGAACGAAAG TTAGGGGATC GAAGACGATT AGATACCGTC GTAGTCCTAA CTATAAACGA TGTCAACCAA GGATTGG--- ---ATTAGTT
N1           TAAGATGCAC GAGAGCGAAA GCATTTCACT CA-ATTGCGT TCATTAATCA AGAACGAAAG TTAGGGGATC GAAGACGATC AGATACCGTC GTAGTCCTAA CTATAAACGA TGTCAACCAA GGATTGG--- ---ATTAGTT
N5           TAAGATGCAC GAGAGCGAAA GCATTTCACT CA-ATTGCGT TCATTAATCA AGAACGAAAG TTAGGGGATC GAAGACGATC AGATACCGTC GTAGTCCTAA CTATAAACGA TGTCAACCAA GGATTGG--- ---ATTAGTT
V9           TAAGATGCAC GAGAGCGAAA GCATTTCACT CA-ATTGCGT TCATTAATCA AGAACGAAAG TTAGGGGATC GAAGACGATT AGATACCGTC GTAGTCCTAA CTATAAACGA TGTCAACCAA GGATTGG--- ---ATTAGTT
C329310      TAAGATGCAC GAGAGCGAAA GCATTTCACT CA-ATTGCGT TCATTAATCA AGAACGAAAG TTAGGGGATC GAAGACGATT AGATACCGTC GTAGTCCTAA CTATAAACGA TGTCAACCAA GGATTGG--- ---ATTAGTT
SW340        TAAGATGCAC GAGAGCGAAA GCATTTCACT CA-ATTGCGT TCATTAATCA AGAACGAAAG TTAGGGGATC GAAGACGATT AGATACCGTC GTAGTCCTAA CTATAAACGA TGTCAACCAA GGATTGG--- ---ATTAGTT
SW339        TAAGATGCAC GAGAGCGAAA GCATTTCACT CA-ATTGCGT TCATTAATCA AGAACGAAAG TTAGGGGATC GAAGACGATT AGATACCGTC GTAGTCCTAA CTATAAACGA TGTCAACCAA GGATTGG--- ---ATTAGTT
C329314      TAAGATGCAC GAGAGCGAAA GCATTTCACT CA-ATTGCGT TCATTAATCA AGAACGAAAG TTAGGGGATC GAAGACGATT AGATACCGTC GTAGTCCTAA CTATAAACGA TGTCAACCAA GGATTGG--- ---ATTAGTT
SW308        TAAGATGCAC GAGAGCGAAA GCATTTCACT CA-ATTGCGT TCATTAATCA AGAACGAAAG TTAGGGGATC GAAGACGATT AGATACCGTC GTAGTCCTAA CTATAAACGA TGTCAACCAA GGATTGG--- ---ATTAGTT
IC85         TAAGATGCAC GAGAGCGAAA GCATTTCACT CA-ATTGCGT TCATTAATCA AGAACGAAAG TTAGGGGATC GAAGACGATC AGATACCGTC GTAGTCCTAA CTATAAACGA TGTCAACCAA GGATTGG--- ---ATTAGTT
SW318        TAAGATGCAC GAGAGCGAAA GCATTTCACT CA-ATTGCGT TCATTAATCA AGAACGAAAG TTAGGGGATC GAAGACGATC AGATACCGTC GTAGTCCTAA CTATAAACGA TGTCAACCAA GGATTGG--- ---ATTAGTT
SW330        TAAGATGCAC GAGAGCGAAA GCATTTCACT CA-ATTGCGT TCATTAATCA AGAACGAAAG TTAGGGGATC GAAGACGATC AGATACCGTC GTAGTCCTAA CTATAAACGA TGTCAACCAA GGATTGG--- ---ATTAGTT
SW322        TAAGATGCAC GAGAGCGAAA GCATTTCACT CA-ATTGCGT TCATTAATCA AGAACGAAAG TTAGGGGATC GAAGACGATT AGATACCGTC GTAGTCCTAA CTATAAACGA TGTCAACCAA GGATTGG--- ---ATTAGTT
SW324        TAAGATGCAC GAGAGCGAAA GCATTTCACT CA-ATTGCGT TCATTAATCA AGAACGAAAG TTAGGGGATC GAAGACGATC AGATACCGTC GTAGTCCTAA CTATAAACGA TGTCAACCAA GGATTGG--- ---ATTAGTT
SW305        TAAGATGCAC GAGAGCGAAA GCATTTCACT CA-ATTGCGT TCATTAATCA AGAACGAAAG TTAGGGGATC GAAGACGATC AGATACCGTC GTAGTCCTAA CTATAAACGA TGTCAACCAA GGATTGG--- ---ATTAGTT
SW315        TAAGATGCAC GAGAGCGAAA GCATTTCACT CA-ATTGCGT TCATTAATCA AGAACGAAAG TTAGGGGATC GAAGACGATC AGATACCGTC GTAGTCCTAA CTATAAACGA TGTCAACCAA GGATTGG--- ---ATTAGTT
SW320        TAAGATGCAC GAGAGCGAAA GCATTTCACT CA-ATTGCGT TCATTAATCA AGAACGAAAG TTAGGGGATC GAAGACGATC AGATACCGTC GTAGTCCTAA CTATAAACGA TGTCAACCAA GGATTGG--- ---ATTAGTT
SW321        TAAGATGCAC GAGAGCGAAA GCATTTCACT CA-ATTGCGT TCATTAATCA AGAACGAAAG TTAGGGGATC GAAGACGATC AGATACCGTC GTAGTCCTAA CTATAAACGA TGTCAACCAA GGATTGG--- ---ATTAGTT
SW341        TAAGATGCAC GAGAGCGAAA GCATTTCACT CA-ATTGCGT TCATTAATCA AGAACGAAAG TTAGGGGATC GAAGACGATC AGATACCGTC GTAGTCCTAA CTATAAACGA TGTCAACCAA GGATTGG--- ---ATTAGTT
IC94         TAAGATGCAC GAGAGCGAAA GCATTTCACT CA-ATTGCGT TCATTAATCA AGAACGAAAG TTAGGGGATC GAAGACGATT AGATACCGTC GTAGTCCTAA CTATAAACGA TGTCAACCAA GGATTGG--- ---ATTAGTT
IC92         TAAGATGCAC GAGAGCGAAA GCATTTCACT CA-ATTGCGT TCATTAATCA AGAACGAAAG TTAGGGGATC GAAGACGATT AGATACCGTC GTAGTCCTAA CTATAAACGA TGTCAACCAA GGATTGG--- ---ATTAGTT
IC86         TAAGATGCAC GAGAGCGAAA GCATTTCACT CA-ATTGCGT TCATTAATCA AGAACGAAAG TTAGGGGATC GAAGACGATT AGATACCGTC GTAGTCCTAA CTATAAACGA TGTCAACCAA GGATTGG--- ---ATTAGTT
```

```
SW316           TAAGATGCAC GAGAGCGAAA GCATTTCACT CA-ATTGCGT TCATTAATCA AGAACGAAAG TTAGGGGATC GAAGACGATC AGATACCGTC GTAGTCCTAA CTATAAACGA TGTCAACCAA GGATTGG--- ---ATTAGTT
IC90            TAAGATGCAC GAGAGCGAAA GCATTTCACT CA-ATTGCGT TCATTAATCA AGAACGAAAG TTAGGGGATC GAAGACGATT AGATACCGTC GTAGTCCTAA CTATAAACGA TGTCAACCAA GGATTGG--- ---ATTAGTT
SW313           TAAGATGCAC GAGAGCGAAA GCATTTCACT CA-ATTGCGT TCATTAATCA AGAACGAAAG TTAGGGGATC GAAGACGATT AGATACCGTC GTAGTCCTAA CTATAAACGA TGTCAACCAA GGATTGG--- ---ATTAGTT
SW317           TAAGATGCAC GAGAGCGAAA GCATTTCACT CA-ATTGCGT TCATTAATCA AGAACGAAAG TTAGGGGATC GAAGACGATC AGATACCGTC GTAGTCCTAA CTATAAACGA TGTCAACCAA GGATTGG--- ---ATTAGTT
                ....|....| ....|....| ....|....| ....|....| ....|....| ....|....| ....|....| ....|....| ....|....| ....|....| ....|....| ....|....| ....|....| ....|....|
                    5          15         25         35         45         55         65         75         85         95         105        115        125        135
SW325           TAAGATGCAC GAGAGCGAAA GCATTTCACT CA-ATTGCGT TCATTAATCA AGAACGAAAG TTAGGGGATC GAAGACGATC AGATACCGTC GTAGTCCTAA CTATAAACGA TGTCAACCAA GGATTGG--- ---ATTAGTT
C329318         TAAGATGCAC GAGAGCGAAA GCATTTCACT CA-ATTGCGT TCATTAATCA AGAACGAAAG TTAGGGGATC GAAGACGATT AGATACCGTC GTAGTCCTAA CTATAAACGA TGTCAACCAA GGATTGG--- ---ATTAGTT
N666251         TAAGATGCAC GAGAGCGAAA GCATTTCACT CA-ATTGCGT TCATTAATCA AGAACGAAAG TTAGGGGATC GAAGACGATT AGATACCGTC GTAGTCCTAA CTATAAACGA TGTCAACCAA GGATTGG--- ---ATTAGTT
IC87            TAAGATGCAC GAGAGCGAAA GCATTTCACT CA-ATTGCGT TCATTAATCA AGAACGAAAG TTAGGGGATC GAAGACGATT AGATACCGTC GTAGTCCTAA CTATAAACGA TGTCAACCAA GGATTGG--- ---ATTAGTT
SW319           TAAGATGCAC GAGAGCGAAA GCATTTCACT CA-ATTGCGT TCATTAATCA AGAACGAAAG TTAGGGGATC GAAGACGATT AGATACCGTC GTAGTCCTAA CTATAAACGA TGTCAACCAA GGATTGG--- ---ATTAGTT
SW307           TAAGATGCAC GAGAGCGAAA GCATTTCACT CA-ATTGCGT TCATTAATCA AGAACGAAAG TTAGGGGATC GAAGACGATT AGATACCGTC GTAGTCCTAA CTATAAACGA TGTCAACCAA GGATTGG--- ---ATTAGTT
N666252         TAAGATGCAC GAGAGCGAAA GCATTTCACT CA-ATTGCGT TCATTAATCA AGAACGAAAG TTAGGGGATC GAAGACGATT AGATACCGTC GTAGTCCTAA CTATAAACGA TGTCAACCAA GGATTGG--- ---ATTAGTT
SW360           TAAGATGCAC GAGAGCGAAA GCATTTCACT CA-ATTGCGT TCATTAATCA AGAACGAAAG TTAGGGGATC GAAGACGATC AGATACCGTC GTAGTCCTAA CTATAAACGA TGTCAACCAA GGATTGG--- ---ATTAGTT
SW346           TAAGATGCAC GAGAGCGAAA GCATTTCACT CA-ATTGCGT TCATTAATCA AGAACGAAAG TTAGGGGATC GAAGACGATC AGATACCGTC GTAGTCCTAA CTATAAACGA TGTCAACCAA GGATTGG--- ---ATTAGTT
IC82            TAAGATGCAC GAGAGCGAAA GCATTTCACT CA-ATTGCGT TCATTAATCA AGAACGAAAG TTAGGGGATC GAAGACGATC AGATACCGTC GTAGTCCTAA CTATAAACGA TGTCAACCAA GGATTGG--- ---ATTAGTT
SW328           TAAGATGCAC GAGAGCGAAA GCATTTCACT CA-ATTGCGT TCATTAATCA AGAACGAAAG TTAGGGGATC GAAGACGATC AGATACCGTC GTAGTCCTAA CTATAAACGA TGTCAACCAA GGATTGG--- ---ATTAGTT
IC84            TAAGATGCAC GAGAGCGAAA GCATTTCACT CA-ATTGCGT TCATTAATCA AGAACGAAAG TTAGGGGATC GAAGACGATC AGATACCGTC GTAGTCCTAA CTATAAACGA TGTCAACCAA GGATTGG--- ---ATTAGTT
SW329           TAAGATGCAC GAGAGCGAAA GCATTTCACT CA-ATTGCGT TCATTAATCA AGAACGAAAG TTAGGGGATC GAAGACGATC AGATACCGTC GTAGTCCTAA CTATAAACGA TGTCAACCAA GGATTGG--- ---ATTAGTT
IC83            TAAGATGCAC GAGAGCGAAA GCATTTCACT CA-ATTGCGT TCATTAATCA AGAACGAAAG TTAGGGGATC GAAGACGATC AGATACCGTC GTAGTCCTAA CTATAAACGA TGTCAACCAA GGATTGG--- ---ATTAGTT
SW304           TAAGATGCAC GAGAGCGAAA GCATTTCACT CA-ATTGCGT TCATTAATCA AGAACGAAAG TTAGGGGATC GAAGACGATT AGATACCGTC GTAGTCCTAA CTATAAACGA TGTCAACCAA GGATTGG--- ---ATTAGTT
R025406         TAAGATGCAC GAGAGCGAAA GCATTTCACT CA-ATTGCGT TCATTAATCA AGAACGAAAG TTAGGGGATC GAAGACGATT AGATACCGTC GTAGTCCTAA CTATAAACGA TGTCAACCAA GGATTGG--- ---ATTAGTT
R686358         TAAGATGCAC GAGAGCGAAA GCATTTCACT CA-ATTGCGT TCATTAATCA AGAACGAAAG TTAGGGGATC GAAGACGATC AGATACCGTC GTAGTCCTAA CTATAAACGA TGTCAACCAA GGATTGGG-- ---ATTAGTT
N666253         TAGGATGCAC GAGAGCGAAA GCATTTCACT CA-ATTGCGT TCATTAATCA AGAACGAAAG TTAGGGGATC GAAGACGATT AGATACCGTC GTAGTCCTAA CTATAAACGA TGTCAACCAA GGATTGG--- ---ATTAGTA
IC89            TAGGATGCAC GAGAGCGAAA GCATTTCACT CA-ATTGCGT TCATTAATCA AGAACGAAAG TTAGGGGATC GAAGACGATT AGATACCGTC GTAGTCCTAA CTATAAACGA TGTCAACCAA GGATTGG--- ---ATTAGTA
R686363         TAAGATGCAC GAGAGCGAAA GCATTTCACT CA-ATTGCGT TCATTAATCA AGAACGAAAG TTAGGGGATC GAAGACGATC AGATACCGTC GTAGTCCTAA CTATAAACGA TGTCAACCAA GGATTGG--- ---ATTAGTC
IC93            TAAGATGCAC GAGAGCGAAA GCATTTCACT CA-ATTGCGT TCATTAATCA AGAACGAAAG TTAGGGGATC GAAGACGATC AGATACCGTC GTAGTCCTAA CTATAAACGA TGTCAACCAA GGATTGG--- ---ATTAGTC
SW332           TAAGATGCAC GAGAGCGAAA GCATTTCACT CA-ATTGCGT TCATTAATCA AGAACGAAAG TTAGGGGATC GAAGACGATC AGATACCGTC GTAGTCCTAA CTATAAACGA TGTCAACCAA GGATTGG--- ---ATTAGTC
R686361         TAAGATGAAC GAGAGCGAAA GCATTTCACT CA-ATTATGT CCATTAATCA AGAACGAAAG TTAGGGGATC GAAGACGATC AGATACCGTC GTAGTCCTAA CTATAAACGA TGTCAACCAA GGATTGG--- ---ATAAATT
F149909         TAAGATGAAC TAGAGCGAAA GCATTTCACT CA-ATTATAT TCATTAATCA AGAACGAAAG TTAGGGGATC GAAGACGATC AGATACCGTC GTAGTCCTAA CTATAAACGA TGTCAACCAA GGATTGG--- ---ATAAATT
IC91            TAAGATGCAC GAGAGCGAAA GCATTTCACT CA-ATTGCGT TCATTAATCA AGAACGAAAG TTAGGGGATC GAAGACGATT AGATACCGTC GTAGTCCTAA CTATAAACGA TGTCAACCAA GGATTGG--- ---ATTCGCT
Q286371         TAAGATGATC GAGAGCGAAA GCATTTCACT CA-TTTATCT TCATTAATCA AGAACGAAAG TTAGGGGATC GAAGACGATC AGATACCGTC GTAGTCCTAA CTATWAACGA TGTCAACCAA GGATTGG--- ---ATAAGCT
F149915         CGAGATAAAC GAGAGCGAAA GCATTTCACT GA-ATTCATT TCATTAATCA AGAACGAAAG TCAGGGAATC AAAGACGATT AGATACCGTC GTAGTCCTGA CCATAAACGA TGTCAACTAA GGATTGGAT- --AAGAAACA
B445018         CGAGATAAGC GAGAGCGAAA GCATTTCACT GA-ACTCATT TCATTAATCA AGAACGAAAG TCAGGGAATC GAAGACGATT AGATACCGTC GTAGTCCTGA CCATAAACGA TGTCAACTAA GGATTGGATT GGAACAAACA
F149910         TAAGATGCAC GAGAGCGAAA GCATTTCACT CA-ATTATAT TCATTAATCA AGAACGAAAG TTAGGGGATC GAAGACGATC AGATACCGTC GTAGTCCTAA CTATAAACGA TGTCAACCAA GGATTAA--- ---ATGAACA
F149907         TAAGATGAAC TAGAGCGAAA GCATTTTACT CA-ATTATGT TCATTAATCA AGAACGAAAG TTAGGGGATC GAAGACGATC AGATACCGTC GTAGTCCTAA CTATAAACGA TGTCAACGAA GGATTGG--- ---ATAAATT
X027294         TTAGATAAAC GAGAGCGAAA GCATTTTACT CA-ATTATCT TCATTAATCA AGAACGAAAG TTAGGGGAAC GAAGACGATC AGATACCGTC GTAGTCCTAA CTATAAACGA TGTTAACCAA GGATTGGATA TATATTCATA
Q286372         TTAGATAAAC TAGAGCGAAA GCATTTTACT CA-ATTATAT TCATTAATCA AGAACGAAAG TTAGGGGATC GAAGACGATC AGATACCGTC GTAGTCCTAA CTATAAACGA TGTTAACCAA GGATTGGATA AAATTTAAAA
F149913         TTGGATAAAC TAAAGCGAAA GCATTTTACT CATATTCTTT TATTTGATCA AGAACGAAAG TTAGGGGATC GAAGTCGATT AGATACCGTC GTAGTCCTAA CTATAAACTA TGTCAACTGG GGATTGGATG AAAATAAACA
Y769863         TAAGATTGTC TAGAGCGAAA GCATTTTAC- CAGCACGGAT TCATTAATCA AGAACGACAG TTAGGGGATC GAAGACGATT AGATACCGTC GTAGTCCTGA CTATAAACGA TATGAACTGA AGATTATACC TGTACAGAAA
F149908         TAAGATGTCC AAAAGCGAAA GCATTTTAC- CAGGATAGAT TCATTAATCA AGAACGACAG TTAGGGGATC GAAGACGATT AGATACCGTC GTAGTCCTGA CTATAAACGA TATGGACCAA GGATTGAA-- --TAGATCAA
onsensus           ***   *  * ******* ****** **   *       *    ** **** ******* ** * **** * *  *** ****  ********** ******** * * ** *** * * *  **     ****

                ....|....| ....|....| ....|....| ....|....| ....|....| ....|....| ....|....| ....|....| ....|....| ....|....| ....|....| ....|....| ....|....| ....|....|
                    145        155        165        175        185        195        205        215        225        235        245        255        265        275
64142           TCAGATG-TA CAAAGATAGA GAAGCATT-- -GTTTCTAGA ---------T CTGAGTA--T ATCAATATTA CCTT-GTTCA GAACTTAAAG AGAAATC-TT GAGTTTATGG ACTTCAGGGG GAGTATGGTC ACAAGGCTGA
R686356         TCAGATG-TA CAAAGATGAA GAAGCATT-- -GTTTCTTAA ---------T CTGAGTA--T ATCAATATTA CCTT-GTTCA GAACTTAAAG AGAAATC-TT GAGTTTATGG ACTTCAGGGG GAGTATGGTC ACAAGGCTGA
R025411         TCAGATA-TA TTTTGATGAA AAAACATT-- -GTTTTTGAT ---------T TAAAATG--T ATCAATACTA CCTT-GTTCA GAACTTAAAG AGAAATC-TT GAGTTTATGG ACTTCAGGGG GAGTATGGTC ACAAGGCTGA
Q286373         TCAGATA-TA AAAAGATGAA GAGACATA-- -GTTTCTGAA ---------T CTGATTA--T ATCAATACTA CCTT-GTTCA GAACTTAAAG AGAAATC-TT GAGTTTATGG ACTTCAGGGG GAGTATGGTC ACAAGGCTGA
F149906         AAAGAAA-TT CGCGGATGAA GAAACATT-- -GTTTCGGAA ---------A CCAAGAG--T TTCACAACTA CCTT-GTTCA GAACTTAAAG AGAAATC-TC GAGTTTATGG ACTTCAGGGG GAGTATGGTC ACAAGGCTGA
SW344           TTAGAGT-GA CAGAAGTCCG GTAACGCT-- -GTTACTGGG ---------T TGACGGA--T CTCGCTTCCA CCTT-ATTCA GAACTTAAAG AGAAATC-TT GAGTTTATGG ACTTCAGGGG GAGTATGGTC ACAAGGCTGA
SW343           TTAGAGT-GA CAGAAGTCCG GTAACGCT-- -GTTACTGGG ---------T TGACGGA--T CTCGCTTCCA CCTT-ATTCA GAACTTAAAG AGAAATC-TT GAGTTTATGG ACTTCAGGGG GAGTATGGTC ACAAGGCTGA
N666250         TTAGAGT-GA CAGAAGTCCG GTAACGCT-- -GTTACTGGG ---------T TGACGGA--T CTCGCTTCCA CCTT-ATTCA GAACTTAAAG AGAAATC-TT GAGTTTATGG ACTTCAGGGG GAGTATGGTC ACAAGGCTGA
C329307         TTAGAGT-GA CAGAAGTCCG GTAACGCT-- -GTTACTGGG ---------T TGACGGA--T CTCGCTTCCA CCTT-ATTCA GAACTTAAAG AGAAATC-TT GAGTTTATGG ACTTCAGGGG GAGTATGGTC ACAAGGCTGA
SW312           TTAGAGT-GA CAGAAGTCCG GTAACGCT-- -GTTACTGGG ---------T TGACGGA--T CTCGCTTCCA CCTT-ATTCA GAACTTAAAG AGAAATC-TT GAGTTTATGG ACTTCAGGGG GAGTATGGTC ACAAGGCTGA
C329309         TTAGAGT-GA CAGAAGTCCG GTAACGCT-- -GTTACTGGG ---------T TGACGGA--T CTCGCTTCCA CCTT-ATTCA GAACTTAAAG AGAAATC-TT GAGTTTATGG ACTTCAGGGG GAGTATGGTC ACAAGGCTGA
SW327           TTAGAGC-GA CAGAAGTCCG GTAACGCT-- -GTTACTGGG ---------T TGACGGA--T CTCGCTTCCA CCTT-ATTCA GAACTTAAAG AGAAATC-TT GAGTTTATGG ACTTCAGGGG GAGTATGGTC ACAAGGCTGA
SW326           TTAGAGT-GA CAGAAGTCCG GTAACGCT-- -GTTACTGGG ---------T TGACGGA--T CTCGCTTCCA CCTT-ATTCA GAACTTAAAG AGAAATC-TT GAGTTTATGG ACTTCAGGGG GAGTATGGTC ACAAGGCTGA
SW331           TTAGAGT-GA CAGAAGTCCG GTAACGCT-- -GTTACTGGG ---------T TGACGGA--T CTCGCTTCCA CCTT-ATTCA GAACTTAAAG AGAAATC-TT GAGTTTATGG ACTTCAGGGG GAGTATGGTC ACAAGGCTGA
SW349           TTAGAGT-GA CAGAAGTCCG GTAACGCT-- -GTTATTGGG ---------T TGACGGA--T CTCGCTTCCA CCTT-ATTCA GAACTTAAAG AGAAATC-TT GAGTTTATGG ACTTCAGGGG GAGTATGGTC ACAAGGCTGA
IC88            TTAGAGT-GA CAGAAGTCCG GTAACGCT-- -GTTACTGGG ---------C TGACGGA--T CTCGCTTCCA CCTT-ATTCA GAACTTAAAG AGAAATC-TT GAGTTTATGG ACTTCAGGGG GAGTATGGTC ACAAGGCTGA
SW350           TTAGAGT-GA CAGAAGTCCG GTAACGCT-- -GTTACTGGG ---------T TGACGGA--T CTCGCTTCCA CCTT-ATTCA GAACTTAAAG AGAAATC-TT GAGTTTATGG ACTTCAGGGG GAGTATGGTC ACAAGGCTGA
SW345           TTAGAGT-GA CAGAAGTCCG GTAACGCT-- -GTTACTGGG ---------T TGACGGA--T CTCGCTTCCA CCTT-ATTCA GAACTTAAAG AGAAATC-TT GAGTTTATGG ACTTCAGGGG GAGTATGGTC ACAAGGCTGA
```

```
Y012746   TTAGAGT-GA CAGAAGTCCG GTAACGCT-- -GTTACTGGG ---------T TGACGGA--T CTCGCTTCCA CCTT-ATTCA GAACTTAAAG AGAAATC-TT GAGTTTATGG ACTTCAGGGG GAGTATGGTC ACAAGGCTGA
Y012748   TTAGAGT-GA CAGAAGTCCG GTAACGCT-- -GTTACTGGG ---------T TGACGGA--T CTCGCTTCCA CCTT-ATTCA GAACTTAAAG AGAAATC-TT GAGTTTATGG ACTTCAGGGG GAGTATGGTC ACAAGGCTGA
N4        TTAGAGT-GA CAGAAGTCCG GTAACACT-- -GTTACTGGG ---------C TGACGGA--T CTCGCTTCCA CCTT-ATTCA GAACTTAAAG AGAAATC-TT GAGTTTATGG ACTTCAGGGG GAGTATGGTC ACAAGGCTGA
N3        TTAGAGT-GA CAGAAGTCCG GTAACACT-- -GTTACTGGG ---------C TGACGGA--T CTCGCTTCCA CCTT-ATTCA GAACTTAAAG AGAAATC-TT GAGTTTATGG ACTTCAGGGG GAGTATGGTC ACAAGGCTGA
N7        TTAGAGT-GA CAGAAGTCCG GTAACACT-- -GTTACTGGG ---------C TGACGGA--T CTCGCTTCCA CCTT-ATTCA GAACTTAAAG AGAAATC-TT GAGTTTATGG ACTTCAGGGG GAGTATGGTC ACAAGGCTGA
N2        TTAGAGT-GA CAGAAGTCCG GTAACACT-- -GTTACTGGG ---------C TGACGGA--T CTCGCTTCCA CCTT-ATTCA GAACTTAAAG AGAAATC-TT GAGTTTATGG ACTTCAGGGG GAGTATGGTC ACAAGGCTGA
V10       TTAGAGT-GA CAGAAGTCCG GTAACACT-- -GTTACTGGG ---------C TGACGGA--T CTCGCTTCCA CCTT-ATTCA GAACTTAAAG AGAAATC-TT GAGTTTATGG ACTTCAGGGG GAGTATGGTC ACAAGGCTGA
V3        TTAGAGT-GA CAGAAGTCCG GTAACACT-- -GTTACTGGG ---------C TGACGGA--T CTCGCTTCCA CCTT-ATTCA GAACTTAAAG AGAAATC-TT GAGTTTATGG ACTTCAGGGG GAGTATGGTC ACAAGGCTGA
N8        TTAGAGT-GA CAGAAGTCCG GTAACACT-- -GTTACTGGG ---------C TGACGGA--T CTCGCTTCCA CCTT-ATTCA GAACTTAAAG AGAAATC-TT GAGTTTATGG ACTTCAGGGG GAGTATGGTC ACAAGGCTGA
N666249   TTAGAGT-GA CAGAAGTCCG GTAACACT-- -GTTACTGGG ---------C TGACGGA--T CTCGCTTCCA CCTT-ATTCA GAACTTAAAG AGAAATC-TC GAGTTTATGG ACTTCAGGGG GAGTATGGTC ACAAGGCTGA
N666248   TTAGAGT-GA CAGAAGTCCG GTAACACT-- -GTTACTGGG ---------C TGACGGA--T CTCGCTTCCA CCTT-ATTCA GAACTTAAAG AGAAATC-TC GAGTTTATGG ACTTCAGGGG GAGTATGGTC ACAAGGCTGA
C329316   TTAGAGT-GA CAGAAGTCCG GTAACACC-- -GTTGCTGGG ---------C TGACGGA--T CTCGCTTCCA CCTT-ATTCA GAACTTAAAG AGAAATC-TC GAGTTTATGG ACTTCAGGGG GAGTATGGTC ACAAGGCTGA
C329308   TTAGAGT-GA CAGAAGTCCG GTAACACT-- -GTTACTGGG ---------C TGACGGA--T CTCGCTTCCA CCTT-ATTCA GAACTTAAAG AGAAATC-TT GAGTTTATGG ACTTCAGGGG GAGTATGGTC ACAAGGCTGA
V14       TTAGAGT-GA CAGAAGTCCG GTAACACA-- -GTTACTGGG ---------C TGACGGA--T CTCGCTTCCA CCTT-ATTCA GAACTTAAAG AGAAATC-TT GAGTTTATGG ACTTCAGGGG GAGTATGGTC ACAAGGCTGA
V7        TTAGAGT-GA CAGAAGTCCG GTAACACA-- -GTTACTGGG ---------C TGACGGA--T CTCGCTTCCA CCTT-ATTCA GAACTTAAAG AGAAATC-TT GAGTTTATGG ACTTCAGGGG GAGTATGGTC ACAAGGCTGA
V5        TTAGAGT-GA CAGAAGTCCG GTAACACA-- -GTTACTGGG ---------C TGACGGA--T CTCGCTTCCA CCTT-ATTCA GAACTTAAAG AGAAATC-TT GAGTTTATGG ACTTCAGGGG GAGTATGGTC ACAAGGCTGA
N6        TTAGAGT-GA CAGAAGTCCG GTAACACT-- -GTTACTGGG ---------C TGACGGA--T CTCGCTTCCA CCTT-ATTCA GAACTTAAAG AGAAATC-TT GAGTTTATGG ACTTCAGGGG GAGTATGGTC ACAAGGCTGA
N1        TTAGAGT-GA CAGAAGTCCG ATAACACG-- -GTTATCGGG ---------C TGACGGA--T CTCGCTTCCA CCTT-ATTCA GAACTTAAAG AGAAATC-TT GAGTTTATGG ACTTCAGGGG GAGTATGGTC ACAAGGCTGA
N5        TTAGAGT-GA CAGAAGTCCG GTAACACG-- -GTTACTGGG ---------C TGACGGA--T CTCGCTTCCA CCTT-ATTCA GAACTTAAAG AGAAATC-TT GAGTTTATGG ACTTCAGGGG GAGTATGGTC ACAAGGCTGA
V9        TTAGAGT-GA CAGAGGTCCG GTAACACT-- -GTTACTGGG ---------C TGACGGA--T CTCGCTTCCA CCTT-ATTCA GAACTTAAAG AGAAATC-TT GAGTTTATGG ACTTCAGGGG GAGTATGGTC ACAAGGCTGA
C329310   TTAGAGT-GA CAGAAGTCCG GTAACACT-- -GTTACTGGG ---------C TGACGGA--T CTCGCTTCCA CCTT-ATTCA GAACTTAAAG AGAAATC-TT GAGTTTATGG ACTTCAGGGG GAGTATGGTC ACAAGGCTGA
SW340     TTAGAGT-GA CAGAAGTCCG GTAACGCT-- -GTTACTGGG ---------T TGACGGA--T CTCGCTTCCA CCTT-ATTCA GAACTTAAAG AGAAATC-TT GAGTTTATGG ACTTCAGGGG GAGTATGGTC ACAAGGCTGA
SW339     TTAGAGT-GA CAGAAGTCCG GTAACGCT-- -GTTACTGGG ---------C TGACGGA--T CTCGCTTCCA CCTT-ATTCA GAACTTAAAG AGAAATC-TT GAGTTTATGG ACTTCAGGGG GAGTATGGTC ACAAGGCTGA
C329314   TTAGAGT-GA CAGAAGTCCG GTAACGCT-- -GTTACTGGG ---------T TGACGGA--T CTCGCTTCCA CCTT-ATTCA GAACTTAAAG AGAAATC-TC GAGTTTATGG ACTTCAGGGG GAGTATGGTC ACAAGGCTGA
SW308     TTAGAGT-GA CAGAAGTCCG GTAACGCT-- -GTTACTGGG ---------T TGACGGA--T CTCGCTTCCA CCTT-ATTCA GAACTTAAAG AGAAATC-TT GAGTTTATGG ACTTCAGGGG GAGTATGGTC ACAAGGCTGA
IC85      TTAGAGT-GA CAGAAGTCCG GTAACGCT-- -GTTACTGGG ---------C TGACGGA--T CTCGCTTCCA CCTT-ATTCA GAACTTAAAG AGAAATC-TT GAGTTTATGG ACTTCAGGGG GAGTATGGTC ACAAGGCTGA
SW318     TTAGAGT-GA CAGAAGTCCG GTAACGCT-- -GTTACTGGG ---------C TGACGGA--T CTCGCTTCCA CCTT-ATTCA GAACTTAAAG AGAAATC-TT GAGTTTATGG ACTTCAGGGG GAGTATGGTC ACAAGGCTGA
SW330     TTAGAGT-GA CAGAAGTCCG GTAACGCT-- -GTTACTGGG ---------C TGACGGA--T CTCGCTTCCA CCTT-ATTCA GAACTTAAAG AGAAATC-TT GAGTTTATGG ACTTCAGGGG GAGTATGGTC ACAAGGCTGA
SW322     TTAGAGT-GA CAGAAGTCCG GTAACGCT-- -GTTACTGGG ---------C TGACGGA--T CTCGCTTCCA CCTT-ATTCA GAACTTAAAG AGAAATC-TT GAGTTTATGG ACTTCAGGGG GAGTATGGTC ACAAGGCTGA
SW324     TTAGAGT-GA CAGAAGTCCG GTAACGCT-- -GTTACTGGG ---------C TGACGGA--T CTCGCTTCCA CCTT-ATTCA GAACTTAAAG AGAAATC-TT GAGTTTATGG ACTTCAGGGG GAGTATGGTC ACAAGGCTGA
SW305     TTAGAGT-GA CAGAAGTCCG GTAACGCT-- -GTTACTGGG ---------C TGACGGA--T CTCGCTTCCA CCTT-ATTCA GAACTTAAAG AGAAATC-TT GAGTTTATGG ACTTCAGGGG GAGTATGGTC ACAAGGCTGA
SW315     TTAGAGT-GA CAGAAGTCCG GTAACGCT-- -GTTACTGGG ---------C TGACGGA--T CTCGCTTCCA CCTT-ATTCA GAACTTAAAG AGAAATC-TT GAGTTTATGG ACTTCAGGGG GAGTATGGTC ACAAGGCTGA
SW320     TTAGAGT-GA CAGAAGTCCG GTAACGCT-- -GATACTGGG ---------C TGACGGA--T CTCGCTTCCA CCTT-ATTCA GAACTTAAAG AGAAATC-TT GAGTTTATGG ACTTCAGGGG GAGTATGGTC ACAAGGCTGA
SW321     TTAGAGT-GA CAGAAGTCCG GTAACGCT-- -GTTACTGGG ---------C TGACGGA--T CTCGCTTCCA CCTT-ATTCA GAACTTAAAG AGAAATC-TT GAGTTTATGG ACTTCAGGGG GAGTATGGTC ACAAGGCTGA
SW341     ATAGAGT-GA CAGAAGTCCG GTAACGCT-- -GATACCGGG ---------C TGACGGA--T CTCGCTTCCA CCTT-ATTCA GAACTTAAAG AGAAATC-TT GAGTTTATGG ACTTCAGGGG GAGTATGGTC ACAAGGCTGA
IC94      TTAGAGT-GA CAGAAGTCCG GTAACGCT-- -GTTACTGGG ---------C TGACGGA--T CTCGCTTCCA CCTT-ATTCA GAACTTAAAG AGAAATC-TT GAGTTTATGG ACTTCAGGGG GAGTATGGTC ACAAGGCTGA
IC92      TTAGAGT-GA CAGAAGTCCG GTAACACT-- -GTTATTGGG ---------C TGACGGA--T CTCGCTTCCA CCTT-ATTCA GAACTTAAAG AGAAATC-TT GAGTTTATGG ACTTCAGGGG GAGTATGGTC ACAAGGCTGA
IC86      TTAGAGT-GA CAGAAGTCCG GCAACGCT-- -GTTGTTGGG ---------C TGACGGA--T CTCGCTTCCA CCTT-ATTCA GAACTTAAAG AGAAATC-TC GAGTTTATGG ACTTCAGGGG GAGTATGGTC ACAAGGCTGA
SW306     TTAGAGT-GA CAGAAGTCCG GCAACGCT-- -GTTGTTGGG ---------C TGACGGA--T CTCGCTTCCA CCTT-ATTCA GAACTTAAAG AGAAATC-TC GAGTTTATGG ACTTCAGGGG GAGTATGGTC ACAAGGCTGA
SW316     TTAGAGT-GA CAGAAGTCCG GCAACGCT-- -GTTGTTGGG ---------C TGACGGA--T CTCGCTTCCA CCTT-ATTCA GAACTTAAAG AGAAATC-TC GAGTTTATGG ACTTCAGGGG GAGTATGGTC ACAAGGCTGA
IC90      TTAGAGT-GA CAGACGTCCG GTAACGCT-- -GTTACTGGG ---------C TGACGGA--T CTCGCTTCCA CCTT-ATTCA GAACTTAAAG AGAAATC-TT GAGTTTATGG ACTTCAGGGG GAGTATGGTC ACAAGGCTGA
SW313     TTAGAGT-GA CAGAAGTCCG GCAACGCT-- -GTTGTCGGG ---------C TGACGGA--T CTCGCTTCCA CCTT-ATTCA GAACTTAAAG AGAAATC-TT GAGTTTATGG ACTTCAGGGG GAGTATGGTC ACAAGGCTGA
SW317     TTAGAGT-GA CAGAAGTCCG GCAACGCT-- -GTTGTTGGG ---------C TGACGGA--T CTCGCTTCCA CCTT-ATTCA GAACTTAAAG AGAAATC-TT GAGTTTATGG ACTTCAGGGG GAGTATGGTC ACAAGGCTGA
SW325     TTAGAGT-GA CAGAAGTCCG GTAACGCT-- -GTTATTGGG ---------C TGACGGA--T CTCGCTTCCA CCTT-ATTCA GAACTTAAAG AGAAATC-TT GAGTTTATGG ACTTCAGGGG GAGTATGGTC ACAAGGCTGA
C329318   TTAGAGT-GA CAGAAGTCCG GTAACACT-- -GTTATTGGG ---------C TGACAGA--T CTCGCTTCCA CCTT-ATTCA GAACTTAAAG AGAAATC-TT GAGTTTATGG ACTTCAGGGG GAGTATGGTC ACAAGGCTGA
N666251   TAAGAGG-GA CAGAAGTCCG GCAACGCT-- -GTTGTTGGG ---------C TGACGGA--C CTCGCTTCCA CCTT-ATTCA GAACTTAAAG AGAAATC-TT GAGTTTATGG ACTTCAGGGG GAGTATGGTC ACAAGGCTGA
IC87      TAAGAGT-GA CAGAAGTCCG GCAACGCT-- -GTTGTTGGG ---------C TGACGGA--C CTCGCTTCCA CCTT-ATTCA GAACTTAAAG AGAAATC-TT GAGTTTATGG ACTTCAGGGG GAGTATGGTC ACAAGGCTGA
SW319     ATAGAGT-GA CAGAAGTCCG GCAACGCT-- -GTTGTCGGG ---------C TGACGGA--C CTCGCTTCCA CCTT-ATTCA GAACTTAAAG AGAAATC-TT GAGTTTATGG ACTTCAGGGG GAGTATGGTC ACAAGGCTGA
SW307     TTAGAGT-GA CAGAAGTCCG GCAACGCT-- -GTTGTCGGG ---------C TGACGGA--C CTCGCTTCCA CCTT-ATTCA GAACTTAAAG AGAAATC-TT GAGTTTATGG ACTTCAGGGG GAGTATGGTC ACAAGGCTGA
N666252   TAAGAGT-GA CAGAAGTCCG GTAACGCT-- -GTTATTGGG ---------C AGACGGA--T CTCGCTTCCA CCTT-ATTCA GAACTTAAAG AGAAATC-TT GAGTTTATGG ACTTCAGGGG GAGTATGGTC ACAAGGCTGA
SW360     AAAGAGT-GA CAGATGCCGA ATAGCATG-- -GCTATCTGG ---------C GGACGGA--A CTCGCTTCCA CCTT-ATTCA GAACTTAAAG AGAAATC-TT GAGTTTATGG ACTTCAGGGG GAGTATGGTC ACAAGGCTGA
SW346     ATAGAGT-GA CAGATGCCGG GTAGCATG-- -GCTATCAGG ---------C GGACGGA--G CTCGCTTCCA CCTT-ATTCA GAACTTAAAG AGAAATC-TT GAGTTTATGG ACTTCAGGGG GAGTATGGTC ACAAGGCTGA
IC82      TTAGAGC-GA CTGATGACCG GTAGCATG-- -GCTATCGGG ---------C TGACGGA--G CTCGCTTCCA CCTT-ATTCA GAACTTAAAG AGAAATC-TT GAGTTTATGG ACTTCAGGGG GAGTATGGTC ACAAGGCTGA
SW328     TTAGAGC-GA CTGAGGACCG GTAGCATG-- -GCTACTGGT ---------T TGAAGAC--G CTCGCTTCCA CCTT-ATTCA GAACTTAAAG AGAAATC-TT GAGTTTATGG ACTTCAGGGG GAGTATGGTC ACAAGGCTGA
IC84      TTAGAGT-GA CAGATGTCCG GTAGCACG-- -GCTATCGGG ---------C TGACGGA--T CTCGCTTCCA CCTT-ATTCA GAACTTAAAG AGAAATC-TT GAGTTTATGG ACTTCAGGGG GAGTATGGTC ACAAGGCTGA
SW329     TTAGAGT-GA CAGATGTCCG GTAGCACG-- -GCTACTGGG ---------T TGACGGA--T CTCGCTTCCA CCTT-ATTCA GAACTTAAAG AGAAATC-TT GAGTTTATGG ACTTCAGGGG GAGTATGGTC ACAAGGCTGA
IC83      TTAGAGT-GA CAGAAGTCCG GTAGCACT-- -GCTACTGGG ---------C TGACGGA--T CTCGCTTCCA CCTT-ATTCA GAACTTAAAG AGAAATC-TT GAGTTTATGG ACTTCAGGGG GAGTATGGTC ACAAGGCTGA
SW304     TTTGAGT-GA CTAAGAGTTA ATAGCATT-- -GCTATTGAC ---------T TGAAGGA--A CTCGCTTTCA CCTT-ATTCA GAACTTAAAG AGAAATC-TT GAGTTTATGG ACTTCAGGGG GAGTATGGTC ACAAGGCTGA
R025406   TTTGAGT-GA CTAAGAGTTA ATAGCATT-- -GCTATTGAC ---------T CGAAGGA--A CTCGCTTTCA CCTT-ATTCA GAACTTAAAG AGAAATC-TT GAGTTTATGG ACTTCAGGGG GAGTATGGTC ACAAGGCTGA
R686358   TTAGGGGAGA CAGAAGGCTG ATATCATTT- -GATATTAGT ---------C TGAAGGA--T CCCGCTTCCA CCTTTATTCA GAACTTAAAG AGAAATC-TT GAGTTTATGG ACTTCAGGGG GAGTATGGTC ACAAGGCTGA
N666253   TTTGGAC-GA CAGAGGTTAG ATAACATT-- -GTTATTTGA ---------C TAACGGA--G TCCGCTTTTA CCTT-ATTCA GAACTTAAAG AGAAATC-TT GAGTTTATGG ACTTCAGGGG GAGTATGGTC ACAAGGCTGA
IC89      TTAGAAC-GA CAGAGGTTAG ATAACATT-- -GTTATTTGA ---------C TAACGGA--G CTCGCTTCTA CCTT-ATTCA GAACTTAAAG AGAAATC-TT GAGTTTATGG ACTTCAGGGG GAGTATGGTC ACAAGGCTGA
R686363   TTAGAAA-TT TCTCGGTCTG GTATCTTC-- -GATATTAGA ---------T TGATAAA--T TTCGCTTCCA CCTT-ATTCA GAACTTAAAG AGAAATC-TT GAGTTTATGG ACTTCAGGGG GAGTATGGTC ACAAGGCTGA
```

```
SW332          ATAGAGT-GA CTGAAGCATT GTATCATT-- -GATACAGTG ---------C TGACGGA--A CTCGCTTCTA CCTT-ATTCA GAACTTAAAG AGAAATC-TT GAGTTTATGG ACTTCAGGGG GAGTATGGTC ACAAGGCTGA
R686361        --ACAAACAG GAAGTTGAAG GGATCATTG- -AT-CTTGGA ---------A ACTTTTC--T GTTAAAATTA CCTT-ATTCA GAACTTAAAG AGAAATC-TT GAGTTTATGG ACTTCAGGGG GAGTATGGTC ACAAGGCTGA
F149909        CAAGAGATCT GAAACACGAA GAATCAACG- -ATTCCTAGT ---------G ATGAAGA--T TTCACTATTA CCTT-ATTCA GAACTTGAAG AGAAATC-TC GAGTTAATAG ACTTCAGGGG GAGTATGGTC ACAAGGCTGA

               ....|....| ....|....| ....|....| ....|....| ....|....| ....|....| ....|....| ....|....| ....|....| ....|....| ....|....| ....|....| ....|....| ....|....|
                   145        155        165        175        185        195        205        215        225        235        245        255        265        275
IC91           TCTGGGG-AT GAGATTATCA AATTCATT-- -GAATTTGAA ---------G ATCAATA--T CTCCGTTCTA ACCT-ATTCA GAACTTAAAG AGAAATC-TT GAGTTTATGG ACTTCAGGGG GAGTATGGTC ACAAGGCTGA
Q286371        AATCGAA-AT GAATAATTGA GTAGCTTTC- -AGCTACAAA ---------A TTAGACA--T TTCATCACTT CCTT-GTTCA GAACKTAAAG AGAAATC-TC GAGTTAATGG ACTTCAGGGG GAGTATGGTC ACAAGGCTGA
F149915        TCGAAG--TA TGAGCCTTTT TCTCCAACTT -GTTGGAAAA ------AAAG CGTTTCG--C TTCATTTCTT CCCTTATTCA GGACTTGATG AGAAATCATT AAGTGAATGG ACTTCAGGGG GAGTATGGTC ACAAGGCTGA
B445018        TGAGAGGTCA CTTTACTTTT CTTTCATTTT CGTCAGAAAA TGGATGAAAT TGGTGGGTTC TCTATATCTT CCCTTATTCA GAACTTGATG AGAAATCATT AAGTTAATGG ACTTCAGGGG GAGTATGGTC ACAAGGCTGA
F149910        AAAGAAATGA GAAGATTCTG ATGATCGAT- -GAT-CAGAA G------AGT TTTATTA--T TTCAATATTA CCTT-ATTTA GCACTTAAAG AGAAATC-TT GAGTTACTAG ACTTCAGGGG GAGTATGGTC ACAAGGCTGA
F149907        TTAAGACTGA TAA-----TG ATATTCAGC- -ATTGCTGGA T------AAC ATTTGAA--G TCGAATAC-A CCTT-ATTCA GAACTTGAAG AGAAATC-TT GAGTTATTGG ACTTCAGGGG GAGTATGGTC ACAAGGCTGA
X027294        ATT---TCTT AGTTACATAA GTAACGTGTT ACTTTGTGAC A--------T AAGAAATTAA TACATAAT-A CTTT-ATTCA GAACTTGAAG AGAAATCT-T GAGTGTATGA ACTTCAGGGG GAGTATGGTC ACAAGGCTGA
Q286372        ATTGAATTAA ATTTAAATGA TTAACTTGTT AATTGTTTAA A--------T TTAATTTAAT AATATAATCA CTTT-ATTCA GAACTTGAAG AGAAATCTAT GAGTTTATGA ACTTCAGGGG GAGTATGGTC ACAAGGCTGA
F149913        A--------- ----GAAAGA TTAATT--TT CATTTTGGAA A--------T TGATTTTTAT --TGTTACTC TCTT-ATTCA GGATTTAAAG GGAAACCTTT AAGTGAATGG ACTTCAGGGG GAGTATGGTC ACAAGGCTGA
Y769863        GGGAACCTCT --TTTTTATT TCGGTAAGAA ATGTTTCCGA A--------T TGTTAGGTAT AACAATCAAG GCCT-CTGTG GGATTCAAGG AGAAATCCTT GAGTCTCTAC ATTTCAGGGG GAGTATGGTT GCAAGGCTGA
F149908        AGGTA----- ---------- TAGGAATGAA AATTTTCTAT A--------C CACTATTT-T AATAC----- -CCT-TTGCA GGACT-ATAG AGAAATCAAG AAGTTCCTGC ATTTCAGGGG GAGTATGGTC ACAAGGCTGA
onsensus                                                                                       *  *    * *      *  **** *    ***   *   * ******** *********   *********

               ....|....| ....|....| ....|....| ....|....| ....|....| ....|....| ....|....| ....|....| ....|....| ....|....| ....|....| ....|....| ....|....| ....|....|
                   285        295        305        315        325        335        345        355        365        375        385        395        405        415
64142          AACTTAAAGG AATTGACGGA AGGGCACACC AGGAGTGGAG CCTGCG-CTT AATTTGACTC AACACGGGAA AACTTACCAA GACCGAACAG TAGAAGGAAT GACAGATTAA GAGTTCTTTC ATGATTTATT GGGTAGTGGT
R686356        AACTTAAAGG AATTGACGGA AGGGCACACC AGGAGTGGAG CCTGCGGCTT AATTTGACTC AACACGGGAA AACTTACCAA GACCGAACAG TAGAAGGAAT GACAGATTAA GAGTTCTTTC ATGATTTATT GGGTAGTGGT
R025411        AACTTAAAGG AATTGACGGA AGGGCACACC AGGAGTGGAG CCTGCGGCTT AATTTGACTC AACACGGGAA AACTTACCAA GACCGAACAG TAGAAGGAAT GACAGATTAA GAGTTCTTTC ATGATTTATT GGGTAGTGGT
Q286373        AACTTAAAGG AATTGACGGA AGGGCACACC AGGAGTGGAG CCTGCGGCTT AATT-GACTC AACACGGGAA AACTTACCAA GACCGAACAG TAGAAGGAAT GACAGATTAA TAGTTCTTTC ATGATTTATT GGGTAGTGGT
F149906        AACTTAAAGG AATTGACGGA AGGGCACACC AGGAGTGGAG CCTGCGGCTT AATTTGACTC AACACGGGAA AACTTACCAA GACCGAACAG TAGAAGGAAT GACAGATTAA GAGTTCTTTC ATGATTTATT GGGTAGTGGT
SW344          AACTTAAAGG AATTGACGGA AGGGCACACC AGGAGTGGAG CCTGCGGCTT AATTTGACTC AACACGGGAA AACTTACCAA GACCGAACAG TATTAGGAAT GACAGATTAA GGGTTCTTTC ATGATTTATT GGGTAGTGGT
SW343          AACTTAAAGG AATTGACGGA AGGGCACACC AGGAGTGGAG CCTGCGGCTT AATTTGACTC AACACGGGAA AACTTACCAA GACCGAACAG TATTAGGAAT GACAGATTAA GGGTTCTTTC ATGATTTATT GGGTAGTGGT
N666250        AACTTAAAGG AATTGACGGA AGGGCACACC AGGAGTGGAG CCTGCGGCTT AATTTGACTC AACACGGGAA AACTTACCAA GACCGAACAG TATTAGGAAT GACAGATTAA GGGTTCTTTC ATGATTTATT GGGTAGTGGT
C329307        AACTTAAAGG AATTGACGGA AGGGCACACC AGGAGTGGAG CCTGCGGCTT AATTTGACTC AACACGGGAA AACTTACCAA GACCGAACAG TATTAGGAAT GACAGATTAA GGGTTCTTTC ATGATTTATT GGGTAGTGGT
SW312          AACTTAAAGG AATTGACGGA AGGGCACACC AGGAGTGGAG CCTGCGGCTT AATTTGACTC AACACGGGAA AACTTACCAA GACCGAACAG TATTAGGAAT GACAGATTAA GGGTTCTTTC ATGATTTATT GGGTAGTGGT
C329309        AACTTAAAGG AATTGACGGA AGGGCACACC AGGAGTGGAG CCTGCGGCTT AATTTGACTC AACACGGGAA AACTTACCAA GACCGAACAG TATTAGGAAT GACAGATTAA GGGTTCTTTC ATGATTTATT GGGTAGTGGT
SW327          AACTTAAAGG AATTGACGGA AGGGCACACC AGGAGTGGAG CCTGCGGCTT AATTTGACTC AACACGGGAA AACTTACCAA GACCGAACAG TATTAGGAAT GACAGATTAA GGGTTCTTTC ATGATTTATT GGGTAGTGGT
SW326          AACTTAAAGG AATTGACGGA AGGGCACACC AGGAGTGGAG CCTGCGGCTT AATTTGACTC AACACGGGAA AACTTACCAA GACCGAACAG TATTAGGAAT GACAGATTAA GGGTTCTTTC ATGATTTATT GGGTAGTGGT
SW331          AACTTAAAGG AATTGACGGA AGGGCACACC AGGAGTGGAG CCTGCGGCTT AATTTGACTC AACACGGGAA AACTTACCAA GACCGAACAG TATTAGGAAT GACAGATTAA GGGTTCTTTC ATGATTTATT GGGTAGTGGT
SW349          AACTTAAAGG AATTGACGGA AGGGCACACC AGGAGTGGAG CCTGCGGCTT AATTTGACTC AACACGGGAA AACTTACCAA GACCGAACAG TATTAGGAAT GACAGATTAA GGGTTCTTTC ATGATTTATT GGGTAGTGGT
IC88           AACTTAAAGG AATTGACGGA AGGGCACACC AGGAGTGGAG CCTGCGGCTT AATTTGACTC AACACGGGAA AACTTACCAA GACCGAACAG TATTAGGAAT GACAGATTAA GGGTTCTTTC ATGATTTATT GGGTAGTGGT
SW350          AACTTAAAGG AATTGACGGA AGGGCACACC AGGAGTGGAG CCTGCGGCTT AATTTGACTC AACACGGGAA AACTTACCAA GACCGAACAG TATTAGGAAT GACAGATTAA GGGTTCTTTC ATGATTTATT GGGTAGTGGT
SW345          AACTTAAAGG AATTGACGGA AGGGCACACC AGGAGTGGAG CCTGCGGCTT AATTTGACTC AACACGGGAA AACTTACCAA GACCGAACAG TATTAGGAAT GACAGATTAA GGGTTCTTTC ATGATTTATT GGGTAGTGGT
Y012747        AACTTAAAGG AATTGACGGA AGGGCACACC AGGAGTGGAG CCTGCGGCTT AATTTGACTC AACACGGGAA AACTTACCAA GACCGAACAG TATTAGGAAT GACAGATTAA GGGTTCTTTC ATGATTTATT GGGTAGTGGT
Y012746        AACTTAAAGG AATTGACGGA AGGGCACACC AGGAGTGGAG CCTGCGGCTT AATTTGACTC AACACGGGAA AACTTACCAA GACCGAACAG TATTAGGAAT GACAGATTAA GGGTTCTTTC ATGATTTATT GGGTAGTGGT
Y012748        AACTTAAAGG AATTGACGGA AGGGCACACC AGGAGTGGAG CCTGCGGCTT AATTTGACTC AACACGGGAA AACTTACCAA GACCGAACAG TATTAGGAAT GACAGATTAA GGGTTCTTTC ATGATTTATT GGGTAGTGGT
N4             AACTTAAAGG AATTGACGGA AGGGCACACC AGGAGTGGAG CCTGCGGCTT AATTTGACTC AACACGGGAA AACTTACCAA GACCGAACAG TATTAGGAAT GACAGATTAA GGGTTCTTTC ATGATTTATT GGGTAGTGGT
N3             AACTTAAAGG AATTGACGGA AGGGCACACC AGGAGTGGAG CCTGCGGCTT AATTTGACTC AACACGGGAA AACTTACCAA GACCGAACAG TATTAGGAAT GACAGATTAA GGGTTCTTTC ATGATTTATT GGGTAGTGGT
N7             AACTTAAAGG AATTGACGGA AGGGCACACC AGGAGTGGAG CCTGCGGCTT AATTTGACTC AACACGGGAA AACTTACCAA GACCGAACAG TATTAGGAAT GACAGATTAA GGGTTCTTTC ATGATTTATT GGGTAGTGGT
N2             AACTTAAAGG AATTGACGGA AGGGCACACC AGGAGTGGAG CCTGCGGCTT AATTTGACTC AACACGGGAA AACTTACCAA GACCGAACAG TATTAGGAAT GACAGATTAA GGGTTCTTTC ATGATTTATT GGGTAGTGGT
V10            AACTTAAAGG AATTGACGGA AGGGCACACC AGGAGTGGAG CCTGCGGCTT AATTTGACTC AACACGGGAA AACTTACCAA GACCGAACAG TATTAGGAAT GACAGATTAA GGGTTCTTTC ATGATTTATT GGGTAGTGGT
V3             AACTTAAAGG AATTGACGGA AGGGCACACC AGGAGTGGAG CCTGCGGCTT AATTTGACTC AACACGGGAA AACTTACCAA GACCGAACAG TATTAGGAAT GACAGATTAA GGGTTCTTTC ATGATTTATT GGGTAGTGGT
N8             AACTTAAAGG AATTGACGGA AGGGCACACC AGGAGTGGAG CCTGCGGCTT AATTTGACTC AACACGGGAA AACTTACCAA GACCGAACAG TATTAGGAAT GACAGATTAA GGGTTCTTTC ATGATTTATT GGGTAGTGGT
N666249        AACTTAAAGG AATTGACGGA AGGGCACACC AGGAGTGGAG CCTGCGGCTT AATTTGACTC AACACGGGAA AACTTACCAA GACCGAACAG TATTAGGAAT GACAGATTAA GGGTTCTTTC ATGATTTATT GGGTAGTGGT
N666248        AACTTAAAGG AATTGACGGA AGGGCACACC AGGAGTGGAG CCTGCGGCTT AATTTGACTC AACACGGGAA AACTTACCAA GACCGAACAG TATTAGGAAT GACAGATTAA GGGTTCTTTC ATGATTTATT GGGTAGTGGT
C329316        AACTTAAAG- AATTGACGGA AGGGCACACC AGGAGTGGAG CCTGCGGCTT AATTTGACTC AACACGGGAA AACTTACCAA GACCGAACAG TATTAGGAAT GACAGATTAA GGGTTCTTTC ATGATTTATT GGGTAGTGGT
C329308        AACTTAAAGG AATTGACGGA AGGGCACACC AGGAGTGGAG CCTGCGGCTT AATTTGACTC AACACGGGAA AACTTACCAA GACCGAACAG TATTAGGAAT GACAGATTAA GGGTTCTTTC ATGATTTATT GGGTAGTGGT
V14            AACTTAAAGG AATTGACGGA AGGGCACACC AGGAGTGGAG CCTGCGGCTT AATTTGACTC AACACGGGAA AACTTACCAA GACCGAACAG TATTAGGAAT GACAGATTAA GGGTTCTTTC ATGATTTATT GGGTAGTGGT
V7             AACTTAAAGG AATTGACGGA AGGGCACACC AGGAGTGGAG CCTGCGGCTT AATTTGACTC AACACGGGAA AACTTACCAA GACCGAACAG TATTAGGAAT GACAGATTAA GGGTTCTTTC ATGATTTATT GGGTAGTGGT
V5             AACTTAAAGG AATTGACGGA AGGGCACACC AGGAGTGGAG CCTGCGGCTT AATTTGACTC AACACGGGAA AACTTACCAA GACCGAACAG TATTAGGAAT GACAGATTAA GGGTTCTTTC ATGATTTATT GGGTAGTGGT
N6             AACTTAAAGG AATTGACGGA AGGGCACACC AGGAGTGGAG CCTGCGGCTT AATTTGACTC AACACGGGAA AACTTACCAA GACCGAACAG TATTAGGAAT GACAGATTAA GGGTTCTTTC ATGATTTATT GGGTAGTGGT
N1             AACTTAAAGG AATTGACGGA AGGGCACACC AGGAGTGGAG CCTGCGGCTT AATTTGACTC AACACGGGAA AACTTACCAA GACCGAACAG TATTAGGAAT GACAGATTAA GGGTTCTTTC ATGATTTATT GGGTAGTGGT
N5             AACTTAAAGG AATTGACGGA AGGGCACACC AGGAGTGGAG CCTGCGGCTT AATTTGACTC AACACGGGAA AACTTACCAA GACCGAACAG TATTAGGAAT GACAGATTAA GGGTTCTTTC ATGATTTATT GGGTAGTGGT
V9             AACTTAAAGG AATTGACGGA AGGGCACACC AGGAGTGGAG CCTGCGGCTT AATTTGACTC AACACGGGAA AACTTACCAA GACCGAACAG TATTAGGAAT GACAGATTAA GGGTTCTTTC ATGATTTATT GGGTAGTGGT
C329310        AACTTAAAGG AATTGACGGA AGGGCACACC AGGAGTGGAG CCTGCGGCTT AATTTGACTC AACACGGGAA AACTTACCAA GACCGAACAG TATTAGGAAT GACAGATTAA GGGTTCTTTC ATGATTTATT GGGTAGTGGT
SW340          AACTTAAAGG AATTGACGGA AGGGCACACC AGGAGTGGAG CCTGCGGCTT AATTTGACTC AACACGGGAA AACTTACCAA GACCGAACAG TATTAGGAAT GACAGATTAA GGGTTCTTTC ATGATTTATT GGGTAGTGGT
SW339          AACTTAAAGG AATTGACGGA AGGGCACACC AGGAGTGGAG CCTGCGGCTT AATTTGACTC AACACGGGAA AACTTACCAA GACCGAACAG TATTAGGAAT GACAGATTAA GGGTTCTTTC ATGATTTATT GGGTAGTGGT
```

```
ISW308    AACTTAAAGG AATTGACGGA AGGGCACACC AGGAGTGGAG CCTGCGGCTT AATTTGACTC AACACGGGAA AACTTACCAA GACCGAACAG TATTAGGAAT GACAGATTAA GGGTTCTTTC ATGATTTATT GGGTAGTGGT
VIC85     AACTTAAAGG AATTGACGGA AGGGCACACC AGGAGTGGAG CCTGCGGCTT AATTTGACTC AACACGGGAA AACTTACCAA GACCGAACAG TATTAGGAAT GACAGATTAA GGGTTCTTTC ATGATTTATT GGGTAGTGGT
ISW318    AACTTAAAGG AATTGACGGA AGGGCACACC AGGAGTGGAG CCTGCGGCTT AATTTGACTC AACACGGGAA AACTTACCAA GACCGAACAG TATTAGGAAT GACAGATTAA GGGTTCTTTC ATGATTTATT GGGTAGTGGT
ISW330    AACTTAAAGG AATTGACGGA AGGGCACACC AGGAGTGGAG CCTGCGGCTT AATTTGACTC AACACGGGAA AACTTACCAA GACCGAACAG TATTAGGAAT GACAGATTAA GGGTTCTTTC ATGATTTATT GGGTAGTGGT
ISW322    AACTTAAAGG AATTGACGGA AGGGCACACC AGGAGTGGAG CCTGCGGCTT AATTTGACTC AACACGGGAA AACTTACCAA GACCGAACAG TATTAGGAAT GACAGATTAA GGGTTCTTTC ATGATTTATT GGGTAGTGGT
ISW324    AACTTAAAGG AATTGACGGA AGGGCACACC AGGAGTGGAG CCTGCGGCTT AATTTGACTC AACACGGGAA AACTTACCAA GACCGAACAG TATTAGGAAT GACAGATTAA GGGTTCTTTC ATGATTTATT GGGTAGTGGT
ISW305    AACTTAAAGG AATTGACGGA AGGGCACACC AGGAGTGGAG CCTGCGGCTT AATTTGACTC AACACGGGAA AACTTACCAA GACCGAACAG TATTAGGAAT GACAGATTAA GGGTTCTTTC ATGATTTATT GGGTAGTGGT
ISW315    AACTTAAAGG AATTGACGGA AGGGCACACC AGGAGTGGAG CCTGCGGCTT AATTTGACTC AACACGGGAA AACTTACCAA GACCGAACAG TATTAGGAAT GACAGATTAA GGGTTCTTTC ATGATTTATT GGGTAGTGGT
ISW320    AACTTAAAGG AATTGACGGA AGGGCACACC AGGAGTGGAG CCTGCGGCTT AATTTGACTC AACACGGGAA AACTTACCAA GACCGAACAG TATTAGGAAT GACAGATTAA GGGTTCTTTC ATGATTTATT GGGTAGTGGT
ISW321    AACTTAAAGG AATTGACGGA AGGGCACACC AGGAGTGGAG CCTGCGGCTT AATTTGACTC AACACGGGAA AACTTACCAA GACCGAACAG TATTAGGAAT GACAGATTAA GGGTTCTTTC ATGATTTATT GGGTAGTGGT
ISW341    AACTTAAAGG AATTGACGGA AGGGCACACC AGGAGTGGAG CCTGCGGCTT AATTTGACTC AACACGGGAA AACTTACCAA GACCGAACAG TATTAGGAAT GACAGATTAA GGGTTCTTTC ATGATTTATT GGGTAGTGGT
VIC94     AACTTAAAGG AATTGACGGA AGGGCACACC AGGAGTGGAG CCTGCGGCTT AATTTGACTC AACACGGGAA AACTTACCAA GACCGAACAG TATTAGGAAT GACAGATTAA GGGTTCTTTC ATGATTTATT GGGTAGTGGT
VIC92     AACTTAAAGG AATTGACGGA AGGGCACACC AGGAGTGGAG CCTGCGGCTT AATTTGACTC AACACGGGAA AACTTACCAA GACCGAACAG TATTAGGAAT GACAGATTAA GGGTTCTTTC ATGATTTATT GGGTAGTGGT
VIC86     AACTTAAAGG AATTGACGGA AGGGCACACC AGGAGTGGAG CCTGCGGCTT AATTTGACTC AACACGGGAA AACTTACCAA GACCGAACAG TATTAGGAAT GACAGATTAA GGGTTCTTTC ATGATTTATT GGGTAGTGGT
ISW306    AACTTAAAGG AATTGACGGA AGGGCACACC AGGAGTGGAG CCTGCGGCTT AATTTGACTC AACACGGGAA AACTTACCAA GACCGAACAG TATTAGGAAT GACAGATTAA GGGTTCTTTC ATGATTTATT GGGTAGTGGT
ISW316    AACTTAAAGG AATTGACGGA AGGGCACACC AGGAGTGGAG CCTGCGGCTT AATTTGACTC AACACGGGAA AACTTACCAA GACCGAACAG TATTAGGAAT GACAGATTAA GGGTTCTTTC ATGATTTATT GGGTAGTGGT
VIC90     AACTTAAAGG AATTGACGGA AGGGCACACC AGGAGTGGAG CCTGCGGCTT AATTTGACTC AACACGGGAA AACTTACCAA GACCGAACAG TATTAGGAAT GACAGATTAA GGGTTCTTTC ATGATTTATT GGGTAGTGGT
ISW313    AACTTAAAGG AATTGACGGA AGGGCACACC AGGAGTGGAG CCTGCGGCTT AATTTGACTC AACACGGGAA AACTTACCAA GACCGAACAG TATTAGGAAT GACAGATTAA GGGTTCTTTC ATGATTTATT GGGTAGTGGT
ISW317    AACTTAAAGG AATTGACGGA AGGGCACACC AGGAGTGGAG CCTGCGGCTT AATTTGACTC AACACGGGAA AACTTACCAA GACCGAACAG TATTAGGAAT GACAGATTAA GGGTTCTTTC ATGATTTATT GGGTAGTGGT
ISW325    AACTTAAAGG AATTGACGGA AGGGCACACC AGGAGTGGAG CCTGCGGCTT AATTTGACTC AACACGGGAA AACTTACCAA GACCGAACAG TATTAGGAAT GACAGATTAA GGGTTCTTTC ATGATTTATT GGGTAGTGGT
.C329318  AACTTAAAGG AATTGACGGA AGGGCACACC AGGAGTGGAG CCTGCGGCTT AATTTGACTC AACACGGGAA AACTTACCAA GACCGAACAG TATTAGGAAT GACAGATTAA GGGTTCTTTC ATGATTTATT GGGTAGTGGT
'N666251  AACTTAAAGG AATTGACGGA AGGGCACACC AGGAGTGGAG CCTGCGGCTT AATTTGACTC AACACGGGAA AACTTACCAA GACCGAACAG TATTAGGAAT GACAGGTTAA GGGTCCTTTC ATGATATATT GGGTAGTGGT
VIC87     AACTTAAAGG AATTGACGGA AGGGCACACC AGGAGTGGAG CCTGCGGCTT AATTTGACTC AACACGGGAA AACTTACCAA GACCGAACAG TATTAGGAAT GACAGATTAA GGGTTCTTTC ATGATTTATT GGGTAGTGGT
ISW319    AACTTAAAGG AATTGACGGA AGGGCACACC AGGAGTGGAG CCTGCGGCTT AATTTGACTC AACACGGGAA AACTTACCAA GACCGAACAG TATTAGGAAT GACAGATTAA GGGTCCTTTC ATGATTTATT GGGTAGTGGT
ISW307    AACTTAAAGG AATTGACGGA AGGGCACACC AGGAGTGGAG CCTGCGGCTT AATTTGACTC AACACGGGAA AACTTACCAA GACCGAACAG TATTAGGAAT GACAGATTAA GGGTCCTTTC ATGATTTATT GGGTAGTGGT
'N666252  AACTTAAAGG AATTGACGGA AGGGCACACC AGGAGTGGAG CCTGCGGCTT AATTTGACTC AACACGGGAA AACTTACCAA GACCGAACAG TATTAGGAAT GACAGATTAA GGGTTCTTTC ATGATTTATT GGGTAGTGGT
ISW360    AACTTAAAGG AATTGACGGA AGGGCACACC AGGAGTGGAG CCTGCGGCTT AATTTGACTC AACACGGGAA AACTTACCAA GACCGAACAG TATTAGGAAT GACAGATTAA AGGTTTTTTC ATGATTTATT GGGTAGTGGT
ISW346    AACTTAAAGG AATTGACGGA AGGGCACACC AGGAGTGGAG CCTGCGGCTT AATTTGACTC AACACGGGAA AACTTACCAA GACCGAACAG TATTAGGAAT GACAGATTAA AGGTTTTTTC ATGATTTATT GGGTAGTGGT
VIC82     AACTTAAAGG AATTGACGGA AGGGCACACC AGGAGTGGAG CCTGCGGCTT AATTTGACTC AACACGGGAA AACTTACCAA GACCGAACAG TATTAGGAAT GACAGATTAA GGGTTCTTTC ATGATTTATT GGGTAGTGGT
ISW328    AACTTAAAGG AATTGACGGA AGGGCACACC AGGAGTGGAG CCTGCGGCTT AATTTGACTC AACACGGGAA AACTTACCAA GACCGAACAG TATTAGGAAT GACAGATTAA GGGTTCTTTC ATGATTTATT GGGTAGTGGT
VIC84     AACTTAAAGG AATTGACGGA AGGGCACACC AGGAGTGGAG CCTGCGGCTT AATTTGACTC AACACGGGAA AACTTACCAA GACCGAACAG TATTAGGAAT GACAGATTAA GGGTTCTTTC ATGATTTATT GGGTAGTGGT
ISW329    AACTTAAAGG AATTGACGGA AGGGCACACC AGGAGTGGAG CCTGCGGCTT AATTTGACTC AACACGGGAA AACTTACCAA GACCGAACAG TATTAGGAAT GACAGATTAA GGGTTCTTTC ATGATTTATT GGGTAGTGGT
VIC83     AACTTAAAGG AATTGACGGA AGGGCACACC AGGAGTGGAG CCTGCGGCTT AATTTGACTC AACACGGGAA AACTTACCAA GACCGAACAG TATTAGGAAT GACAGATTAA GGGTTCTTTC ATGATTTATT GGGTAGTGGT
ISW304    AACTTAAAGG AATTGACGGA AGGGCACACC AGGAGTGGAG CCTGCGGCTT AATTTGACTC AACACGGGAA AACTTACCAA GACCGAACAG TATTAGGAAT GACAGATTAA GGGTTCTTTC ATGATTTATT GGGTAGTGGT
IR025406  AACTTAAAGG AATTGACGGA AGGGCACACC AGGAGTGGAG CCTGCGGCTT AATTTGACTC AACACGGGAA AACTTACCAA GACCGAACAG TATTAGGAAT GACAGATTAA GGGTTCTTTC ATGATTTATT GGGTAGTGGT
'R686358  AACTTAAAGG AATTGACGGA AGGGCACACC AGGAGTGGAG CCTGCGGCTT AATTTGACTC AACACGGGAA AACTTACCAA GACCGAACAG TATTAGGAAT GACAGATTAA GGGTTCTTTC ATGATTTATT GGGTAGTGGT
'N666253  AACTTAAAGG AATTGACGGA AGGGCACACC AGGAGTGGAG CCTGCGGCTT AATTTGACTC AACACGGGAA AACTTACCAA GACCGAACAG TATTAGGAAT GACAGATTAA GGGTTCTTTC ATGATTTATT GGGTAGTGGT
VIC89     AACTTAAAGG AATTGACGGA AGGGCACACC AGGAGTGGAG CCTGCGGCTT AATTTGACTC AACACGGGAA AACTTACCAA GACCGAACAG TATTAGGAAT GACAGATTAA GGGTTCTTTC ATGATTTATT GGGTAGTGGT
'R686363  AACTTAAAGG AATTGACGGA AGGGCACACC AGGAGTGGAG CCTGCGGCTT AATTTGACTC AACACGGGAA AACTTACCAA GACCGAACAG GAGTAGGAAT GACAGATTAA GGGTTCTTTC ATGATTTTCT GGGTAGTGGT
VIC93     AACTTAAAGG AATTGACGGA AGGGCACACC AGGAGTGGAG CCTGCGGCTT AATTTGACTC AACACGGGAA AACTTACCAA GACCGAACAG TATTAGGAAT GACAGGTTAA GGGTCCTTTC ATGATTTATT GGGTAGTGGT
ISW332    AACTTAAAGG AATTGACGGA AGGGCACACC AGGAGTGGAG CCTGCGGCTT AATTTGACTC AACACGGGAA AACTTACCAA GACCGAACAG TATTAGGAAT GACAGGTTAA GGGTCCTTTC ATGATTTATT GGGTAGTGGT
'R686361  AACTTAAAGG AATTGACGGA AGGGCACACC AGGAGTGGAG CCTGCGGCTT AATTTGACTC AACACGGGAA AACTTACCAA GACCGAACAG TAGTAGGAAT GACAGATTAA TGGTTCTTTC ATGATTTATT GGGTAGTGGT
.F149909  AACTTAAAGG AATTGACGGA AGGGCACACC AGGAGTGGAG CCTGCGGCTT AATTTGACTC AACACGGGAA AACTTACCAA GACCGAACAT TATAAGGAAT GACAGATTAA GAGTTCTTTC ATGATTTAAT GGGGTGTGGT
VIC91     AACTTAAAGG AATTGACGGA AGGGCACACC AGGAGTGGAG CCTGCGGCTT AATTTGACTC AACACGGGAA AACTTACCAA GACCGAACGG TGTTAGGAAT GACAGGTTAA TGGTTCTTTC ATGATTCATC GGGTAGTGGT
)Q286371  AACTTAAAGG AATTGACGGA AGGGCACACC AGGAGTGGAG CCTGCGGCTT AATTTGACTC AACACGGGAA AACTTACCAA GACCGAACAT TAGAAGGATT GACAGGTTAA GGGTCCTTTC AAGATTTGAT GGGTAGTGGT
.F149915  AACTTAAAGG AATTGACGGA AGGGCACACC AGGAGTGGAG CCTGCGGCTT AATTTGACTC AACACGGGAA AACTTACCAA GACCAAACGG TGTAAGGAAT GACAGATTAC TGGGTCTTTC ATGATTCATC GGGGAGTGGT
.B445018  AACTTAAAGA AATTGACGGA AGGGCACACC AGGAGTGGAG CCTGCGGCTT AATTTGACTC AACACGGGAA AACTTACCAA GACCAAACGG TGTTAGGAAT GACAGGTTAC TGGATCTTTC ATGATTCATC GGGGAGTGGT
.F149910  AACTTAAAGG AATTGACGGA AGGGCACACC AGGAGTGGAG CCTGCGGCTT AATTTGACTC AACACGGGAA AACTTACCAA GACCGAACAG TATTAGGAAT GACAGATTAA GGGTTCTTTC ATGATTTATT GGCGAGTGGT
.F149907  AACTTAAAGG AATTGACGGA AGGGCACACA AGGAGTGGAG CCTGCGGCTT AATTTGACTC AACACGGGAA AACTTACCAA GACCGAACAT TATGAGGAAT GACAGATTAA GAGATCTTTC ATGATTTAAT GGTGAGTGGT
IX027294  AACTTAAAGG AATTGACGGA AGGGCACACC AGGAGTGGAG CCTGCGGCTT AATTTGACTC AACACGGGAA AACTTACCAA GACCGAACAT ACTAAGGAAT GACAGATTAA AGGATTTTTC ATGATTGTAT GGGGAGTGGT
)Q286372  AACTTAAAGG AATTGACGGA AGGGCACACC AGGAGTGGAG CCTGCGGCTT AATTTGACTC AACACGGGAA AACTTACCAA GACCGAACAT AATAAGGAAT GACAGATTAA AGGTTCTTTC ATGATTTTAT GGGGAGTGGT
.F149913  AACTTAAAGG AATTGACGGA AGGGCACACC AGGAGTGGAG CCTGCGGCTT AATTTGACTC AACACGGGAA AACTTACCAA GACCGTACAT TAAAAGGATT GACAGATTAA TAGTTTTTTC ATGATATGAT GGTGAGTGGT
.Y769863  AACTTAAAGG AATTGACGGA AGGGCACACC AGGAGTGGAG CCTGCGGCTT AATTTGACTC AACACGGGAA AACTTACCAA GACCGAACAT TAGAGGGATT GACAGATTTA GGGTTCTTTC AAGATTTAAA GAGGAGTGGT
.F149908  AACTTAAAGG AATTGACGGA AGGGCACACC AGGAGTGGAG CCTGCGGCTT AATTTGACTC AACACGGGAA AACTTACCAA GACCGAACAT TAGAGGGAAT GACAGATTAA GGGTTCTTTC ATGATTTAAA GGGGAGTGGT
Consensus *********  ********** *********  ********** ****** *** **** ***** ********** ********** ****  **         *** * ***** **     *   **** * ***      *    *****


          ....|....| ....|....| ....|....| ....|....| ....|....| ....|....| ....|....| ....|....| ....|....| ....|....| ....|....| ....|....| ....|....| ....|....|
                425        435        445        455        465        475        485        495        505        515        525        535        545        555
I64142    GCATGGCCGT TCTTAGTTGG TGGAGTGATT TGTCAGGTTA ATTCCGGTAA CGAACGAGAC TGAAACCTAT TAATTAGTTT TCTG------ CCTATAAGAC AGAAATGTTC GCAA------ ---GAACAGG TGC-GTAA--
'R686356  GCATGGCCGT TCTTAGTTGG TGGAGTGATT TGTCAGGTTA ATTCCGGTAA CGAACGAGAC TGAAACCTAT TAATTAGTTT TCTG------ CCTATAAGAC AGAAATGTTC GCAA------ ---GAACAGG TGC-GTAA--
IR025411  GCATGGCCGT TCTTAGTTGG TGGAGTGATT TGTCAGGTTA ATTCCGGTAA CGAACGAGAC TGAAACCTAT TAATTAGTTT TTTG------ CCTATAAGAC AAGAATGTTC GTAA------ ---GGACAGG TGCCGCAA--
)Q286373  GCATGGCCGT TCTTAGTTGG TGGAGTGATT TGTCAGGTTA ATTCCGGTAA CGAACGAGAC TGAAACCTAT TAATTAGTTT TTTG------ CCTATAAGAC AAGAATGTTC GTAA------ ---GGACAGG TGC-GCAA--
```

```
SW344    GCATGGCCGT TCTTAGTTGG TGGAGTGATT TGTCAGGTTA ATTCCGGTAA CGAACGAGAC TGAAGCCTAT TAATTAGTTT CTCG------ CCTATAAGAC GAGAATGTCC GCAA------ ---GGATGGA TGCTTTT---
SW343    GCATGGCCGT TCTTAGTTGG TGGAGTGATT TGTCAGGTTA ATTCCGGTAA CGAACGAGAC TGAAGCCTAT TAATTAGTTT CTCG------ CCTATAAGAC GAGAATGTCC GCAA------ ---GGATGGA TGCTTTT---
N666250  GCATGGCCGT TCTTAGTTGG TGGAGTGATT TGTCAGGTTA ATTCCGGTAA CGAACGAGAC TGAAGCCTAT TAATTAGTTT CTCG------ CCTATAAGAC GAGAATGTCC GCAA------ ---GGATGGA TGCTTTT---
C329307  GCATGGCCGT TCTTAGTTGG TGGAGTGATT TGTCAGGTTA ATTCCGGTAA CGAACGAGAC TGAAGCCTAT TAATTAGTTT CTCG------ CCTATAAGAC GAGAATGTCC GCAT------ ---GGATGGA TGCTTTT---
SW312    GCATGGCCGT TCTTAGTTGG TGGAGTGATT TGTCAGGTTA ATTCCGGTAA CGAACGAGAC TGAAGCCTAT TAATTAGTTT CTCG------ CCTATAAGAC GAGAATGTCC GCAA------ ---GGATGGA TGCTTTT---
C329309  GCATGGCCGT TCTTAGTTGG TGGAGTGATT TGTCAGGTTA ATTCCGGTAA CGAACGAGAC TGAAGCCTAT TAATTAGTTT CTCG------ CCTATAAGAC GAGAATGTCC GCAA------ ---GGATGGA TGCTTTT---
SW327    GCATGGCCGT TCTTAGTTGG TGGAGTGATT TGTCAGGTTA ATTCCGGTAA CGAACGAGAC TGAAGCCTAT TAATTAGTTT CTCG------ CCTATACGAC GAGAATGTCC GCAA------ ---GGATGGA TGCTTTT---
SW326    GCATGGCCGT TCTTAGTTGG TGGAGTGATT TGTCAGGTTA ATTCCGGTAA CGAACGAGAC TGAAGCCTAT TAATTAGTTT CTCG------ CCTATACGAC GAGAATGTCC GCAA------ ---GGATGGA TGCTTTT---
SW331    GCATGGCCGT TCTTAGTTGG TGGAGTGATT TGTCAGGTTA ATTCCGGTAA CGAACGAGAC TGAAGCCTAT TAATTAGTTT CTCG------ CCTATACGAC GAGAATGTCC GCAA------ ---GGATGGA TGCTTTT---
SW349    GCATGGCCGT TCTTAGTTGG TGGAGTGATT TGTCAGGTTA ATTCCGGTAA CGAACGAGAC TGAAGCCTAT TAATTAGTTT CTCG------ CCTATACGAC GAGAATGTCC GCAA------ ---GGATGGA TGCTTTT---
IC88     GCATGGCCGT TCTTAGTTGG TGGAGTGATT TGTCAGGTTA ATTCCGGTAA CGAACGAGAC TGAAGCCTAT TAATTAGTTT CTCG------ CCTATACGAC GAGAATGTCC GCAA------ ---GGATGGA TGCTTTT---
SW350    GCATGGCCGT TCTTAGTTGG TGGAGTGATT TGTCAGGTTA ATTCCGGTAA CGAACGAGAC TGAAGCCTAT TAATTAGTTT CTCG------ CCTATACGAC GAGAATGTCC GCAA------ ---GGATGGA TGCTTTT---
SW345    GCATGGCCGT TCTTAGTTGG TGGAGTGATT TGTCAGGTTA ATTCCGGTAA CGAACGAGAC TGAAGCCTAT TAATTAGTTT CTCG------ CCTATACGAC GAGAATGTCC GCAA------ ---GGATGGA TGCTTTT---
Y012747  GCATGGCCGT TCTTAGTTGG TGGAGTGATT TGTCAGGTTA ATTCCGGTAA CGAACGAGAC TGAAGCCTAT TAATTAGTTT CTCG------ CCTATAAGAC GAGAATGTCC GCAA------ ---GGATGGA TGCTTTT---
Y012746  GCATGGCCGT TCTTAGTTGG TGGAGTGATT TGTCAGGTTA ATTCCGGTAA CGAACGAGAC TGAAGCCTAT TAATTAGTTT CTCG------ CCTATAAGAC GAGAATGTCC GCAA------ ---GGATGGA TGCTTTT---
Y012748  GCATGGCCGT TCTTAGTTGG TGGAGTGATT TGTCAGGTTA ATTCCGGTAA CGAACGAGAC TGAAGCCTAT TAATTAGTTT CTCG------ CCTATAAGAC GAGAATGTCC GCAA------ ---GGATGGA TGCTTTT---
N4       GCATGGCCGT TCTTAGTTGG TGGAGTGATT TGTCAGGTTA ATTCCGGTAA CGAACGAGAC TGAAGCCTAT TAATTAGTTT CTCG------ CCTATAAGAC GAGAATGTCC GTAT------ ---GGATGGA TGCTTTT---
N3       GCATGGCCGT TCTTAGTTGG TGGAGTGATT TGTCAGGTTA ATTCCGGTAA CGAACGAGAC TGAAGCCTAT TAATTAGTTT CTCG------ CCTATAAGAC GAGAATGTCC GTAT------ ---GGATGGA TGCTTTT---
N7       GCATGGCCGT TCTTAGTTGG TGGAGTGATT TGTCAGGTTA ATTCCGGTAA CGAACGAGAC TGAAGCCTAT TAATTAGTTT CTCG------ CCTATAAGAC GAGAATGTCC GTAT------ ---GGATGGA TGCTTTT---
N2       GCATGGCCGT TCTTAGTTGG TGGAGTGATT TGTCAGGTTA ATTCCGGTAA CGAACGAGAC TGAAGCCTAT TAATTAGTTT CTCG------ CCTATAAGAC GAGAATGTCC GTAT------ ---GGATGGA TGCTTTT---
V10      GCATGGCCGT TCTTAGTTGG TGGAGTGATT TGTCAGGTTA ATTCCGGTAA CGAACGAGAC TGAAGCCTAT TAATTAGTTT CTCG------ CCTATAAGAC GAGAATGTCC GTAT------ ---GGATGGA TGCTTTT---
V3       GCATGGCCGT TCTTAGTTGG TGGAGTGATT TGTCAGGTTA ATTCCGGTAA CGAACGAGAC TGAAGCCTAT TAATTAGTTT CTCG------ CCTATAAGAC GAGAATGTCC GTAT------ ---GGATGGA TGCTTTT---
N8       GCATGGCCGT TCTTAGTTGG TGGAGTGATT TGTCAGGTTA ATTCCGGTAA CGAACGAGAC TGAAGCCTAT TAATTAGTTT CTCG------ CCTATACGAC GAGAATGTCC GTAT------ ---GGATGGA TGCTTTT---
N666249  GCATGGCCGT TCTTAGTTGG TGGAGTGATT TGTCAGGTTA ATTCCGGTAA CGAACGAGAC TGAAGCCTAT TAATTAGTTT CTCG------ CCTATACGAC GAGAATGTCC GTAT------ ---GGATGGA TGCTTTC---
N666248  GCATGGCCGT TCTTAGTTGG TGGAGTGATT TGTCAGGTTA ATTCCGGTAA CGAACGAGAC TGAAGCCTAT TAATTAGTTT CTCG------ CCTATACGAC GAGAATGTCC GTAT------ ---GGATGGA TGCTTTC---
C329316  GCATGGCCGT TCTTAGTTGG TGGAGTGATT TGTCAGGTTA ATTCCGGTAA CGAACGAGAC TGAAGCCTAT TAATTAGTTT CTCG------ CCTATACGAC GAGAATGTCC GTAT------ ---GGATGGA TGCGTTC---
C329308  GCATGGCCGT TCTTAGTTGG TGGAGTGATT TGTCAGGTTA ATTCCGGTAA CGAACGAGAC TGAAGCCTAT TAATTAGTTT CTCG------ CCTATACGAC GAGAATGTCC GTAT------ ---GGATGGA TGCTCTC---
V14      GCATGGCCGT TCTTAGTTGG TGGAGTGATT TGTCAGGTTA ATTCCGGTAA CGAACGAGAC TGAAGCCTAT TAATTAGTTT CTCG------ CCTATAAGAC GAGAATGTCC GTAT------ ---GGATGGA TGCTTTC---
V7       GCATGGCCGT TCTTAGTTGG TGGAGTGATT TGTCAGGTTA ATTCCGGTAA CGAACGAGAC TGAAGCCTAT TAATTAGTTT CTCG------ CCTATACGAC GAGAATGTCC GTAT------ ---GGATGGA TGCTCTC---
V5       GCATGGCCGT TCTTAGTTGG TGGAGTGATT TGTCAGGTTA ATTCCGGTAA CGAACGAGAC TGAAGCCTAT TAATTAGTTT CTCG------ CCTATAAGAC GAGAATGTCC GCAT------ ---GGATGGA TGCTTTC---
N6       GCATGGCCGT TCTTAGTTGG TGGAGTGATT TGTCAGGTTA ATTCCGGTAA CGAACGAGAC TGAAGCCTAT TAATTAGTTT CTCG------ CCTATACGAC GAGAATGTCC GTAT------ ---GGATGGA TGCTTTC---
N1       GCATGGCCGT TCTTAGTTGG TGGAGTGATT TGTCAGGTTA ATTCCGGTAA CGAACGAGAC TGAAGCCTAT TAATTAGTTT CTCG------ CCTATAAGAC GAGAATGTCC GCAT------ ---GGATGGA TGCATTT---
N5       GCATGGCCGT TCTTAGTTGG TGGAGTGATT TGTCAGGTTA ATTCCGGTAA CGAACGAGAC TGAAGCCTAT TAATTAGTTT CTCG------ CCTATACGAC GAGAATGTCC GTAT------ ---GGATGGA TGCATTC---
V9       GCATGGCCGT TCTTAGTTGG TGGAGTGATT TGTCAGGTTA ATTCCGGTAA CGAACGAGAC TGAAGCCTAT TAATTAGTTT CTCG------ CCTATACGAC GAGAATGTCC GCAT------ ---GGATGGA TGCTTTT---
C329310  GCATGGCCGT TCTTAGTTGG TGGAGTGATT TGTCAGGTTA ATTCCGGTAA CGAACGAGAC TGAAGCCTAT TAATTAGTTT CTCG------ CCTATAAGAC GAGAATGTCC GCAA------ ---GGATGGA TGCTCTC---
SW340    GCATGGCCGT TCTTAGTTGG TGGAGTGATT TGTCAGGTTA ATTCCGGTAA CGAACGAGAC TGAAGCCTAT TAATTAGTTT CTCG------ CCTATAAGAC GAGAATGTCC GTAT------ ---GGATGGA TGCTTTC---
SW339    GCATGGCCGT TCTTAGTTGG TGGAGTGATT TGTCAGGTTA ATTCCGGTAA CGAACGAGAC TGAAGCCTAT TAATTAGTTT CTCG------ CCTATAAGAC GAGAATGTCC GTAT------ ---GGATGGA TGCTTTT---
C329314  GCATGGCCGT TCTTAGTTGG TGGAGTGATT TGTCAGGTTA ATTCCGGTAA CGAACGAGAC TGAAGCCTAT TAATTAGTTT CTCG------ CCTATAAGAC GAGAATGTCC GTAT------ ---GGATGGA TGCTTTT---
SW308    GCATGGCCGT TCTTAGTTGG TGGAGTGATT TGTCAGGTTA ATTCCGGTAA CGAACGAGAC TGAAGCCTAT TAATTAGTTT CTCG------ CCTATAAGAC GAGAATGTCC GTAT------ ---GGATGGA TGCTTTT---
IC85     GCATGGCCGT TCTTAGTTGG TGGAGTGATT TGTCAGGTTA ATTCCGGTAA CGAACGAGAC TGAAGCCTAT TAATTAGTTT CTCG------ CCTATACGAC GAGAATGTCC GCAA------ ---GGATGGA TGCTTTT---
SW318    GCATGGCCGT TCTTAGTTGG TGGAGTGATT TGTCAGGTTA ATTCCGGTAA CGAACGAGAC TGAAGCCTAT TAATTAGTTT CTCG------ CCTATACGAC GAGAATGTCC GCAA------ ---GGATGGA TGCTTTT---
SW330    GCATGGCCGT TCTTAGTTGG TGGAGTGATT TGTCAGGTTA ATTCCGGTAA CGAACGAGAC TGAAGCCTAT TAATTAGTTT CTCG------ CCTATACGAC GAGAATGTCC GCAA------ ---GGATGGA TGCTTTT---
SW322    GCATGGCCGT TCTTAGTTGG TGGAGTGATT TGTCAGGTTA ATTCCGGTAA CGAACGAGAC TGAAGCCTAT TAATTAGTTT CTCG------ CCTATACGAC GAGAATGTCC GCAA------ ---GGATGGA TGCTTTT---
SW324    GCATGGCCGT TCTTAGTTGG TGGAGTGATT TGTCAGGTTA ATTCCGGTAA CGAACGAGAC TGAAGCCTAT TAATTAGTTT CTCG------ CCTATACGAC GAGAATGTCC GCAA------ ---GGATGGA TGCTTTT---
SW305    GCATGGCCGT TCTTAGTTGG TGGAGTGATT TGTCAGGTTA ATTCCGGTAA CGAACGAGAC TGAAGCCTAT TAATTAGTTT CTCG------ CCTATACGAC GAGAATGTCC GCAA------ ---GGATGGA TGCTTTT---
SW315    GCATGGCCGT TCTTAGTTGG TGGAGTGATT TGTCAGGTTA ATTCCGGTAA CGAACGAGAC TGAAGCCTAT TAATTAGTTT CTCG------ CCTATACGAC GAGAATGTCC GCAT------ ---GGATGGA TGCTTTT---
SW320    GCATGGCCGT TCTTAGTTGG TGGAGTGATT TGTCAGGTTA ATTCCGGTAA CGAACGAGAC TGAAGCCTAT TAATTAGTTT CTCG------ CCTATACGAC GAGAATGTCC GCAA------ ---GGATGGA TGCTTTT---
SW321    GCATGGCCGT TCTTAGTTGG TGGAGTGATT TGTCAGGTTA ATTCCGGTAA CGAACGAGAC TGAAGCCTAT TAATTAGTTT CTCG------ CCTATACGAC GAGAATGTCC GTAT------ ---GGATGGA TGCTTTT---
SW341    GCATGGCCGT TCTTAGTTGG TGGAGTGATT TGTCAGGTTA ATTCCGGTAA CGAACGAGAC TGAAGCCTAT TAATTAGTTT CTCG------ CCTATAAGAC GAGAATGTCC GCAA------ ---GGATGGA TGCCTTT---
IC94     GCATGGCCGT TCTTAGTTGG TGGAGTGATT TGTCAGGTTA ATTCCGGTAA CGAACGAGAC TGAAGCCTAT TAATTAGTTT CTCG------ CCTATAAGAC GAGAATGTCC GCAA------ ---GGATGGA TGCTTTT---
IC92     GCATGGCCGT TCTTAGTTGG TGGAGTGATT TGTCAGGTTA ATTCCGGTAA CGAACGAGAC TGAAGCCTAT TAATTAGTTT CTCG------ CCTATAAGAC GAGAATGTCC GCAA------ ---GGATGGA TGCTTTT---
IC86     GCATGGCCGT TCTTAGTTGG TGGAGTGATT TGTCAGGTTA ATTCCGGTAA CGAACGAGAC TGAAGCCTAT TAATTAGTTT CTCG------ CCTATACGAC GAGAATGTCC GCAA------ ---GGATGGA TGCTTTT---
SW306    GCATGGCCGT TCTTAGTTGG TGGAGTGATT TGTCAGGTTA ATTCCGGTAA CGAACGAGAC TGAAGCCTAT TAATTAGTTT CTCG------ CCTATACGAC GAGAATGTCC GCAA------ ---GGATGGA TGCTTTT---
SW316    GCATGGCCGT TCTTAGTTGG TGGAGTGATT TGTCAGGTTA ATTCCGGTAA CGAACGAGAC TGAAGCCTAT TAATTAGTTT CTCG------ CCTATACGAC GAGAATGTCC GCAA------ ---GGATGGA TGCTTTT---
IC90     GCATGGCCGT TCTTAGTTGG TGGAGTGATT TGTCAGGTTA ATTCCGGTAA CGAACGAGAC TGAAGCCTAT TAATTAGTTT CTCG------ CCTATACGAC GAGAATGTCC GCAA------ ---GGATGGA TGCTTTT---
SW313    GCATGGCCGT TCTTAGTTGG TGGAGTGATT TGTCAGGTTA ATTCCGGTAA CGAACGAGAC TGAAGCCTAT TAATTAGTTT CTCG------ CCTATAAGAC GAGAATGTCC GCAA------ ---GGATGGA TGCTTTT---
SW317    GCATGGCCGT TCTTAGTTGG TGGAGTGATT TGTCAGGTTA ATTCCGGTAA CGAACGAGAC TGAAGCCTAT TAATTAGTTT CTCG------ CCTATACGAC GAGAATGTCC GCAA------ ---GGATGGA TGCTTTT---
SW325    GCATGGCCGT TCTTAGTTGG TGGAGTGATT TGTCAGGTTA ATTCCGGTAA CGAACGAGAC TGAAGCCTAT TAATTAGTTT CTCG------ CCTATACGAC GAGAATGTCC GCAA------ ---GGATGGA TGCTTTC---
C329318  GCATGGCCGT TCTTAGTTGG TGGAGTGATT TGTCAGGTTA ATTCCGGTAA CGAACGAGAC TGAAGCCTAT TAATTAGTTT CTCG------ CCTATAAGAC GAGAATGTCC GCAA------ ---GGATGGA TGCTTTT---
N666251  GCATGGCCGT TCTTAGTTGG TGGAGTGATT TGTCAGGTTA ATTCCGGTAA CGAACGAGAC TGAAGCCTAT TAATTAGTTT CTCG------ CCTATACGAC GAGAATGTCC GCAA------ ---GGATGGA TGCGTTT---
IC87     GCATGGCCGT TCTTAGTTGG TGGAGTGATT TGTCAGGTTA ATTCCGGTAA CGAACGAGAC TGAAGCCTAT TAATTAGTTT CTCG------ CCTATACGAC GAGAATGTCC GCAA------ ---GGATGGA TGCGTTT---
SW319    GCATGGCCGT TCTTAGTTGG TGGAGTGATT TGTCAGGTTA ATTCCGGTAA CGAACGAGAC TGAAGCCTAT TAATTAGTTT CTCG------ CCTATACGAC GAGAATGTCC GCAA------ ---GGATGGA TGCGTCC---
SW307    GCATGGCCGT TCTTAGTTGG TGGAGTGATT TGTCAGGTTA ATTCCGGTAA CGAACGAGAC TGAAGCCTAT TAATTAGTTT CTCG------ CCTATACGAC GAGAATGTCC GCAA------ ---GGATGGA TGCGTCC---
```

```
ISW360        GCATGGCCGT TCTTAGTTGG TGGAGTGATT TGTCAGGTTA ATTCCGGTAA CGAACGAGAC TGAAGCCTAT TAATTAGTTT CCCG------ CCTATACGAC GGGAATGTCC GCAA------ ---GGATGGA TGCATTT---
ISW346        GCATGGCCGT TCTTAGTTGG TGGAGTGATT TGTCAGGTTA ATTCCGGTAA CGAACGAGAC TGAAGCCTAT TAATTAGTTT CTCG------ CCTATACGAC GAGAATGTCC GCAA------ ---GGATGGA TGCATTT---
'IC82         GCATGGCCGT TCTTAGTTGG TGGAGTGATT TGTCAGGTTA ATTCCGGTAA CGAACGAGAC TGAAGCCTAT TAATTAGTTT CCCG------ CCTATACGAC GGGAATGTCC GCAA------ ---GGATGGA TGCGTAT---
ISW328        GCATGGCCGT TCTTAGTTGG TGGAGTGATT TGTCAGGTTA ATTCCGGTAA CGAACGAGAC TGAAGCCTAT TAATTAGTTT CCCG------ CCTATAAGAC GGGAATGTCC GCAA------ ---GGATGGA TGCGTAA---
              ....|....| ....|....| ....|....| ....|....| ....|....| ....|....| ....|....| ....|....| ....|....| ....|....| ....|....| ....|....| ....|....| ....|....|
                   425        435        445        455        465        475        485        495        505        515        525        535        545        555
'IC84         GCATGGCCGT TCTTAGTTGG TGGAGTGATT TGTCAGGTTA ATTCCGGTAA CGAACGAGAC TGAAGCCTAT TAATTAGTTT CTCG------ CCTATACGAC GAGAATGTCC GCAA------ ---GGATGGA TGCGTTT---
ISW329        GCATGGCCGT TCTTAGTTGG TGGAGTGATT TGTCAGGTTA ATTCCGGTAA CGAACGAGAC TGAAGCCTAT TAATTAGTTT CTCG------ CCTATAAGAC GAGAATGTCC GCAA------ ---GGATGGA TGCTTTT---
'IC83         GCATGGCCGT TCTTAGTTGG TGGAGTGATT TGTCAGGTTA ATTCCGGTAA CGAACGAGAC TGAAGCCTAT TAATTAGTTT CTCG------ CCTATACGAC GAGAATGTCC GCAA------ ---GGATGGA TGCTTTC---
ISW304        GCATGGCCGT TCTTAGTTGG TGGAGTGATT TGTCAGGTTA ATTCCGGTAA CGAACGAGAC TGAAGCCTAT TAATTAGTTT CCTG------ CCTATAAGAC AGGAATG-CC GCAA------ ---GG-TGGG TGCATTT---
IR025406      GCATGGCCGT TCTTAGTTGG TGGAGTGATT TGTCAGGTTA ATTCCGGTAA CGAACGAGAC TGAAGCCTAT TAATTAGTTT CCTG------ CCTATAAGAC AGGAATG-CC GCAA------ ---GG-TGGG TGCATTT---
'R686358      GCATGGCCGT TCTTAGTTGG TGGAGTGATT TGTCAGGTTA ATTCCGGTAA CGAACGAGAC TGAAACCTAT TAATTAGTTT CACG------ CCTATACGAC GTGAATGTCC GCAA------ ---GGATGGA TGCTTTT---
'N666253      GCATGGCCGT TCTTAGTTGG TGGAGTGATT TGTCAGGTTA ATTCCGGTAA CGAACGAGAC TGAAGCCTAT TAATTAGTTT CTCA------ CCTATACGAT GAGAATGTCC GCAA------ ---GGATGGA TGCGTAA---
'IC89         GCATGGCCGT TCTTAGTTGG TGGAGTGATT TGTCAGGTTA ATTCCGGTAA CGAACGAGAC TGAAGCCTAT TAATTAGTTT CTCA------ CCTATACGAT GAGAATGTCC GCAA------ ---GGATGGA TGCGTAA---
'R686363      GCATGGCCGT TCTTAGTTGG TGGAGTGATT TGTCAGGTTA ATTCCGGTAA CGAACGAGAC TGAAGCCTAT TAATTAGTTT CTCA------ CCTATAAGAT GAGAACGTCA GCAA------ ---TGACGGA TG--TTT---
'IC93         GCATGGCCGT TCTTAGTTGG TGGAGTGATT TGTCAGGTTA ATTCCGGTAA CGAACGAGAC TGAAGCCTAT TAATTAGTTT TCCA------ TCTATACGGT GGAAATGTTC GCAA------ ---GAACAGA TGCATTC---
ISW332        GCATGGCCGT TCTTAGTTGG TGGAGTGATT TGTCAGGTTA ATTCCGGTAA CGAACGAGAC TGAAGCCTAT TAATTAGTTT TCCA------ TCTATACGGT GGAAATGTTC GCAA------ ---GAACGGA TGCATTC---
'R686361      GCATGGCCGT TCTTAGTTGG TGGAGTGATT TGTCAGGTTA ATTCCGGTAA CGAACGAGAC TGAAACCTAT TAATTAGTTG CTTA------ CCTATACGAT AAGCATGT-- AGCA------ ---ATACAGC TGCTC-----
.F149909      GCATGGCCGT TCTTAGTTGG TGGAGTGATT TGTCAGGTTA ATTCCGGTAA CGAACGAGAC TGAAACCTAT TAATTAGTTT TTCA------ TCCATGAGGT GAAAATGT-- AGTA------ ---ATACAAC TGCTATCA--
'IC91         GCATGGCCGT TCTTAGTTGG TGGAGTGATT TGTCAGGTTA ATTCCGGTAA CGAACGAGAC TGAAACCTAT TAATTAGTTC CAGT------ TCTATATGAA TTGGATG-CC GCAA------ ---GG-TAGA TGTATCT---
)Q286371      GCATGGCCGT TCTTAGTTGG TGGAGTAATT TGTCAGGTTA ATTCCGGTAA CGAACGAGAC TGAAACCTAT TAATTAGTTA ACTA------ TCTATAAGGT AGTTATGT-- AGCA------ ---ATACAGA TGTTTTC---
.F149915      GCATGGCCGT TCTTAGTTCG TGGACTGATT TGTCTGGTTA ATTCCGATAA CGAACGAGAC TTGAACTTAT TAATTAGTTG GGCAAGAATC ATTTTCGGAT GATTTTGCTT ACCTCCGTAA GGTGGACTGG TGGAATTAAA
.B445018      GCATGGCCGT TCTTAGTTCG TGGAGTGATT TGTCTGGTTA ATTCCGATAA CGAACGAGAC TTGAACTTGC TAATTAGTTG GACAATAAAT TCCTTCGGGG GTTTATGTTT ATTCTCTCCT --TGGAGAGT TACACGCAAA
.F149910      GCATGGCCGT TCTTAGTTGG TGGAGTGATT TGTCGGGTTA ATTCCGGTAA CGAACGAGAC TGAAACCTAT TAATTAATTA ATCA------ TCCATGAGGT GATTATATTC AGCAA----- --TGGATACG TGATCAC---
.F149907      GCATGGCCGT TCTTAGTTGG TGGTATGAAT TGTCAGGTTA ATTCCGGTAA CGAACGAGAC TGAAACCTAT TAATTAGTTC TGTG------ CCTATAAGAC ACAGGTGTT- AGCAA----- --TA-ACAGA TAGCGAT---
IX027294      GCATGGCCGT TCTTAGTTGG TGGAATGATT TGTCAGGTTA ATTCCGGTAA CGAACGAGAC TTAAACCTAT TAATTAGTTA CATTTGA--- TGTAAATAAA ATGTATATAT AGAAA----- ---TATATAA TTATTAGTAA
)Q286372      GCATGGCCGT TCTTAGTTGG TGGAATGATT TGTCAGGTTA ATTCCGGTAA CGAACGAGAC TTAAACCTAT TAATTAGTTG AAT------- ---ACCTAAA AGATATTCAT GTAGT----- ---AATACAA TTATTATTTT
.F149913      GCATGGCCGT TCTTAGTTGG TGGAGTGATT TGTCTGGTTA ATTCCGATAA CGAACGAGAC TTTATCTTAT TAATTGGTTT AGT------- --------AA GAGTTTACTT TTACT----- ---AAAATTA TATTTAT---
.Y769863      GCATGGCCGT TCTTAGTTCG TGGACTGGTT TGTCTGGTTA ATTCCGATAA CGAACGAGAC TGAAACCTAT TAATTAGTAT AT-------- CTTTGACAGG GGATATCATG CG-------- ----AAAGCA TAT-TAGGAA
.F149908      GCATGGCCGT TCTTAGTTCG TGGAGTAATT CGTCAGGTTA ATTCCGGTAA CGAACGAGAC TGAAACCTAT TTATTAGTAT TTT------- CTCTGTTAGG GAAAATTGTA TGTGT----- ---AAAAACA TACACAAGAA
:onsensus     ********** ******** * ***  *   *  *** ***** ****** *** ********** *  * * *   * ***  *                                                          *

              ....|....| ....|....| ....|....| ....|....| ....|....| ....|....| ....|....| ....|....| ....|....| ....|....| ....|....| ....|....| ....|....| ....|....|
                   565        575        585        595        605        615        625        635        645        655        665        675        685        695
I64142        ----GTACC- ACTTCTTAAA GGGACACATT TCAATTGTCC TATT----TT AATTGTAGT- ---TATCTAA TTTCGGTT-- --AGACCTCT TT-------- TAACGTGGGA AAAAGAAAAA GGAAGCATTC AGCAATAACA
'R686356      ----GTACC- ACTTCTTAAA GGGACACATT TCAATTGTCC TATT----TT AATTGTAGT- ---TATCTAA TTTCGGTT-- --AGAGCTCT TT-------- TAACGTGGGA AAAAGAAAAA GGAAGCATTC AGCAATAACA
IR025411      ----GTACC- ACTTCTTAAA GGGACACATT TCAATTGTCC TATT----TT AATTGTAGT- ----ATCTAA CTTCGGTT-- --AGAACTCT TT-------- TAACGTGGGA AAAAGAAAAA GGAAGCATTC AGCAATAACA
)Q286373      ----GTACC- ACTTCTTAAA GGGACACATT TCAATTGTCT TGTT----TT AATCGTAGT- ----ATCTGA TTTCGGTC-- --AGATCTCC TT-------- TAAAATGAGA AAAAGAAAAA GGAAGCATTC AGCAATAACA
.F149906      ----GTACC- ACTTCTTAAA GGGACACATT TCAATTGTCT CATT----TT AATCGATGT- ----CTCTGG CTCCGGTC-- --AGAACTCT TT-------- TAACGTGAGA AAAAGAAAAA GGAAGCATTC AGCAATAACA
ISW344        ----GCATC- ACTTCTTAAA GGGACTAATA TCAATTGTTT CTCG----GT TGATCAGTG- ---CGACCA- TCTCGAT--- --GGTACCTT TT-------- CAATCGGAAA AAAAGATAAT GGAAGCATTC AGCAATAACA
ISW343        ----GCATC- ACTTCTTAAA GGGACTAATA TCAATTGTTT CTCG----GT TGATCAGTG- ---CGACCA- TCTCGAT--- --GGTACCTT TT-------- CAATCGGAAA AAAAGATAAT GGAAGCATTC AGCAATAACA
'N666250      ----GCATC- ACTTCTTAAA GGGACTAATA TCAATTGTTT CTCG----GT TGATCAGTG- ---CGACCA- TCTCGAT--- --GGTACCTT TT-------- CAATCGGAAA AAAAGATAAT GGAAGCATTC AGCAATAACA
.C329307      ----GCATC- ACTTCTTAAA GGGACTAATA TCAATTGTTT CTCG----GT TGATCAGTG- ---CGACCA- TCTCGAT--- --GGTACCTT TT-------- CAATCGGAAA AAAAGATAAT GGAAGCATTC AGCAATAACA
ISW312        ----GCATC- ACTTCTTAAA GGGACTAATA TCAATTGTTT CTCG----GT TGATCAGTG- ---CGACCA- TCTCGAT--- --GGTACCTT TT-------- CAATCGGAAA AAAAGATAAT GGAAGCATTC AGCAATAACA
.C329309      ----GCATC- ACTTCTTAAA GGGACTAATA TCAATTGTTT CTCG----GT TGATCAGTG- ---CGACCA- TCTCGAT--- --GGTACCTT TT-------- CAATCGGAAA AAAAGATAAT GGAAGCATTC AGCAATAACA
ISW327        ----GCATC- ACTTCTTAAA GGGACTAATA TCAATTGTTT CTCG----GT TGATCAGTG- ---CGACCG- TTTCGAC--- --GGTACCTT TT-------- CAATCGGAAA AAAAGATAAT GGAAGCATTC AGCAATAACA
ISW326        ----GCATC- ACTTCTTAAA GGGACTAATA TCAATTGTTT CTCG----GT TGATTAGTG- ---CGACCG- TTTCGAC--- --GGTACCTT TT-------- CAATCGGAAA AAAAGATAAT GGAAGCATTC AGCAATAACA
ISW331        ----GCATC- ACTTCTTAAA GGGACTAATA TCAATTGTTT CTCG----GT TGATTAGTG- ---CGACCG- TCTCGAT--- --GGTACCTT TT-------- CAATCGGAAA AAAAGATAAT GGAAGCATTC AGCAATAACA
ISW349        ----GCATC- ACTTCTTAAA GGGACTAATA TCAATTGTTT CTCG----GT TGATCAGTG- ---CGACCA- TTTCGAT--- --GGTACCTT TT-------- CAATCGGAAA AAAAGATAAT GGAAGCATTC AGCAATAACA
'IC88         ----GCATC- ACTTCTTAAA GGGACTAATA TCAATTGTTT CTCG----GT TGATCAGTG- ---CGACCA- TCTCGAT--- --GGTACCTT TT-------- CAATCGGAAA AAAAGATAAT GGAAGCATTC AGCAATAACA
ISW350        ----GCATC- ACTTCTTAAA GGGACTAATA TCAATTGTTT CTCG----GT TGATCAGTG- ---CGACCA- TCTCGAT--- --GGTACCTT TT-------- CAATCGGAAA AAAAGATAAT GGAAGCATTC AGCAATAACA
ISW345        ----GCATC- ACTTCTTAAA GGGACTAATA TCAATTGTTT CTCG----GT TGATCAGTG- ---CGACCA- TCTCGAT--- --GGTACCTT TT-------- CAATCGGAAA AAAAGATAAT GGAAGCATTC AGCAATAACA
IY012747      ----GCATC- ACTTCTTAAA GGGACTAATA TCAATTGTTT CTCG----GT TGATCAGTG- ---CGACCA- TCTCGAC--- --GGTACCTT TT-------- CAATCGGAAA AAAAGATAAT GGAAGCATTC AGCAATAACA
IY012746      ----GCATC- ACTTCTTAAA GGGACTAATA TCAATTGTTT CTCG----GT TGATCAGTG- ---CTACCA- TCTCGAC--- --GGTACCTT TT-------- CAATCGGAAA AAAAGATAAT GGAAGCATTC AGCAATAACA
IY012748      ----GCATC- ACTTCTTAAA GGGACTAATA TCAATTGTTT CTCG----GT TGATCAGTG- ---CGACCA- TCTCGAT--- --GGTACCTT TT-------- CAATCGGAAA AAAAGATAAT GGAAGCATTC AGCAATAACA
.N4           ----GCATC- ACTTCTTAAA GGGACTAATA TCAATTGTTT CTCG----GT TGATCAGTG- ---CGACCA- TCTCGAT--- --GGTACCTT TT-------- CAATCGGAAA AAAAGATAAT GGAAGCATTC AGCAATAACA
.N3           ----GCATC- ACTTCTTAAA GGGACTAATA TCAATTGTTT CTCG----GT TGATCAGTG- ---CGACCA- TCTCGAT--- --GGTACCTT TT-------- CAATCGGAAA AAAAGATAAT GGAAGCATTC AGCAATAACA
.N7           ----GCATC- ACTTCTTAAA GGGACTAATA TCAATTGTTT CTCG----GT TGATCAGTG- ---CGACCA- TCTCGAT--- --GGTACCTT TT-------- CAATCGGAAA AAAAGATAAT GGAAGCATTC AGCAATAACA
.N2           ----GCATC- ACTTCTTAAA GGGACTAATA TCAATTGTTT CTCG----GT TGATCAGTG- ---CGACCA- TCTCGAT--- --GGTACCTT TT-------- CAATCGGAAA AAAAGATAAT GGAAGCATTC AGCAATAACA
.V10          ----GCATC- ACTTCTTAAA GGGACTAATA TCAATTGTTT CTCG----GT TGATCAGTG- ---CGACCA- TCTCGAT--- --GGTACCTT TT-------- CAATCGGAAA AAAAGATAAT GGAAGCATTC AGCAATAACA
.V3           ----GCATC- ACTTCTTAAA GGGACTAATA TCAATTGTTT CTCG----GT TGATCAGTG- ---CGACCA- TCTCGAT--- --GGTACCTT TT-------- CAATCGGAAA AAAAGATAAT GGAAGCATTC AGCAATAACA
.N8           ----GCATC- ACTTCTTAAA GGGACTAATA TCAATTGTTT CTCG----GT TGATCAGTG- ---CGACCA- TCTCGAT--- --GGTACCTT TT-------- CAATCGGAAA AAAAGATAAT GGAAGCATTC AGCAATAACA
'N666249      ----GCATC- ACTTCTTAAA GGGACTAATA TCAATTGTTT CCCG----GT TGATCAGTG- ---CGACCA- TCTCGAT--- --GGTACCTC TT-------- CAATCGGAAA AAAAGATAAT GGAAGCATTC AGCAATAACA
```

```
C329316   ----GCATC- ACTTCTTAAA GGGACTAATA TCAATTGTTT CCAG----GT TGATAAGTG- ---CGACCA- TCTCGAT--- --GGTACCTT TT-------- CAATCGGAAA AAAAGATAAT GGAAGCATTC AGCAATAACA
C329308   ----GCATC- ACTTCTTAAA GGGACTAATA TCAATTGTTT CCAG----GT TGATCAGTG- ---CGACCA- TCTCGAT--- --GGTACCTT TT-------- CAATCGGAAA AAAAGATAAT GGAAGCATTC AGCAATAACA
V14       ----GCATC- ACTTCTTAAA GGGACTAATA TCAATTGTTT CTCG----GT TGATCAGTG- ---CGACCA- TCTCGAT--- --GGTACCTT TT-------- CAATCGGAAA AAAAGATAAT GGAAGCATTC AGCAATAACA
V7        ----GCATC- ACTTCTTAAA GGGACTAATA TCAATTGTTT CTCG----GT TGATCAGTG- ---CGACCA- TCTCGAT--- --GGTACCTC TT-------- CAATCGGAAA AAAAGATAAT GGAAGCATTC AGCAATAACA
V5        ----GCATC- ACTTCTTAAA GGGACTAATA TCAATTGTTT CTCG----GT TGATCAGTG- ---CGACCA- TCTCGAT--- --GGTACCTT TT-------- CAATCGGAAA AAAAGATAAT GGAAGCATTC AGCAATAACA
N6        ----GCATC- ACTTCTTAAA GGGACTAATA TCAATTGTTT CTCG----GT TGATCAGTG- ---CGACCA- TCTCGAT--- --GGTACCTT TT-------- CAATCGGAAA AAAAGATAAT GGAAGCATTC AGCAATAACA
N1        ----GCATC- ACTTCTTAAA GGGACTAATA TCAATTGTTT CTCG----GT TGATCAGTG- ---CGACCA- TCTCGAT--- --AGTACCTT TT-------- CAATCGGAAA AAAAGATAAT GGAAGCATTC AGCAATAACA
N5        ----GCATC- ACTTCTTAAA GGGACTAATA TCAATTGTTT CTCG----GT TGATCAGTG- ---CGACCA- TCTCGAT--- --AGTACCTT TT-------- CAATCGGAAA AAAAGATAAT GGAAGCATTC AGCAATAACA
V9        ----GCATC- ACTTCTTAAA GGGACTAATA TCAATTGTTT CTCG----GT TGATCAGTG- ---CGACCA- TCTCGAT--- --GGTACCTT TT-------- CAATCGGAAA AAAAGATAAT GGAAGCATTC AGCAATAACA
C329310   ----GCATC- ACTTCTTAAA GGGACTAATA TCAATTGTTT CTCG----GT TGATCAGTG- ---CGACCA- TCTCGAT--- --GGTACCTT TT-------- CAATCGGAAA AAAAGATAAT GGAAGCATTC AGCAATAACA
SW340     ----GCATC- ACTTCTTAAA GGGACTAATA TCAATTGTTT CTCG----GT TGATCAGTG- ---CGACCA- TCTCGAT--- --GGTACCTC TT-------- CAATCGGAAA AAAAGATAAT GGAAGCATTC AGCAATAACA
SW339     ----GCATC- ACTTCTTAAA GGGACTAATA TCAATTGTTT CTCG----GT TGATCAGTG- ---CGACCA- TCTCGAT--- --GGTACCTT TT-------- CAATCGGAAA AAAAGATAAT GGAAGCATTC AGCAATAACA
C329314   ----GCATC- ACTTCTTAAA GGGACTAATA TCAATTGTTT CTCG----GT TGATCAGTG- ---CGACCA- TCTCGAT--- --GGTACCTT TT-------- CAATCGGAAA AAAAGATAAT GGAAGCATTC AGCAATAACA
SW308     ----GCATC- ACTTCTTAAA GGGACTAATA TCAATTGTTT CTCG----GT TGATAAGTG- ---CGACCA- TCTCGAT--- --GGTACCTT TT-------- CAATCGGAAA AAAAGATAAT GGAAGCATTC AGCAATAACA
IC85      ----GCATC- ACTTCTTAAA GGGACTAATA TCAATTGTTT CTCG----GT TGATCAGTG- ---TGACCA- TCTCGAT--- --GGTACCTT TT-------- CAATCGGAAA AAAAGATAAT GGAAGCATTC AGCAATAACA
SW318     ----GCATC- ACTTCTTAAA GGGACTAATA TCAATTGTTT CTCG----GT TGATCAGTG- ---CGACCA- TCTCGAT--- --GGTACCTT TT-------- CAATCGGAAA AAAAGATAAT GGAAGCATTC AGCAATAACA
SW330     ----GCATC- ACTTCTTAAA GGGACTAATA TCAATTGTTT CTCG----GT TGATTAGTG- ---CGACCA- TCTCGAT--- --GGTACCTC TT-------- CAATCGGAAA AAAAGATAAT GGAAGCATTC AGCAATAACA
SW322     ----GCATC- ACTTCTTAAA GGGACTAATA TCAATTGTTT CTCG----GT TGATTAGTG- ---CGACCA- TCTCGAT--- --GGTACCTC TT-------- CAATCGGAAA AAAAGATAAT GGAAGCATTC AGCAATAACA
SW324     ----GCATC- ACTTCTTAAA GGGACTAATA TCAATTGTTT CTCG----GT TGATTAGTG- ---CGACCA- TCTCGAT--- --GGTACCTT TT-------- CAATCGGAAA AAAAGATAAT GGAAGCATTC AGCAATAACA
SW305     ----GCATC- ACTTCTTAAA GGGACTAATA TCAATTGTTT CTCG----GT TGATCAGTG- ---CGACCA- TCTCGAT--- --GGTACCTT TT-------- CAATCGGAAA AAAAGATAAT GGAAGCATTC AGCAATAACA
SW315     ----GCATC- ACTTCTTAAA GGGACTAATA TCAATTGTTT CTCG----GT TGATCAGTG- ---CGACCA- TCTCGAT--- --GGTACCTT TT-------- CAATCGGAAA AAAAGATAAT GGAAGCATTC AGCAATAACA
SW320     ----GCATC- ACTTCTTAAA GGGACTAATA TCAATTGTTT CTCG----GT TGATCAGTG- ---CGACCA- TCTCGAT--- --GGTACCTT TT-------- CAATCGGAAA AAAAGATAAT GGAAGCATTC AGCAATAACA
SW321     ----GCATC- ACTTCTTAAA GGGACTAATA TCAATTGTTT CTCG----GT TGATCAGTG- ---CGACCA- TCTCGAT--- --GGTACCTT TT-------- CAATCGGAAA AAAAGATAAT GGAAGCATTC AGCAATAACA
SW341     ----GCATC- ACTTCTTAAA GGGACTAATA TCAATTGTTT CTCG----GT TGATCAGTG- ---CGACCA- TCTTGAT--- --GGTACCTT TT-------- CAATCGGAAA AAAAGATAAT GGAAGCATTC AGCAATAACA
IC94      ----GCATC- ACTTCTTAAA GGGACTAATA TCAATTGTTT CTCG----GT TGATCAGTG- ---CGACCA- TCTCGAT--- --GGTACCTC TT-------- CAATCGGAAA AAAAGATAAT GGAAGCATTC AGCAATAACA
IC92      ----GCATC- ACTTCTTAAA GGGACTAATA TCAATTGTTT CTCG----GT TGATCAGTG- ---CGACCA- TCTCGAT--- --GGTACCTC TT-------- CAATCGGAAA AAAAGATAAT GGAAGCATTC AGCAATAACA
IC86      ----GCATC- ACTTCTTAAA GGGACTAATA TCAATTGTTT CTCG----GT TGATTAGTG- ---CGACCA- TCTCGAT--- --GGTACCTC TT-------- CAATCGGAAA AAAAGATAAT GGAAGCATTC AGCAATAACA
SW306     ----GCATC- ACTTCTTAAA GGGACTAATA TCAATTGTTT CTCG----GT TGATTAGTG- ---CGACCA- TCTCGAT--- --GGTACCTC TT-------- CAATCGGAAA AAAAGATAAT GGAAGCATTC AGCAATAACA
SW316     ----GCATC- ACTTCTTAAA GGGACTAATA TCAATTGTTT CTCG----GT TGATTAGTG- ---CGACCA- TCTCGAT--- --GGTACCTC TT-------- CAATCGGAAA AAAAGATAAT GGAAGCATTC AGCAATAACA
IC90      ----GCATC- ACTTCTTAAA GGGACTAATA TCAATTGTTT CTCG----GT TGATTAGTG- ---CGACCA- TCTCGAT--- --GGTACCTC TT-------- CAATCGGAAA AAAAGATAAT GGAAGCATTC AGCAATAACA
SW313     ----GCATC- ACTTCTTAAA GGGACTAATA TCAATTGTTT CTCG----GT TGATCAGTG- ---CGACCA- TCTCGAT--- --GGTACCTC TT-------- CAATCGGAAA AAAAGATAAT GGAAGCATTC AGCAATAACA
SW317     ----GCATC- ACTTCTTAAA GGGACTAATA TCAATTGTTT CTCG----GT TGATCAGTG- ---CGACCA- TCTCGAT--- --GGTACCTT TT-------- CAATCGGAAA AAAAGATAAT GGAAGCATTC AGCAATAACA
SW325     ----GCATC- ACTTCTTAAA GGGACTAATA TCAATTGTTT CTCG----GT TGATCAGTG- ---CGACCA- TCTCGAT--- --GGTACCTT TT-------- CAATCGGAAA AAAAGATAAT GGAAGCATTC AGCAATAACA
C329318   ----GCATC- ACTTCTTAAA GGGACTAATA TCAATTGTTT CTCG----GT TGATTAGTG- ---CGACCA- TCTCGAT--- --GGTACCTT TT-------- CAATCGGAAA AAAAGATAAT GGAAGCATTC AGCAATAACA
N666251   ----GCATC- ACTTCTTAAA GGGACTAATA TCAATTGTTT CTCG----GT TGATCAGTG- ---CTACCG- TCTCGAC--- --GGTTCCTT TT-------- CAATCGGAAA AAAAGATAAT GGAAGCATTC AGCAATAACA
IC87      ----GCATC- ACTTCTTAAA GGGACTAATA TCAATTGTTT CTCG----GT TGATCAGTG- ---CTACCG- TCTCGAC--- --GGTTCCTT TT-------- CAATCGGAAA AAAAGATAAT GGAAGCATTC AGCAATAACA
SW319     ----GCATC- ACTTCTTAAA GGGACTAATA TCAATTGTTT CTCG----GT TGATCAGTG- ---TCACCG- TCTCGAC--- --GGTTCCTT TT-------- CAATCGGAAA AAAAGATAAT GGAAGCATTC AGCAATAACA
SW307     ----GCATC- ACTTCTTAAA GGGACTAATA TCAATTGTTT CTCG----GT TGATCAGTG- ---TCACCG- TCTCGAC--- --GGTTCCTT TT-------- CAATCGGAAA AAAAGATAAT GGAAGCATTC AGCAATAACA
N666252   ----GCATC- ACTTCTTAAA GGGACTAATA TCAATTGTTT CTCG----GT TGATCAGTG- ---CGACTG- TTTCGAC--- --AGTACCTT TT-------- CAATCGGAAA AAAAGATAAT GGAAGCATTC AGCAATAACA
SW360     ----GTATC- ACTTCTTAAA GGGACTAATA TCAATTGTTT CTCG----GT TAATTTGCG- ---TTGCTA- TTTCGAT--- --AGCACCTT TT-------- TAATCGGAAA AAAAGATAAT GGAAGCATTC AGCAATAACA
SW346     ----GTATC- ACTTCTTAAA GGGACTAATA TCAATTGTTT CTCG----GT TAATTTGCG- ---TCGCTA- TTTCGAT--- --AGCACCTT TT-------- TAATCGGAAA AAAAGATAAT GGAAGCATTC AGCAATAACA
IC82      ----GTATC- ACTTCTTAAA GGGACTAATA TCAATTGTTT CTCG----GT TAATTTGCG- ---TTGCTA- TTTCGAT--- --AGTGCCTT TT-------- TAATCGGAAA AAAAGATAAT GGAAGCATTC AGCAATAACA
SW328     ----GTATC- ACTTCTTAAA GGGACTAATA TCAATTGTTT CTCG----GT TAATTTGCG- ---TTGCTA- TTTCGAT--- --AGTGCCTT TT-------- TAATCGGAAA AAAAGATAAT GGAAGCATTC AGCAATAACA
IC84      ----GTATC- ACTTCTTAAA GGGACTAATA TCAATTGTTT CTCG----GT TGATTTGCG- ---TTGCTA- TTTCGAT--- --AGTGCCTT TT-------- TAATCGGAAA AAAAGATAAT GGAAGCATTC AGCAATAACA
SW329     ----GTATC- ACTTCTTAAA GGGACTAATA TCAATTGTTT CTCG----GT TGATTAGCG- ---TTACTA- TCTCGAT--- --AGTGCCTT TT-------- TAATCGGAAA AAAAGATAAT GGAAGCATTC AGCAATAACA
IC83      ----GTATC- ACTTCTTAAA GGGACTAATA TCAATTGTTT CTCG----GT TGATTAGTG- ---TTACTA- TTTCGAT--- --GGTACCTT TT-------- CAATCGGAAA AAAAGATAAT GGAAGCATTC AGCAATAACA
SW304     ----GCATC- ACTTCTTAAA GGGACTAATA TCAATTGTTT CTCG----GT TAATCTGTG- ---ATACCG- GTTCGCC--- --GGTGCCTC TT-------- TAATCGGAAA AAAAGATAAT GGAAGCATTC AGCAATAACA
R025406   ----GCATC- ACTTCTTAAA GGGACTAATA TCAATTGTTT CTCG----GT TAATCTGTG- ---ATACCG- GTTCGCC--- --GGTGCCTC TT-------- TAATCGGAAA AAAAGATAAT GGAAGCATTC AGCAATAACA
R686358   ----GCATC- ACTTCTTAAA GGGACTAATA TCAATTGTTT CTAG----GT TAATTTGTG- ---CCATAA- CTTCGGT--- --TATGCCTT TT-------- TAATCAGAAA AAAAGATAAT GGAAGCATTC AGCAATAACA
N666253   ----GTATC- ACTTCTTAAA GGGACTAATA TCAATTGTTT CACG----GT TAATAAGTG- ---AAGTCA- TTTCGAT--- --GACAACTT TT-------- TAATCGGAAA AAAAGATAAT GGAAGCATTC AGCAATAACA
IC89      ----GTATC- ACTTCTTAAA GGGACTAATA TCAATTGTTT CACG----GT TGATAAGTG- ---AAGTCA- TTTCGAT--- --GACAACTT TT-------- TAATCGGAAA AAAAGATAAT GGAAGCATTC AGCAATAACA
R686363   ----ACATC- ACTTCTTAAA GGGACTAATA TCAATTGTTT CTCG----GT TAATGTGTG- ---ATTTGAG CTTCGGC--- --TCAACCTA TT-------- TAATCGGAAA AAAAGATAAT GGAAGCATTC AGCAATAACA
IC93      ----GTATC- ACTTCTTAAA GGGACTAATA TCAATTGTTT CCAG----GT TAATTGGTG- ---TATTCA- CTTCGGT--- --GAGACCCT TT-------- TAATCGGAAA AAAAGATAAA GGAAGCATTC AGCAATAACA
SW332     ----GTATC- ACTTCTTAAA GGGACTAATA TCAATTGTTT CCAG----GT TAATTAGTG- ---TATTCA- CTTCGAT--- --GAGACCCT TT-------- TAATCGGAAA AAAAGATAAT GGAAGCATTC AGCAATAACA
R686361   ----GCAGC- ACTTCTTAAA GGGACACATT TCAATTGTCT CATT----CT ATTCTGTGT- ---CAGGTAC TTCGGTA--- --TCTACCTC TA-------- TAGAAGGAGA AAAAGAAAAA GGAAGCATTC AGCAATAACA
F149909   ----GTAGT- ACTTCTTAAA GGGACACATT TCAATTGTTC CACT----TT AATTGGATC- ---CGTACAC TCACGTG--- --TACGACCA TT-------- TAACGTGGAA AAAAGAAAAA GGAAGCATTC AGCAATAACA
IC91      ----ATATC- ACTACTTAAA GGGACACATT TCAATTGTTT TCCA----CT ACAAGTCTC- ---TTCTGGT TCCTACCA-- --GATATCTT GT-------- TGA-GGAAAA AAAAGAAAAA GGAAGCATTC AGCAATAACA
Q286371   ------ATC- ACTACTTAAA GGGACACATT TCAATTGTTT CC------AT TTAATAGTG- ---GAGGTTT TTCCAAA--- -----ACTTT TA-------- RAA--GGAAA AAAAGAAAAA GGAAGCGTTC AGCAATAACA
F149915   TTTCGCCTTT ACTTCTTAAA GGGACACATC TTTAATCTTT TCGGGT--GT TCATTGGTT- ---TCTTTGG GCTTTGCCCT TAGATGATAT GA-------- ATACCTGAAA AAAAGAAGAA GGAAGCGTCA AGCAATAACA
B445018   TGAAGCGTG- ACTTCTTAGA GGGACAAATC TTTATTCCTT TACGTC--GT TGGCACGCC- ---TCTTTCC TTTCGGGGTT AGAGTTTGCT AA-------- CGGTCGTAAA ACAAAAAGAA GGAAGCATCA AGCAATAACA
F149910   ------ACG- ATTACTTAAA GGGACACATT TCAATTGTTT TTCATT--AA TCGTGTGTT- ---AAAGTA- TTACTTT--- ---AACTGCT TT-------- TAATGGGAAA AAAAGAAAAA GGAAGCATTC AGCAATAACA
F149907   ------ATC- ACTTCTTAAA GGGACACATT TCAAATGTTC TTTGAT--TA TCAAAAGTT- ---GGA---- TTCTGTC--- ---CAACGTG AT-------- AAACGAAGAA AAAAGAAACA GGAAGCATTC AGCAATAACA
X027294   TT--AATAAT ACTACTTAAA GGGACAACTA TCAATCTCTT TTCTCTCTTT TAGATAATAA TTTGTGTTAT TT------GA ATAATAGAAA TTATGTAAAA GAGAGAAAAG AAAAGATAAA GGAAGCGTTA AGCAATAACA
```

```
.F149913  --------AT ACTATTTAAA AGGACAGAC- ----TCTCAT T-------TT GAATCAAT-- ------TTAT TT-------A ATTAAATTGA TT-------- ----TAAAGA AAGAGG---A GGAAGCATGA AGCAATAACA
.Y769863  TT--A----A ATTTCTTAGA GGGACAACTT TTAGATAGTC CTCCCAGCGG TATATGA--- ---GTACTAA CC-------- GTACTTTTTA TT-------- TCTGGAGGGA TTAATGAAAG GGAAGCGTTC AGCAATAACA
.F149908  AC--AT--TG ATTTCTTAAA GGGACACGTT TTGATAGATT ACTCTCGAGT TAGCTACT-- ---ACAGAAA AT-------- GTGGTACTAA TC-------- CAGGAGTCGT TTAAAGAAAG GGAAGCGTTC AGCAATAACA
Consensus            * *  *** *  ****                                                                                       *        ****** *   **********

          ....|....| ....|....| ....|....| ....|....| ....|....| ....|....| ....|....| ....|....| ....|....| ....|....| ....|....| ....|....| ....|....| ....|....|
                705        715        725        735        745        755        765        775        785        795        805        815        825        835
:64142    GGTCTGTGAT GCCCTTAGAC ATCTTGGGCC GCACSCSCGC TACAATGGAG TTACTAGAGA GTATTTTATC ATTT--ACAC CTTATTTATT A-------GG CTTTGT-CTA ATAATTAAGG ATAGTA---- -AGTGGTGTA
R686356   GGTCTGTGAT GCCCTTAGAC ATCTTGGGCC GCACGCGCGC TACAATGGAG TTACTAGAGA GCATTTTATC ATTT--ACAC CTTATTTATT A-------GG CTTTGT-CTA ATAATTAAGG ATAGTG---- -AGTGGTGTA
R025411   GGTCTGTGAT GCCCTTAGAC ATCTTGGGCC GCACGCGCGC TACAATGGAG TTACTAGAGA GCAATTTATT ATTT--ACGC CTTTTTTATT A-------GG CTTTGT-CTA ATAGGAGGGG ATAGTA---- -AATGATGTA
Q286373   GGTCTGTGAT GCCCTTAGAC ATCTTGGGCC GCACGCGCGC TACAATGGAG TTACTAGAGA GCATTTTATT ATTC--ATGC CTTATTTATT A-------GG CTCTGT-CTA ATGGATAGGG AGAGTG---- -AGTAGTGTA
.F149906  GGTCTGTGAT GCCCTTAGAC ATCTTGGGCC GCACGCGCGC TACAATGGAG TTACTAGAGA GTATTTTATC ATCC--AAGC CTTATATTGT A-------GA CTTTGT-TTA TAATGTAGGG ATATTG---- -GGTGATGTA
ISW344    GGTCTGTGAT GCCCTTAGAC ATCTTGGGCT GCACGCGCGC TACAATGGAG ATACTAGCGA GTATTT-GTC GGGG--ATGC TCGGTTTATC --------GG CTCTG--TCG GTAGATCGAG ATTTTC---- -CCCGGTGTA
ISW343    GGTCTGTGAT GCCCTTAGAC ATCTTGGGCT GCACGCGCGC TACAATGGAG ATACTAGCGA GTATTT-GTC GGGG--ATGC TCGGTTTATC --------GG CTCTG--TCG GTAGATCGAG ATTTTC---- -CCCGGTGTA
'N666250  GGTCTGTGAT GCCCTTAGAC ATCTTGGGCT GCACGCGCGC TACAATGGAG ATACTAGCGA GTATTT-GTC GGGG--ATGC TCGGTTTATC --------GG CTCTG--TCG GTAGATCGAG ATTTTC---- -CCCGGTGTA
.C329307  GGTCTGTGAT GCCCTTAGAC ATCTTGGGCT GCACGCGCGC TACAATGGAG ATACTAGCGA GTATTT-GTC GGGG--ATGC TCGGTTTATC --------GG CTCTG--TCG GTAGATCGAG ATTTTC---- -CCCGGTGTA
ISW312    GGTCTGTGAT GCCCTTAGAC ATCTTGGGCT GCACGCGCGC TACAATGGAG ATACTAGCGA GTATTT-GTC GGGG--ATGC TCGGTTTATC --------GG CTCTG--TCG GTAGATCGAG ATTTTC---- -TCCGGTGTA
.C329309  GGTCTGTGAT GCCCTTAGAC ATCTTGGGCT GCACGCGCGC TACAATGGAG ATACTAGCGA GTATTT-GTC GGGG--ATGC TCGGTTTATC --------GG CTCTG--TCG GTAGATCGAG ATTTTC---- -CCCGATGTA
ISW327    GGTCTGTGAT GCCCTTAGAC ATCTTGGGCT GCACGCGCGC TACAATGGAG ATACTAGCGA GTATTT-GTC GGGG--ATGC TCGGTTTATC --------GG CTCTG--TCG GTAGATCGAG ATTTTC---- -CCCGGTGTA
ISW326    GGTCTGTGAT GCCCTTAGAC ATCTTGGGCT GCACGCGCGC TACAATGGAG ATACTAGCGA GTATTT-GTC GGGG--ATGC TCGGTTTATC --------GG CTCTG--TCG GTAGATCGAG ATTTTC---- -CCCGGTGTA
ISW331    GGTCTGTGAT GCCCTTAGAC ATCTTGGGCT GCACGCGCGC TACAATGGAG ATACTAGCGA GTATTT-GTC GGGG--ATGC TCGGTTTATC --------GG CTCTG--TCG GTAGATCGAG ATTTGC---- -CACGATGTA
ISW349    GGTCTGTGAT GCCCTTAGAC ATCTTGGGCT GCACGCGCGC TACAATGGAG ATACTAGCGA GTATTT-GTC GGGG--ATGC TCGGTTTATC --------GG CTCTG--TCG GTAGATCGAG ATTTCC---- -CCCGGTGTA
'IC88     GGTCTGTGAT GCCCTTAGAC ATCTTGGGCT GCACGCGCGC TACAATGGAG ATACTAGCGA GTATTT-GTC GGGG--ATGC TCGGTTTATC --------GG CTCTG--TCG GTAGATCGAG ATTTTC---- -CCCGGTGTA
ISW350    GGTCTGTGAT GCCCTTAGAC ATCTTGGGCT GCACGCGCGC TACAATGGAG ATACTAGCGA GTATTT-GTC GGGG--ATGC TCGGTTTATC --------GG CTCTG--TCG GTGGATCGAG ATTTTC---- -CCCGGTGTA
ISW345    GGTCTGTGAT GCCCTTAGAC ATCTTGGGCT GCACGCGCGC TACAATGGAG ATACTAGCGA GTATTT-GTC GGGG--ATGC TCGGTTTATC --------GG CTCTG--TCG GTGAAGTGAG ATTTTC---- -CCCGGTGTA
:Y012747  GGTCTGTGAT GCCCTTAGAC ATCTTGGGCT GCACGCGCGC TACAATGGAG ATACTAGCGA GTATTT-GTC GGGG--ATGC CTATTCCATC --------GG CTCTG--TCG GTGGATCGAG ATTTTC---- -CCCGGTGTA
:Y012746  GGTCTGTGAT GCCCTTAGAC ATCTTGGGCT GCACGCGCGC TACAATGGAG ATACTAGCGA GTATTT-GTC GGGG--ATGC TCGGTTTATC --------GG CTCTGG-TCG GCGGGTCGAG ATTTTC---- -CCCGGTGTA
:Y012748  GGTCTGTGAT GCCCTTAGAC ATCTTGGGCT GCACGCGCGC TACAATGGAG ATACTAGCGA GTATTT-GTC GGGG--ATGC TCGGTTTATC --------GG CTCTG--TCG GTGGATCGAG ATTTTC---- -CCCGGTGTA
.N4       GGTCTGTGAT GCCCTTAGAC ATCTTGGGCT GCACGCGCGC TACAATGGAG ATACTAGCGA GTATTT-GTC GGGA--ATGC TCGGTTTATC --------GG CTCTG--TCG GTGAGTCGAG ATTTTC---- -TCCGATGTA
.N3       GGTCTGTGAT GCCCTTAGAC ATCTTGGGCT GCACGCGCGC TACAATGGAG ATACTAGCGA GTATTT-GTC GGGA--ATGC TCGGTTTATC --------GG CTCTG--TCG GTGAGTCGAG ATTTTC---- -TCCGATGTA
.N7       GGTCTGTGAT GCCCTTAGAC ATCTTGGGCT GCACGCGCGC TACAATGGAG ATACTAGCGA GTATTT-GTC GGGA--ATGC TCGGTTTATC --------GG CTCTG--TCG GTGAGTCGAG ATTTTC---- -TCCGATGTA
.N2       GGTCTGTGAT GCCCTTAGAC ATCTTGGGCT GCACGCGCGC TACAATGGAG ATACTAGCGA GTATTT-GTC GGGA--ATGC TCGGTTTATC --------GG CTCTG--TCG GTGAGTCGAG ATTTTC---- -TCCGATGTA
.V10      GGTCTGTGAT GCCCTTAGAC ATCTTGGGCT GCACGCGCGC TACAATGGAG ATACTAGCGA GTATTT-GTC GGGA--ATGC TCGGTTTATC --------GG CTCTG--TCG GTGAGTCGAG ATTTTC---- -TCCGGTGTA
.V3       GGTCTGTGAT GCCCTTAGAC ATCTTGGGCT GCACGCGCGC TACAATGGAG ATACTAGCGA GTATTT-GTC GGGA--ATGC TCGGTTTATC --------GG CTCTG--TCG GTGAGTCGAG ATTTTC---- -TCCGGTGTA
.N8       GGTCTGTGAT GCCCTTAGAC ATCTTGGGCT GCACGCGCGC TACAATGGAG ATACTAGCGA GTATTT-GTC GGGA--ATGC TCGGTTTATC --------GG CTCTG--TCG GTGAGTCGAG ATTTTC---- -TCCGGTGTA
'N666249  GGTCTGTGAT GCCCTTAGAC ATCTTGGGCT GCACGCGCGC TACAATGGAG ATACTAGCGA GTATTT-GTC GGGA--ATGC TCGGTTTATC --------GG CTCTG--TCG GTGAATCGAG ATTTTC---- -TCCGGTGTA
'N666248  GGTCTGTGAT GCCCTTAGAC ATCTTGGGCT GCACGCGCGC TACAATGGAG ATACTAGCGA GTATTT-GTC GGGA--ATGC TCGGTTTATC --------GG CTCTG--TCG GTGAATCGAG ATTTTC---- -TCCGGTGTA
.C329316  GGTCTGTGAT GCCCTTAGAC ATCTTGGGCT GCACGCGCGC TACAATGGAG ATACTAGCGA GTATTT-GTC GGGA--ATGC TCGGTTTATC --------GG CTCTG--TCG GTGAATCGAG ATTTTC---- -TCCGGTGTA
.C329308  GGTCTGTGAT GCCCTTAGAC ATCTTGGGCT GCACGCGCGC TACAATGGAG ATACTAGCGA GTATTT-GTC GGGA--ATGC TCGGTTTATC --------GG CTCTG--TCG GTGAATCGAG ATTTTT---- -TCCGGTGTA
.V14      GGTCTGTGAT GCCCTTAGAC ATCTTGGGCT GCACGCGCGC TACAATGGAG ATACTAGCGA GTATTT-GTC GGGA--ATGC TCGGTTTATC --------GG CTCTG--TCG GTGAGTCGAG ATTTTC---- -TCCGGTGTA
.V7       GGTCTGTGAT GCCCTTAGAC ATCTTGGGCT GCACGCGCGC TACAATGGAG ATACTAGCGA GTATTT-GTC GGGA--ATGC TCGGTTTATC --------GG CTCTG--TCG GTGAGTCGAG ATTTTC---- -TCCGGTGTA
.V5       GGTCTGTGAT GCCCTTAGAC ATCTTGGGCT GCACGCGCGC TACAATGGAG ATACTAGCGA GTATTT-GTC GGGA--ATGC TCGGTTTATC --------GG CTCTG--TCG GTGAGTCGAG ATTTTC---- -TCCGGTGTA
.N6       GGTCTGTGAT GCCCTTAGAC ATCTTGGGCT GCACGCGCGC TACAATGGAG ATACTAGCGA GTATTT-GTC GGGA--ATGC TCGGTTTATC --------GG CTCTG--TCG GTGAGTCGAG ATTTTC---- -TCCGGTGTA
.N1       GGTCTGTGAT GCCCTTAGAC ATCTTGGGCT GCACGCGCGC TACAATGGAG ATACTAGCGA GTATTT-GTC GGGA--ATGC TCGGTTTATC --------GG CTCTG--TCA CTGAGTCGAG ATGTTC---- -TCCGGTGTA
.N5       GGTCTGTGAT GCCCTTAGAC ATCTTGGGCT GCACGCGCGC TACAATGGAG ATACTAGCGA GTATTT-GTC GGGA--ATGC TCGGTTTAGC --------GG CTCTG--TCA GTGAGTCGAG ATGTTC---- -TCCGGTGTA
.V9       GGTCTGTGAT GCCCTTAGAC ATCTTGGGCT GCACGCGCGC TACAATGGAG ATACTAGCGA GTATTT-GTC GGGA--ATGC TCGGTTTATC --------GG CTCTG--TCG GTGAGTCGAG ATTTTC---- -TCCGGTGTA
.C329310  GGTCTGTGAT GCCCTTAGAC ATCTTGGGCT GCACGCGCGC TACAATGGAG ATACTAGCGA GTATTT-GTC GGGA--ATGC TCGGTTTATC --------GG CTCTG--TCG GTGAATCGAG ATTTTC---- -TCCGGTGTA
ISW340    GGTCTGTGAT GCCCTTAGAC ATCTTGGGCT GCACGCGCGC TACAATGGAG ATACTAGCGA GTATTT-GTC GGGA--ATGC TCGGTTTATC --------GG CTCTG--TCG GTGAATCGAG ATTTTC---- -TCCGGTGTA
ISW339    GGTCTGTGAT GCCCTTAGAC ATCTTGGGCT GCACGCGCGC TACAATGGAG ATACTAGCGA GTATTT-GTC GGGG--ATGC TCGGTTTATC --------GG CTCTG--TCG GTAAATCGAG ATTTTC---- -TCCGATGTA
.C329314  GGTCTGTGAT GCCCTTAGAC ATCTTGGGCT GCACGCGCGC TACAATGGAG ATACTAGCGA GTATTT-GTC GGGG--ATGC TCGGTTTATC --------GG CTCTG--TCG GTAAATCGAG ATTTTC---- -TCCGATGTA
ISW308    GGTCTGTGAT GCCCTTAGAC ATCTTGGGCT GCACGCGCGC TACAATGGAG ATACTAGCGA GTATTT-GTC GGGG--ATGC TCGGTTTATC --------GG CTCTG--TCG GTAGATCGAG ATTTTC---- -CCCGATGTA
'IC85     GGTCTGTGAT GCCCTTAGAC ATCTTGGGCT GCACGCGCGC TACAATGGAG ATACTAGCGA GTATTT-GTC GGGG--ATGC TCAGTTTAGC --------GG CTCTG--TCG GTGGATCGAG ATTTTC---- -CACGGTGTA
ISW318    GGTCTGTGAT GCCCTTAGAC ATCTTGGGCT GCACGCGCGC TACAATGGAG ATACTAGCGA GTATTT-GTC GGGG--ATGC TCGATTCAGC --------GG CTCTG--TCC GTAGATCGAG ATTTTC---- -CCCGGTGTA
ISW330    GGTCTGTGAT GCCCTTAGAC ATCTTGGGCT GCACGCGCGC TACAATGGAG ATACTAGCGA GTATTT-GTC GGGA--ATGC TCGATTCAGC --------GG CTCTG--TCG GTGGATCGAG ATTTGC---- -TTCGATGTA
ISW322    GGTCTGTGAT GCCCTTAGAC ATCTTGGGCT GCACGCGCGC TACAATGGAG ATACTAGCGA GTATTT-GTC GGGA--ATGC TCGATTCATC --------GG CTCTG--TCG GTGGATCGAG ATTTGC---- -TTCGATGTA
ISW324    GGTCTGTGAT GCCCTTAGAC ATCTTGGGCT GCACGCGCGC TACAATGGAG ATACTAGCGA GTATTT-GTC GGGA--ATGC TCGATTCAGC --------GG CTCTG--TCG GTGGATCGAG ATTTGC---- -TCCGATGTA
ISW305    GGTCTGTGAT GCCCTTAGAC ATCTTGGGCT GCACGCGCGC TACAATGGAG ATACTAGCGA GTATTT-GTC GGGA--ATGC TCGGTTCAGC --------GG CTCTG--TCG GTGGAGCGAG ATTTGC---- -CCCGATGTA
ISW315    GGTCTGTGAT GCCCTTAGAC ATCTTGGGCT GCACGCGCGC TACAATGGAG ATACTAGCGA GTATTT-GTC GGGA--ATGC TCGATTCAGC --------GG CTCTG--TCG GTGAATCGAG ATTTTC---- -TCCGATGTA
ISW320    GGTCTGTGAT GCCCTTAGAC ATCTTGGGCT GCACGCGCGC TACAATGGAG ATACTAGCGA GTATTT-GTC GGGA--ATGC TCGGTTTTGC --------GG CTCTG--TCG GTAGAGCGAG ATTTTC---- -CCCGATGTA
ISW321    GGTCTGTGAT GCCCTTAGAC ATCTTGGGCT GCACGCGCGC TACAATGGAG ATACTAGCGA GTATTT-GTC GGGA--ATGC TCGGTTTATC --------GG CTCTG--TCG GTAGATCGAG ATTTTC---- -TCCGATGTA
ISW341    GGTCTGTGAT GCCCTTAGAC ATCTTGGGCT GCACGCGCGC TACAATGGAG ATACTAGCGA GTATTT-GTC GGGG--ATGC CCGGTATATC --------GG CTCTG--TCG GTAGAATGAG ATTTGC---- -GCCGGTGTA
'IC94     GGTCTGTGAT GCCCTTAGAC ATCTTGGGCT GCACGCGCGC TACAATGGAG ATACTAGCGA GTATTT-GTC GGGG--ATGC TCGATTCATC --------GG CTTTG--TCG GTGAGATGAG ATTTGT---- -TTCGATGTA
'IC92     GGTCTGTGAT GCCCTTAGAC ATCTTGGGCT GCACGCGCGC TACAATGGAG ATACTAGCGA GTATTT-GTC GGGG--ATGC TCGATTCATC --------GG CTCTG--TCG GTGAGGTGAG ATTTGT---- -TTCGCTGTA
```

```
ISW306     GGTCTGTGAT GCCCTTAGAC ATCTTGGGCT GCACGCGCGC TACAATGGAG ATACTAGCGA GTATTT-GTC GGGA--CTGC TCGATTCATC --------GG CTCTG--TCG GTGGGTTGAG ATTTGT---- -TTCGATGTA
ISW316     GGTCTGTGAT GCCCTTAGAC ATCTTGGGCT GCACGCGCGC TACAATGGAG ATACTAGCGA GTATTT-GTC GGGA--CTGC TCGATTCAGC --------GG CTCTG--TCG GTGGGTTGAG ATTTGT---- -TTCGATGTA
VIC90      GGTCTGTGAT GCCCTTAGAC ATCTTGGGCT GCACGCGCGC TACAATGGAG ATACTAGCGA GTATTT-GTC GGGA--CTGC TCGATTCATC --------GG CTCTG--TCG GTGGGTTGAG ATTTGT---- -TTCGATGTA
ISW313     GGTCTGTGAT GCCCTTAGAC ATCTTGGGCT GCACGCGCGC TACAATGGAG ATACTAGCGA GTATTT-GTC GAGG--CTGC TCGATTCATC --------GG CTCTG--TCG GTGGATTGAG ATTTGT---- -CTCGATGTA
ISW317     GGTCTGTGAT GCCCTTAGAC ATCTTGGGCT GCACGCGCGC TACAATGGAG ATACTAGCGA GTATTT-GTC GGGG--ATGC TCGATTCAGC --------GG CTCTG--TCG GTCGGGTGAG ATTGTT---- -CTCGATGTA
ISW325     GGTCTGTGAT GCCCTTAGAC ATCTTGGGCT GCACGCGCGC TACAATGGAG ATACTAGCGA GTATTT-GTC GGGA--ATGC TCGATTCAGC --------GG CTCTG--TCG GTGGAGCGAG ATTTTT---- -TTCGATGTA
LC329318   GGTCTGTGAT GCCCTTAGAC ATCTTGGGCT GCACGCGCGC TACAATGGAG ATACTAGCGA GTATTT-GTC GGGG--ATGC CCGTTTCATC --------GG CTCTG--TCG GTGAGTCGAG ATGTTC---- -TCCGGTGTA
FN666251   GGTCTGTGAT GCCCTTAGAC ATCTTGGGCT GCACGCGCGC TACAATGGAG ATACTAGCGA GTATTT-GTC GGGG--TTGC TCGATTCGTT --------GG CTTCGG-TCA GCGGGTTGAG ATGTTC---- -TCCG-TGTA
VIC87      GGTCTGTGAT GCCCTTAGAC ATCTTGGGCT GCACGCGCGC TACAATGGAG ATACTAGCGA GTATTT-GTC GGGG--TTGC TCGATTCATC --------GG CT-CGG-TCA TCGAGTTGAG ATGTTC---- -TCCGGTGTA
ISW319     GGTCTGTGAT GCCCTTAGAC ATCTTGGGCT GCACGCGCGC TACAATGGAG ATACTAGCGA GTATTT-GTC GAGG--TTGC TCGGGTCGCT --------GG CT-CGG-TCA GCGGTTCGAG ATGTTC---- -TTCGATGTA
ISW307     GGTCTGTGAT GCCCTTAGAC ATCTTGGGCT GCACGCGCGC TACAATGGAG ATACTAGCGA GTATTT-GTC GAGG--TTGC TCGGGTCGCT --------GG CT-CTG-TCA GCGGTTCGAG ATGTTC---- -TTCGATGTA
FN666252   GGTCTGTGAT GCCCTTAGAC ATCTTGGGCT GCACGCGCGC TACAATGGAG ATACTAGCGA GTATTT-GTC GGGG--TTGC CTATTCCATT --------GG CTTCGG-TCA GTGGAGTGGG ATGTTC---- -TTCGATGTT
ISW360     GGTCTGTGAT GCCCTTAGAC ATCTTGGGCT GCACGCGCGC TACAATGGAG ATACTAGCGA GTATTT-GCC GGGG--AAGC CCATTTCTGT --------GG CTTTG--TCA CCGAGGTGGG ATGTTC---- -AACGGTGTA
ISW346     GGTCTGTGAT GCCCTTAGAC ATCTTGGGCT GCACGCGCGC TACAATGGAG ATACTAGCGA GTATTT-GCC GGGG--AAGC CCACTTCTGT --------GG CTTTG--TCA CCGAGGTGGG ATGTTC---- -AACGGTGTA
VIC82      GGTCTGTGAT GCCCTTAGAC ATCTTGGGCT GCACGCGCGC TACAATGGAG ATACTAGCGA GTATTT-GCC GGGG--AAGC CCATTTCTGT --------GG CTATG--TCA CCGAGGTGGG ATGTTC---- -AACGGTGTA
ISW328     GGTCTGTGAT GCCCTTAGAC ATCTTGGGCT GCACGCGCGC TACAATGGAG ATACTAGCGA GTATTT-ACC GGGG--AAGC CCATTTCTCT --------GG CTATG--TCA GCGGGGTGAG ATGTTC---- -AACGGTGTA
VIC84      GGTCTGTGAT GCCCTTAGAC ATCTTGGGCT GCACGCGCGC TACAATGGAG ATACTAGCGA GTATTT-GTC GGGG--ATGC TCATTTCTGT --------GG CTCTG--TCA GCGAGGTGAG ATGTTC---- -AACGGTGTA
ISW329     GGTCTGTGAT GCCCTTAGAC ATCTTGGGCT GCACGCGCGC TACAATGGAG ATACTAGCGA GTATTT-GCC GGGG--AAGC TCAGTTCAGC --------GG CTCTG--TCA GCAGAGTGAG ATGTTC---- -AACGGTGTA
VIC83      GGTCTGTGAT GCCCTTAGAC ATCTTGGGCT GCACGCGCGC TACAATGGAG ATACTAGCGA GTATTT-GTC GGGG--ATGC CCGTTTCTCC --------GG CTCTG--TCG CCGGAGTGAG ATGTTC---- -ACCGGTGTA
ISW304     GGTCTGTGAT GCCCTTAGAC ATCTTGGGCT GCACGCGCGC TACAATGGAG ATACTAGCGA GCATTC-CGT CGCG--ATGC TTATTTAATT --------GG CTTTG--TCA GTTAGGTGAG ATTATT---- -GCGACAATA
MR025406   GGTCTGTGAT GCCCTTAGAC ATCTTGGGCT GCACGCGCGC TACAATGGAG ATACTAGCGA GCATTC-CGT CGCG--ATGC TTATTTAATT --------GG CTTTG--TCA GTTAGGTGAG ATTATT---- -GCGACAATA
JR686358   GGTCTGTGAT GCCCTTAGAC ATCTTGGGCT GCACGCGCGC TACAATGGAG ATACTAGCGA GTATTT-GGC GAGA--GAGC TTATTTATTC --------GA CTCTG--TTG GATGGGTAAG ATTTCT---- -CTCGTTGTA
FN666253   GGTCTGTGAT GCCCTTAGAC ATCTTGGGCT GCACGCGCGC TACAATGGAG ATACTAGCGA GTATTT-ATC GTTG--AAGC CCATTTTACT --------GA CTTTG--TTA GTAGAGTGGG ATGTTT---- -GACGGTGTA
VIC89      GGTCTGTGAT GCCCTTAGAC ATCTTGGGCT GCACGCGCGC TACAATGGAG ATACTAGCGA GTATTT-ATC GATG--AAGC CCGTATTACT --------GA CTTTG--TTA GTAGAGTGGA ATGTTT---- -GACTGTGTA
JR686363   GGTCTGTGAT GCCCTTAGAC ATCTTGGGCT GCACGCGCGC TACAATGGAG ATACTAGCGA GTATTT-TTC GTAG--ATGC TTGTTTTACT --------GA TTCTG--TCA GTAAGATGAG ATTATC---- -TGCGTCTTA
VIC93      GGTCTGTGAT GCCCTTAGAC ATCTTGGGCT GCACGCGCGC TACAATGGAG ATACTAGCGA GTATTT-AAC GAAG--CTGC CTGTATATCC --------AC TTCGG---TG GCTATATAGG ATTAGC---- -GACGTTGTA
ISW332     GGTCTGTGAT GCCCTTAGAC ATCTTGGGCT GCACGCGCGC TACAATGGAG ATACTAGCGA GTATTT-ATC GAAG--CTGC CTGTATATCC --------AC TTCTG---TG GCTATATAGG ATTATC---- -GACGTTGTA
JR686361   GGTCTGTGAT GCCCTTAGAC ATCTTGGGCC GCACGCGCGC TACAATGGAG TTACTAGAGA GTATTTAGGT A-GT--AGGT CCCTTTTTGA --------GG CTTCGG-TTT CAGGGAGGAT CAACCT---- -ATTATCATA
AF149909   GGTCTGTGAT GCCCTTAGAC ATCTTGGGCT GCACGCGCGC TACAATGGAG TTACTAGAGA GTATTTTGGT AAGG--ACGT TTCTTTTCAA --------GG CTTCGG-TCT TGGATTGAAA ATTGTT---- -CTTATCGTA
VIC91      GGTCTGTGAT GCCCTTAGAC ATCTTGGGCT GCACGCGCGC TACAATGGAG TTACTAGAGA GTATATTATT AGATCTATGC TTGATTAATA ---------G ATTCG--TCT ATTTATTGAG AAGTTA---- -GCTAATATA
DQ286371   GGTCTGTGAT GCCCTTAGAC ATCTTGGGCT GCACGCGCGC TACAATGGAG TTACTAGAGA GTAATT--TT GTAAC-GCTA GCATTTAAGT A-------AG CT-TGC-TTA CTTAGGTGTG ATGTTT---- -TAC--TATA
AF149915   GGTCTGTGAT GCCCTTAGAC ATCTTGGGCT GCACGCGTGC TACAATGAAA GTACTAACGA GTCCC---CG TTGTCGGCAA TGGGTTGAGT C-------TT TCGGGACGTT ---GATTCAT TGCTGG---- -CAACATTTT
AB445018   GGTCTGTGAT GCCCTTAGAC ATCTTGGGCT GCACGCGTGC TACAATGAAA GTACTAGAGA GTTCAA-GTG TTTTTTACTG TCTTTTGGGG G-------GT TTCTGACTTT TCTGAGATAG TGAAAG---- -ATGCGTTCA
AF149910   GGTCTGTGAT GCCCTTAGAC ATCTTGGGCT GCACGCGCGC TACAATGAAG TTACTAGAGA GTAATTAAT- TGAGATA-TC GTATAAGATG A-------GA CTTTGTTTCA TTTTGTATGT A-ATAT---- -CACAATGTA
AF149907   GGTCTGTGAT GCCCTTAGAC ATCTTGGGCT GCACGCGCGC TACAATGAAG ATAGTAGAGA GTAATATATA TATGACAGTT TCGTAATCAT A-------TA TTGTTTACAA TGTTGGTTAT AGAAAG---- -TGTCATAAA
MX027294   GGTCTGTGAT GCCCTTAGAC ATCTTGGGCT GCACGCGCGC TACAATGAAA ATACTAAAGA GTAGTACTTT A-ATAATGGA AATTTTTATT -------ATT TTATAACATT ATAGAATAAT AAAAAT--GA CATTATTTTA
DQ286372   GGTCTGTGAT GCCCTTAGAC ATCTTGGGCT GCACGCGCGC TACAATGGAA ATACTAAAGA GTAGTAATTT AGATATTGGA AATAATAAAT -------ATT T-ATACTTGT ATAAATATTT ATTATTA-GA AAATATTTTA
AF149913   GGTCTGTGAT GCCCTTAGAC ATCTTGGGCT GCACGCGTGC TACAATGGAA TTTATAGAGA GTAAT--TTG AAGTAAAAGG AAGGTTTAAT -------ATA T--TTATATA TTAGATATGG AATTTT---- ---ATTTTTA
AY769863   GGTCTGTGAT GCCCTTAGAC ATCTTGGGCC GCACGCGCGC TACAATGGAA GAAGAAAAGA GTT-TAGTCA AGGAATTATT TCATTTGGTT CTGCCAAGTG GAGTGATTTC TAAAAAATGG ACACAAGAAT TTTTGACTAG
AF149908   GGTCTGTGAT GCCCTTAGAC ATCTTGGGCC GCACGCGCGC TACAATGGAA AAGGAAAAGA GTA-CAGTTG GAAGATTATT TTTATTGG-- ---------G ACGTTGTTTC AATAAGAATG ATACTA---- -TTTGGCTGA
Consensus  ********** ********** *********  **** *  ** ******* *       *  ** *
```

```
                     ....|....| ....|....| ....
                        845        855
K64142               CCGAGATTGA AATAGTTAAG GAAA
JR686356             CCGAGATTGA AATAGTTAAG GAAA
MR025411             CCGAGATTGA AATAGTTAAG GAAA
DQ286373             CCGAGATTGA AATAGTTAAG GAAA
AF149906             CCTAAATTGA AATAGTTAAG GAAA
ISW344               CCGAGACTGA AATAGTTTAG GAAA
ISW343               CCGAGACTGA AATAGTTTAG GAAA
FN666250             CCGAGACTGA AATAGTTTAG GAAA
LC329307             CCGAGACTGA AATAGTTTAG GAAA
ISW312               CCGAGACTGA AATAGTTTAG GAAA
LC329309             CCGAGACTGA AATAGTTTAG GAAA
ISW327               CCGAGACTGA AATAGTTTAG GAAA
ISW326               CCGAGACTGA AATAGTTTAG GAAA
ISW331               CCGAGACTGA AATAGTTTAG GAAA
ISW349               CCGAGACTGA AATAGTTTAG GAAA
VIC88                CCGAGACTGA AATAGTTTAG GAAA
ISW350               CCGAGACTGA AATAGTTTAG GAAA
ISW345               CCGAGACTGA AATAGTTTAG GAAA
KY012747             CCGAGACTGA AATAGTTTAG GAAA
KY012746             CCGAGACTGA AATAGTTTAG GAAA
KY012748             CCGAGACTGA AATAGTTTAG GAAA
LN4                  CCGAGACTGA AATAGTTTAG GAAA
LN3                  CCGAGACTGA AATAGTTTAG GAAA
LN7                  CCGAGACTGA AATAGTTTAG GAAA
                     ....|....| ....|....| ....
                        845        855
LN2                  CCGAGACTGA AATAGTTTAG GAAA
LV10                 CCGAGACTGA AATAGTTTAG GAAA
LV3                  CCGAGACTGA AATAGTTTAG GAAA
LN8                  CCGAGACTGA AATAGTTTAG GAAA
FN666249             CCGAGACTGA AATAGTTTAG GAAA
FN666248             CCGAGACTGA AATAGTTTAG GAAA
LC329316             CCGAGACTGA AATAGTTTAG GAAA
LC329308             CCGAGACTGA AATAGTTTAG GAAA
LV14                 CCGAGACTGA AATAGTTTAG GAAA
LV7                  CCGAGACTGA AATAGTTTAG GAAA
LV5                  CCGAGACTGA AATAGTTTAG GAAA
LN6                  CCGAGACTGA AATAGTTTAG GAAA
LN1                  CCGAGACTGA AATAGTTTAG GAAA
LN5                  CCGAGACTGA AATAGTTTAG GAAA
```

```
C329310                          CCGAGACTGA AATAGTTTAG GAAA
SW340                            CCGAGACTGA AATAGTTTAG GAAA
SW339                            CCGAGACTGA AATAGTTTAG GAAA
C329314                          CCGAGACTGA AATAGTTTAG GAAA
SW308                            CCGAGACTGA AATAGTTTAG GAAA
IC85                             CCGAGACTGA AATAGTTTAG GAAA
SW318                            CCGAGACTGA AATAGTTTAG GAAA
SW330                            CCGAGACTGA AATAGTTTAG GAAA
SW322                            CCGAGACTGA AATAGTTTAG GAAA
                                 ....|....| ....|....| ....
                                    845        855
SW324                            CCGAGACTGA AATAGTTTAG GAAA
SW305                            CCGAGACTGA AATAGTTTAG GAAA
SW315                            CCGAGACTGA AATAGTTTAG GAAA
SW320                            CCGAGACTGA AATAGTTTAG GAAA
SW321                            CCGAGACTGA AATAGTTTAG GAAA
SW341                            CCGAGACTGA AATAGTTTAG GAAA
IC94                             CCGAGACTGA AATAGTTTAG GAAA
IC92                             CCGAGACTGA AATAGTTTAG GAAA
IC86                             CCGAGACTGA AATAGTTTAG GAAA
SW306                            CCGAGACTGA AATAGTTTAG GAAA
SW316                            CCGAGACTGA AATAGTTTAG GAAA
IC90                             CCGAGACTGA AATAGTTTAG GAAA
SW313                            CCGAGACTGA AATAGTTTAG GAAA
SW317                            CCGAGACTGA AATAGTTTAG GAAA
SW325                            CCGAGACTGA AATAGTTTAG GAAA
C329318                          CCGAGACTGA AATAGTTTAG GAAA
N666251                          CCGAGACTGA AATAGTTTAG GAAA
IC87                             CCGAGACTGA AATAGTTTAG GAAA
SW319                            CCGAGACTGA AATAGTTTAG GAAA
SW307                            CCGAGACTGA AATAGTTTAG GAAA
N666252                          CCGAGACTGA AATAGTTTAG GAAA
SW360                            CCGAGACTGA AATAGTTTAG GAAA
SW346                            CCGAGACTGA AATAGTTTAG GAAA
IC82                             CCAAGACTGA AATAGTTTAG GAAA
SW328                            CCAAGACTGA AATAGTTTAG GAAA
IC84                             CCGAGACTGA AATAGTTTAG GAAA
SW329                            CCGAGACTGA AATAGTTTAG GAAA
IC83                             CCGAGACTGA AATAGTTTAG GAAA
SW304                            CCAAAACTGA AATAGTTTAG GAAA
R025406                          CCAAAACTGA AATAGTTTAG GAAA
R686358                          CCGACATTGA AATAGTAAAG GAAA
N666253                          CCAAGACTGA AATAGTTTAG GCAA
IC89                             CCAAGACTGA AATAGTTTAG GCAA
R686363                          CCAAAACTGA AATAGTTTAG GAAA
IC93                             TCGAGACTGA AATAGTTTAG GCAA
SW332                            TCGAGACTGA AATAGTTTAG GCAA
R686361                          GCAAGTCTGA AATAGATTAG CAAA
F149909                          CCAAGACTGA AATGGTTTAG GAAA
IC91                             CCGAAACTGA AATAGTTGAG GAAA
Q286371                          CCGAGACTTA AA-AGTTTAG GAAA
F149915                          CCTAGGCCGA AAGGCCAAGG AAAG
B445018                          CCTACTCCGA AAGGAGATGG AAAA
F149910                          CCGATAATTA AA-ATTGGAG GAAA
F149907                          CCGAAACCGT CA-GGTGGAG GAAA
X027294                          ACAAATTTGA AAAAATAAGT AAAA
Q286372                          ACTAATCTGA AAGATAAGT AAAA
F149913                          CCTATTTTGA AAAAAGGAGG AAAT
Y769863                          CCGATATTTA AAAACGGAGG AAAA
F149908                          CCAATATTGA AAAGTGGAGG GAAA
onsensus                           * *        *          *
```
