# Supplementary material for: First Evidence of Entamoeba Parasites in Australian Wild Deer and Assessment of Transmission to Cattle
Source: Front Cell Infect Microbiol. 2022 Jun 10;12:883031. doi: 10.3389/fcimb.2022.883031 (PMC9226911; doi:10.3389/fcimb.2022.883031)
Supplement: Supplementary Table 1 — List of 18S rRNA Entamoeba sequences included on the phylogenetic and divergence time analysis. [file Table_1.docx]

**Table S1.**

| *Entamoeba* species | GenBank accession number | Host | Geographic location |
| --- | --- | --- | --- |
| *E. muris* | AB445018 | Mongolian gerbil | Japan |
| *E. moshkovskii* | AF149906 | ND | N/A |
| *E. hartmanni* | AF149907 | Primate | N/A |
| *E. ranarum* | AF149908 | Amphibian | N/A |
| *E. insolita* | AF149909 | Tortoise | N/A |
| *E. terrapinae* | AF149910 | Turtle | N/A |
| *E. polecki* | AF149913 | Pig | N/A |
| *E. coli* | AF149915 | Primate | N/A |
| *E. invadens* | AY769863 | Turtle | N/A |
| *E. equi* | DQ286371 | Horse | N/A |
| *E. suis* | DQ286372 | Pig | N/A |
| *E. ecuadoriensis* | DQ286373 | Enviromental sample | N/A |
| *E. bovis* | FN666249 | Cattle | Sweden |
| *E. bovis* | FN666250 | Sheep | Sweden |
| *E. bovis* | FN666251 | Sheep | Sweden |
| *E. bovis* | FN666252 | Reindeer | Iceland |
| *Entamoeba sp. RL1* | FN666253 | Roe deer | Sweden |
| *E. nuttalli* | FR686356 | Primate | France |
| *Entamoeba sp. RL3* | FR686358 | Hulman | N/A |
| *Entamoeba sp. RL4* | FR686361 | Cattle | N/A |
| *Entamoeba sp. RL2* | FR686363 | Cattle | N/A |
| *Entamoeba sp. RL8* | KR025406 | Cattle | United Kingdom |
| *E. bangladeshi* | KR025411 | Human | Bangladesh |
| *E. gingivalis* | KX027294 | Human | Mexico |
| *E. bovis* | KY012746 | Goat | Australia |
| *E. bovis* | KY012748 | Goat | Australia |
| *E. bovis* | LC329307 | Cattle | Japan |
| *E. bovis* | LC329308 | Cattle | Japan |
| *E. bovis* | LC329316 | Cattle | Japan |
| *E. bovis* | LC329318 | Cattle | Japan |
| *E. histolytica* | X64142 | Human | N/A |

N/A: not available
