# Supplementary material for: First Evidence of Entamoeba Parasites in Australian Wild Deer and Assessment of Transmission to Cattle
Source: Front Cell Infect Microbiol. 2022 Jun 10;12:883031. doi: 10.3389/fcimb.2022.883031 (PMC9226911; doi:10.3389/fcimb.2022.883031)
Supplement: Supplementary Table 2 — List of accession number of the Entamoeba 18S rRNA DNA sequences obtained in the present study and submitted to GenBank. [file Table_2.docx]

**Table S2.**

| **Sample ID** | **host** | **Accession number** |
| --- | --- | --- |
| LN2 | cattle | OM415364 |
| LN3 | cattle | OM415365 |
| LN4 | cattle | OM415366 |
| LN5 | cattle | OM415367 |
| LN6 | cattle | OM415368 |
| LN7 | cattle | OM415369 |
| LN8 | cattle | OM415370 |
| LV3 | cattle | OM415371 |
| LV5 | cattle | OM415372 |
| LV7 | cattle | OM415373 |
| LV9 | cattle | OM415374 |
| LV10 | cattle | OM415375 |
| LV14 | cattle | OM415376 |
| NSW304 | fallow deer | OM415377 |
| NSW305 | fallow deer | OM415378 |
| NSW306 | fallow deer | OM415379 |
| NSW307 | fallow deer | OM415380 |
| NSW308 | fallow deer | OM415381 |
| NSW312 | fallow deer | OM415382 |
| NSW313 | fallow deer | OM415383 |
| NSW315 | fallow deer | OM415384 |
| NSW316 | fallow deer | OM415385 |
| NSW317 | fallow deer | OM415386 |
| NSW318 | fallow deer | OM415387 |
| NSW319 | fallow deer | OM415388 |
| NSW320 | fallow deer | OM415389 |
| NSW321 | fallow deer | OM415390 |
| NSW322 | fallow deer | OM415391 |
| NSW324 | fallow deer | OM415392 |
| NSW325 | fallow deer | OM415393 |
| NSW326 | fallow deer | OM415394 |
| NSW327 | fallow deer | OM415395 |
| NSW328 | fallow deer | OM415396 |
| NSW329 | fallow deer | OM415397 |
| NSW330 | fallow deer | OM415398 |
| NSW331 | fallow deer | OM415399 |
| NSW332 | fallow deer | OM415400 |
| VIC82 | fallow deer | OM415401 |
| VIC83 | fallow deer | OM415402 |
| VIC84 | fallow deer | OM415403 |
| VIC85 | sambar deer | OM415404 |
| VIC86 | sambar deer | OM415405 |
| VIC87 | sambar deer | OM415406 |
| VIC88 | sambar deer | OM415407 |
| VIC89 | sambar deer | OM415408 |
| LN1 | cattle | OM415409 |
| NSW339 | fallow deer | OM415410 |
| NSW340 | fallow deer | OM415411 |
| NSW341 | fallow deer | OM415412 |
| NSW343 | fallow deer | OM415413 |
| NSW344 | fallow deer | OM415414 |
| NSW345 | fallow deer | OM415415 |
| NSW346 | fallow deer | OM415416 |
| NSW349 | fallow deer | OM415417 |
| NSW350 | fallow deer | OM415418 |
| NSW360 | fallow deer | OM415419 |
| VIC90 | sambar deer | OM415420 |
| VIC91 | sambar deer | OM415421 |
| VIC92 | sambar deer | OM415422 |
| VIC93 | fallow deer | OM415423 |
| VIC94 | fallow deer | OM415424 |
