# Supplementary material for: First Evidence of Entamoeba Parasites in Australian Wild Deer and Assessment of Transmission to Cattle
Source: Front Cell Infect Microbiol. 2022 Jun 10;12:883031. doi: 10.3389/fcimb.2022.883031 (PMC9226911; doi:10.3389/fcimb.2022.883031)
Supplement: Supplementary file 7 [file Table_3.docx]

**Table S3.**

| **Primer name** | **Sequence (5′ to 3′)** | **Annealing Temperature (°C)** |
| --- | --- | --- |
| A-L5 | GGATCGATACCCCTCATCTCCA | 64 |
| A-L3 | CGCATCTTGCGATAGCCGAG |  |
| D-A5 | CTGGTTAGTATCTTCGCCTGT | 56 |
| D-A3 | GCTACACCCCCATTAACAAT |  |
| N-K5 | CGAACGGCTGTTAACCGTTA | 55 |
| N-K3 | TTCCTAGCTCAGTCGGTAGA |  |
| R-R5 | AGCATCAGCCTTCTAAGCTG | 55 |
| R-R3 | CTTCCGACTGAGCTAACAAG |  |
| STGA-D5 | CTCTGGATGCGTAGGTTCAA | 58 |
| STGA-D3 | GTATCTTCGCCTGTCACGTG |  |
| S-Q5 | GTGGTCTAAGGCGTGTGACT | 56 |
| S-Q3 | GAGATTCTGGTTCTTAGGACCC |  |
